# Characterization of liver, adipose, and fecal microbiome in obese patients with MASLD: links with disease severity and metabolic dysfunction parameters

**DOI:** 10.1186/s40168-024-02004-7

**Published:** 2025-01-14

**Authors:** Katherine J. P. Schwenger, Julia K. Copeland, Yasaman Ghorbani, Lina Chen, Elena M. Comelli, David S. Guttman, Sandra E. Fischer, Timothy D. Jackson, Allan Okrainec, Johane P. Allard

**Affiliations:** 1 Toronto General Hospital, University Health Network, Toronto, Canada; 2 Centre for the Analysis of Genome Evolution & Function, University of Toronto, Toronto, Canada; 3 Sunnybrook Health Sciences Centre, University of Toronto, Toronto, Canada; 4 Department of Laboratory Medicine and Pathobiology, University of Toronto, Toronto, Canada; 5 Department of Nutritional Sciences, University of Toronto, Toronto, Canada; 6 Division of General Surgery, University of Toronto, Toronto, Canada; 7 Division of General Surgery, Toronto Western Hospital, University Health Network, Toronto, Canada; 8 Division of Gastroenterology, Department of Medicine, Toronto General Hospital, 585 University Avenue, 9N-973, Toronto, ON M5G 2N2 Canada

**Keywords:** Fatty liver, Tissue microbiome, Hepatic immune cells, Hepatic transcriptome, Adipose gene expression

## Abstract

**Background:**

Metabolic dysfunction-associated steatotic liver disease (MASLD) encompasses a range of histological findings from the generally benign simple steatosis to steatohepatitis (MASH) which can progress to fibrosis and cirrhosis. Several factors, including the microbiome, may contribute to disease progression.

**Results:**

Here, we demonstrate links between the presence and abundance of specific bacteria in the adipose and liver tissues, inflammatory genes, immune cell responses, and disease severity. Overall, in MASLD patients, we observed a generalized obesity-induced translocation of gut bacteria to hepatic and adipose tissues. We identified microbial patterns unique to more severely diseased tissues. Specifically, *Enterococcus*, *Granulicatella*, and Morganellaceae abundance is positively correlated with immune cell counts and inflammatory gene expression levels, and both genera are significantly enriched in MASH patients. *Brevibacterium* is enriched in adipose tissues of patients with liver fibrosis.

**Conclusion:**

Together, these results provide further insight into the microbial factors that may be driving disease severity.

Video Abstract

**Supplementary Information:**

The online version contains supplementary material available at 10.1186/s40168-024-02004-7.

## Introduction

Metabolic dysfunction-associated steatotic liver disease (MASLD) is the new term for non-alcoholic fatty liver disease (NAFLD) and is defined by the American Association for the Study of Liver Disease (AASLD) as the presence of hepatic steatosis (> 5% fat) and the finding of any cardiometabolic risk factor [1]. The presence of liver steatosis (> 5% fat) for the diagnosis of MASLD can be assessed by imaging like a CT scan or ultrasound. On the other hand, a liver biopsy is required to specifically assess the presence of ballooning of hepatocytes, for the diagnosis of steatohepatitis (MASH) and confirmed fibrosis severity [1, 2]. According to the AASLD guidance [1], the term MASLD includes MASH, which is a more severe form, while MASLD refers only to the presence of steatosis. However, overall, a proportion of these patients can have various degrees of fibrosis which can lead to cirrhosis and detrimental outcomes [2]. Therefore, patients generally diagnosed as MASLD can also progress to more severe liver disease. Globally, MASLD is increasing with MASH cirrhosis trending to be the primary reason for liver transplantation by 2030 [3]. MASLD and MASH are both associated with features of metabolic syndrome, including being overweight or obese, having insulin resistance, type 2 diabetes (T2D), and/or high levels of triglycerides in the blood [4]. However, not all individuals who have MASLD will develop inflammation (MASH) and/or fibrosis and progress to MASH cirrhosis, thus suggesting other mechanisms are involved in disease pathogenesis.

Studies have suggested a role for the intestinal microbiome (IM) in features of metabolic syndrome. For instance, IM has been linked to T2D and bacteria such as lower *Faecalibacterium prausnitzii*, and higher *Bacteroides vulgates* have been observed in those with T2D compared to control [5]. In addition, specific microbiome signatures for liver steatosis and fibrosis, independent of other metabolic risk factors, were reported in patients with T2D which can be related to increased intestinal permeability via gut-barrier dysfunction [6, 7]. This in turn can lead to translocation of endotoxins, bacterial fragments, and potentially live bacteria into the circulatory system and extra-intestinal tissue [8, 9]. The liver, which receives approximately 75% of its portal blood supply from the intestine [10], is one of the first targets of circulatory bacteria/fragments, but very few studies reported on liver tissue bacteria in MASLD [11, 12]. In one study [11], hepatic bacterial composition was shown to differ between morbidly obese and non-morbidly obese individuals with MASLD compared to controls but also across the histological spectrum [11]. Specifically, Bacteroidetes and Firmicutes were overrepresented in the liver of morbidly obese patients, whereas Proteobacteria was overrepresented in the non-morbidly obese group [11]. In both groups, there was a depletion of the Lachnospiraceae family associated with severe histological features and additionally, various associations between worsening liver histology and intrahepatic Peptostreptococcaceae, Verrucomicrobia, Actinobacteria, and Gammaproteobacteria DNA among morbidly obese patients [11]. Another study assessing blood, fecal, and liver microbiome in those with either hepatocellular carcinoma or nonmalignant cirrhotic patients or non-cirrhotic MASLD found higher blood and liver Bacteroidaceae and Ruminococcaceae and lower fecal *Agathobacter* and *Blautia* [12]. However, these studies did not investigate links with metabolic dysfunction parameters including gene expression and immune cells that may play a role in MASLD pathogenesis [13–16]. Additionally, it did not assess adipose tissue.

Fecal bacteria can translocate into adipose tissue and affect metabolism. In 75 obese subjects, bacterial DNA was found in omental, subcutaneous, and visceral adipose tissue [17], where Proteobacteria and Firmicute*s* were the predominant phyla in these tissues and bacterial load being associated with adipose immune cell infiltration and inflammatory parameters [17]. Higher bacterial load was also found in liver and omental adipose tissue, but not plasma, of 40 morbidly obese individuals [9]. Those with T2D had lower bacterial diversity with reduced Gram-positive bacteria (*Faecalibacterium*) and higher levels of Enterobacteriaceae in both adipose tissue and plasma. These studies did not assess MASLD [9, 17].

Assessing links between adipose tissue bacteria and MASLD is important. Bacterial fragments identified in the visceral adipose tissue may contribute to visceral adipose tissue dysregulation via increased expression of inflammatory cytokines [17], thus possibly contributing to MASLD pathogenesis [18]. Mechanisms include alterations in adipokine levels with higher leptin and lower adiponectin, larger fat cell area, and alterations in gene expression such as TGFB1 and p53 genes [18, 19], all associated with metabolic dysfunction [19–21]. To our knowledge, no studies have investigated the relationship between IM, liver, and visceral adipose tissue bacterial composition, together with adipose tissue histology, gene expression in the liver and adipose tissues, immune cells, and other metabolic dysfunction parameters in relation with MASLD and disease severity. In the present study, we characterized the fecal, hepatic, and adipose tissue microbiome of obese patients, determined their bacterial profiles according to MASLD severity and liver fibrosis, and linked these findings with metabolic dysfunction parameters, liver immune cells, and adipose/hepatic gene expression.

## Materials and methods

### Study design

This is a prospective study which included subjects with obesity (BMI ≥ 35 kg/m^2^) who underwent bariatric surgery between September 2013 and August 2020. Patients were screened and recruited from a University Hospital’s bariatric clinic prior to surgery. Further information, including inclusion and exclusion criteria, can be found in Supplementary.

All subjects provided written consent and data for clinical variables. Samples were collected just before or at the time of bariatric surgery and included fecal, liver, and adipose tissue for microbiome, tissue histology, immune cells, and gene expression. Bloodwork was performed for relevant adipokines, cytokines, gut hormones, and metabolic parameters (see methods and the list of measured parameters, their known function, and associations with MASLD/MASH in Supplementary Table 1). All research was approved by the University Health Network Research Ethics Board (UHN; REB#13–6115-A and REB#21–5623) and was conducted in accordance with both the Declarations of Helsinki and Istanbul, and they were also registered with ClinicalTrials.gov (NCT0185646).

### Clinical and biochemical measurements

Blood work was collected in the morning after a 12-h fast. The certified University Hospital Laboratory Medicine Program analyzed samples for HbA1c, fasting insulin, glucose, liver enzymes, lipid profile, albumin, and platelets using standard protocols (see Supplementary Methods).

### Liver biopsy

A wedge biopsy was performed at the time of surgery. To avoid contamination, the biopsies were transferred to a sterile petri dish. Using sterile forceps and single-use scalpel, the biopsies were divided into three smaller parts. One piece was preserved in formalin within 15 min of the biopsy and stored at 4 °C in a refrigerator and later embedded in paraffin; this sample was used to determine liver histology and immune cells. The second and third samples were transferred to sterile microtubes and kept at − 80 °C for DNA and RNA extraction. Liver histology was assessed blindly by a liver pathologist. The Brunt system [22] was used for all liver biopsies. For the grouping, normal liver was determined if < 5% steatosis and no other histologic abnormalities were found. MASLD was diagnosed if the liver had ≥ 5% steatosis. Within the MASLD group, patients who had ballooning of hepatocytes were defined as having MASH. Therefore, the term MASLD is used for the grouping of both steatotic and MASH, while the term MASH is used specifically for those who have more severe histology with ballooning of hepatocytes. Both of these histologic conditions may or may not have fibrosis. The presence and severity of fibrosis [22] were assessed: if no fibrosis, the grade was 0, and if presence of fibrosis, a fibrosis grade of 1, 2, 3 or 4 was used with severe fibrosis determined to be a fibrosis grade of 3 or 4. Details of liver immune cell, hepatic DNA extraction for liver microbiome, and hepatic RNA extraction for transcriptome can be found in the Supplementary Materials.

### Adipose biopsy

Two visceral adipose tissue samples were collected at the time of surgery, with both samples being stored at − 80 °C. A similar process to the liver biopsy was used for collection, aliquoting, and transfer of the samples to sterile microtubes. One was for RNA extraction to be used for gene expression and the other was for DNA extraction to be used for tissue microbiome, details can be found in the Supplementary Materials. Briefly, RNA was extracted from the adipose tissue using Trizol reagent combined with GeneJET RNA Purification Kit (Thermofisher Scientific Cat number #K0732) and was assessed with a quantitative polymerase chain reaction. Microbial DNA from the tissue was extracted and sequenced as described below.

### Fecal and tissue microbiome sequencing and analysis

Stool samples were collected within 72 h of the baseline visit, aliquoted and kept in a − 80 °C freezer as previously described [23]. DNA was extracted from stool using the ZymoBIOMICS DNA Miniprep kit and from adipose and liver biopsies using the ZymoBIOMICS Host-Zero microbial DNA prep kit, with the addition of the ZymoBIOMICS Low Microbial Load Spike-In Control II, following the manufacturer protocol. See Supplementary Methods.

The DNA sequencing was performed by the Centre of the Analysis of Genome Evolution and Function (CAGEF, University of Toronto, ON, Canada) as previously described [24]. The V4 region of the 16S rRNA gene was amplified and sequenced. Extraction and PCR amplification positive and negative controls were prepared alongside samples in order to identify and thus eliminate contaminant bacteria in the data. See details in Supplementary Methods. The Qiime2 analysis package version 2023.2 was used for sequence analysis. In brief, paired-end sequences were assembled and quality trimmed and clustered into Amplicon Sequence Variant (ASV) groups. Taxonomy was assigned using a trained Average ReadyToWear Silva database version 138.1. The data was then analyzed using R version 4.3.0. To ensure that results were simply not reporting contaminant sequences, prior to any analysis, the data was first rigorously filtered to remove contaminant sequences using Decontam, prevalence, and abundance filtering. The data was subsequently normalized using ConQuR to reduce batch effects, following default parameters. The estimated ASV abundances and total cells per gram of tissue were determined using the Zymo Low Microbial Load Spike-In Control, following the standard protocol. See details in Supplementary Methods.

### Statistical analysis

Descriptive statistics were presented for continuous variables as median (1st, 3rd quartile) and as frequency (percentage) for categorical variables. For all parameters, we compared the following groups: normal liver (normal liver obese: NLO) versus MASLD; NLO versus MASH and in patients with MASLD, we compared no fibrosis (F0) to presence of fibrosis (F1, F2, F3, F4); and F0 to severe fibrosis (F3, F4). The differences in anthropometric and metabolic parameters, immune cells, gut hormones, adipokines, FGF-19, endotoxin, and adipose gene expression were assessed using the Wilcoxon rank-sum test for continuous variables and chi-squared test or Fisher exact test for categorical variables. A *p*-value < 0.05 was considered statistically significant. Differential gene expression in the liver was assessed using DESeq2 in R and adjusted for multiple hypothesis testing using Benjamini-Hochberg. Genes with log2(fold change) and adjusted *p* < 0.05 were considered as differentially expressed. KEGG pathway overrepresentation analysis in the liver was performed using ClusterProfiler, and after adjustment similar to the above, pathways with adjusted *p* < 0.05 were considered significantly different between groups (details in the Supplementary Material). For the KEGG pathways in the liver, gene set variation analysis (GSVA) was also conducted using the GSVA package.

16S rRNA gene Chao1 alpha diversity was determined on Amplicon Sequence Variant (ASV) data rarefied to an even depth and compared by performing a one-way analysis of variance (ANOVA) followed by Tukey’s HSD test for post hoc comparisons. Permutational multivariate analysis of variance (PERMANOVA) using distance matrices (ADONIS) with subsequent Benjamini–Hochberg *P* value adjustment was used to assign statistical significance to the clusters of cumulative sum scored ASVs that were visualized in PCoA scatterplots. To identify bacterial signatures that were differentially abundant between tissues, disease severity, and fibrosis levels, we filtered low prevalence taxa and used Microbiome Multivariable Associations with Linear Models (MaAsLin2), applying Centred Log-Ratio (CLR) normalization followed by linear model (LM) analysis and a Benjamini–Hochberg *P* value correction. Biological relevance was investigated by comparing the MaAsLin2 results from both the relative abundance analyses and the estimated absolute abundance analyses, to identify taxa that followed similar trends. Details are in the Supplementary Material.

Lastly, significant bacteria identified in stool, liver, and adipose were correlated with significant clinical, metabolic, immune cells, adipose genes, and gene liver KEGG pathways. For the correlations, the Spearman correlation coefficient was calculated and *p* < 0.05 was considered significant. A heatmap of the correlations was also created using rstatix package. Details are in the Supplementary Material.

## Results

### MASLD/MASH and fibrosis have significantly altered metabolic parameters, immune cells, and adipose and hepatic gene expression

A total of 98 individuals with obesity (see Patient Flow Chart, Fig. S1A, B) were included in the analysis. There were 26 obese individuals with normal liver (NLO) and 72 individuals with MASLD. Of those with MASLD, 36 were diagnosed with steatotic liver and 36 with MASH. Of those, 21 did not have fibrosis and 51 had fibrosis. A summary of the clinical and biological samples taken for the study is shown in Fig. S2 with Table S1 providing additional explanations about these measurements and their relevance in MASLD and fibrosis.

Overall, and as expected, patients with MASLD or MASH showed clinical and metabolic dysfunction parameters when compared with NLO (Table S2); similar differences were also demonstrated related to the degree of fibrosis (Table S3). Additionally, in MASLD overall, or specifically in MASH, there was significantly lower plasma adiponectin and higher plasma retinol-binding protein-4 compared to NLO, and C-reactive protein was significantly higher in those with MASH, whereas fibroblast growth factor (FGF)−19 was significantly lower in those with MASLD compared to NLO. In addition, endotoxin levels were significantly higher in both MASLD and MASH compared to NLO, suggesting increased intestinal permeability and bacterial translocation.

There were significant differences in hepatic immune cells between groups (Supplementary Table S5). Those with MASLD or MASH had higher Helper T, CD4 Treg, and B cells and lower activated macrophages compared to NLO (see Supplementary Table S1 for explanation). A few adipose gene expressions were found to be significant: those with MASLD had significantly higher expression of adipose peroxisome proliferator-activated receptor gamma (PPARg), which increases adipose tissue fat storage capacity [25], and those with MASH had significantly lower expression of adipose S100a8, a calcium-binding protein which is involved in modulating inflammatory response [26], compared to NLO (Supplementary Table S4). However, there were several significant differences in hepatic gene expression between groups (Supplementary Table S6). In the liver, we identified 11 differentially expressed genes (DEG) in MASLD vs NLO and 60 in MASH vs NLO. Nine genes were common in both comparisons which were involved in lipid metabolism/transport, glucose homeostasis, and extracellular matrix degradation. Thirty-eight genes (mostly related to the immune system, cytokines/chemokines, extracellular matrix, and apoptosis) were uniquely upregulated, and 13 were uniquely downregulated in MASH vs NLO. We also identified several KEGG pathways that were significantly different (details in Supplementary Tables S10 and S11).

Those with the presence of or severe fibrosis had significantly lower expression of adipose S100a9 (Supplementary Table S7), and those with severe fibrosis had significantly lower leptin and adiponectin compared to those without fibrosis. 

There were also significant differences in hepatic immune cell and gene expression between groups (Supplementary Table S8 and S9). Those with severe fibrosis had higher Helper T and B cells and lower NK cells compared to those without fibrosis. In those with MASLD, 10 DEG were common in the presence of fibrosis vs no fibrosis and severe fibrosis vs no fibrosis. However, in severe fibrosis, 57 additional genes related to extracellular matrix formation and its proteins as well as immune system and apoptosis were upregulated compared to no fibrosis group and 10 DEG uniquely downregulated. Significant KEGG pathways can be found in Supplementary Tables S12 and S13.

### Fecal microbiome differed significantly from tissues while adipose and liver tissue microbiome were highly similar

We first assessed the abundance and diversity from all 3 sample types. Overall, the average number of amplicon sequence variants (ASV) identified after filtering out low abundance and low prevalence ASVs, removing identified contaminants, and correcting for batch effect (see Supplementary Methods for more details) was approximately 5.9 × 10^3^ for tissue samples and 2.1 × 10^4^ for stool samples in all patient data. We observed that in the stool, the most abundant average genus across all samples was *Blautia*, followed by *Faecalibacterium*, *Agathobacter*, *Collinsella*, *Eubacterium*, *Dorea*, *Fusicatenibacter*, *Anaerostipes*, *Subdoligranulum*, *Ruminococcus*, *Streptococcus*, *Bifidobacterium*, *Coprococcus*, and *Dialister*, all with an average relative abundance (RA) > 1%.

In the tissue, we observed the most abundant genera for both adipose and liver tissues were *Corynebacterium*, followed by *Bacillaceae* sp., *Pseudomonas*, *Bacillus*, *Halomonas*, and *Streptococcus*, all with an average RA > 1%. Between the adipose and liver tissues, 83% of the total genera are shared (identified in one or more samples within each tissue type). Stool samples shared only 33% and 37% of genera with adipose and liver tissues, respectively (Supplementary Figure S3A). Thirty-two percent of these genera were shared between all sample types: adipose, liver, and stool. Less than 1% of genera were shared between adipose and stool only, and 5% between liver and stool only. The distribution of genera with a prevalence > 10% and an abundance > 0.1% (therefore excluding extremely low abundance and low prevalence genera) were compared between hepatic, adipose, and fecal samples (Supplementary Figs. S3B–S2E). Trends were consistent for NLO, MASLD, and MASH samples, when examined separately (Supplementary Fig.S3C–E).

**Fig. 1:**
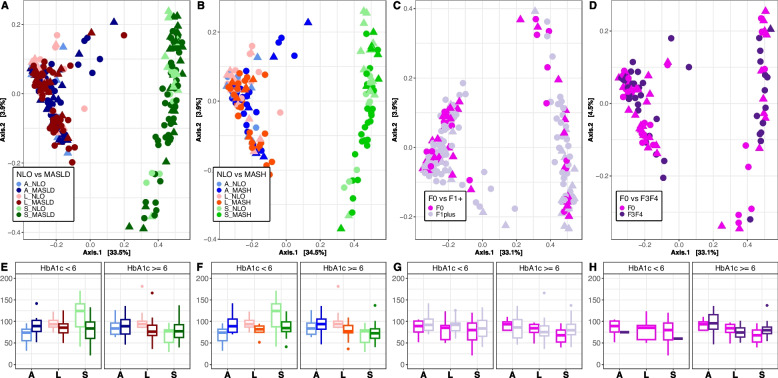
Bray–Curtis PCoAand Chao1 alpha diversity plots of stool, adipose, and liver tissues. **A**–**D** PCoA plots of Bray–Curtis distances for stool, adipose, and liver tissues and corresponding **E**–**H** Chao1 alpha diversity values. Data is colored by disease state **A**, **E** NLO compared to MASLD, and **B**, **F** NLO compared to MASH, and fibrosis level **C**, **G** no fibrosis (F0) compared to the presence of fibrosis (F1, F2, F3, F4), and **D**, **H** no fibrosis (F0) compared to severe fibrosis (F3, F4). The shape of point indicates HbA1c < 6 (triangle) or HbA1c > = 6 (circle). A adipose, L liver, and S stool. Diagnoses: F0 no fibrosis, F1 + presence of fibrosis, F3F4 severe fibrosis

Our primary goal was first to determine whether the microbial communities were influenced by sample type, disease status, fibrosis level, or HbA1c level. HbA1c was input into the model prior to the factor of interest to control for the potential confounding effect of T2D [6]. This was performed to examine these factors sequentially, first determining the amount of variation explained by HbA1c and then the residual variation explained by the factor of interest. We determined that the fecal microbiome differed significantly from their corresponding hepatic and adipose tissue microbial communities, regardless of disease grouping or HbA1c level (see Fig. 1A–D). Bray–Curtis diversity ADONIS results indicate that sample type (fecal, hepatic, adipose) accounts for the majority of the variance observed between samples (*p* < 0.01, *R*2 = ~ 0.32) (Supplementary Table S14). When considering all sample types, disease state (NLO vs MASLD, or NLO vs MASH) (Fig. 1A, B) and fibrosis level in those with MASLD (no versus presence of fibrosis or no versus severe fibrosis) (Fig. 1C, D) did not contribute significantly to sample diversity and accounted for a very small proportion (*R*2 between 0.01 and 0.02) of the variance. HbA1c level also did not contribute to significant differences in community diversity between samples and did not interact significantly with either disease state or fibrosis level. Alpha diversity was also compared between disease states, fibrosis levels, and HbA1c levels. A significant difference in alpha diversity was identified between NLO and MASLD (Fig. 1E) and between NLO and MASH (Fig. 1F) for the liver (Chao1 *p* < 0.05) (Supplementary Table S15).

Using rarefied data, a significant interaction was identified between MASLD, MASH, and HbA1c levels in liver alpha diversity levels. For observation, HbA1c levels were grouped as either less than 6 or greater than or equal to 6, as per the Health Canada guidelines for the presence of pre-diabetes and diabetes [27]. We observed that alpha diversity was significantly lower in MASLD and MASH livers, particularly for individuals with higher HbA1c levels. In those with MASLD, no difference was observed between no fibrosis vs presence or severe fibrosis for stool or tissues (see Fig. 1G and H).

Differential abundance analysis was performed using MaAsLin2 to compare the relative abundances of genera between stool and tissue samples (minimum prevalence set at 10%, CLR normalization, see Supplementary methods for more details). Overall, 94% of genera differed significantly between stool and tissues (adjusted *p* < 0.05). The genera with the most substantial differences included *Pseudomonas, Corynebacterium, Halomonas*, and *Bacillaceae*, which were significantly enriched in tissue samples, and *Blautia, Anaerostipes, Ruminoccus, Butyricicoccus*, and *Eubacterium,* which were significantly enriched in stool (Supplementary Table S16).

The data was then separated to analyze the tissue and stool data independently. In the preliminary analysis, we compared simple steatosis to MASH and did not find an overall significant difference between the two groups (data not shown). For stool alone, disease state and fibrosis level did not significantly affect community diversity (Supplementary Fig. S4A–4D, Supplementary Table S17). For the liver and adipose tissues, we observe that tissue type, MASLD, and MASH have a small but significant impact on community structure after accounting for the effect of HbA1c on community diversity (ADONIS: *p* < 0.01, *R*2 = ~ 0.02 for each parameter) (Supplementary Table S18, Supplementary Fig. S5E and S5F). In those with MASLD, the presence of fibrosis and severe fibrosis also had a significant impact on community structure (ADONIS: *p* < 0.01, *R*2 = 0.02 and *p* < 0.01, *R*2 = 0.04, respectively) (Supplementary Fig. S5G and 4H). It is important to note that intra-patient consistency also contributed significantly and accounted for the greatest proportion of variance observed between samples, as both adipose and liver tissues from the same patient are examined within this analysis (ADONIS: *p* < 0.05, *R*2 = ~ 0.5).

Differential abundance analysis was performed to determine differences in stool genera relative abundances between the disease states and fibrosis groups of interest. Age, sex, and HbA1c level were considered cofactors. Taxa that were only influenced by age or sex were filtered out from the analysis. We did not observe any genera to be significantly different between the groups of interest.

We investigated the MaAsLin2 Log2 fold change of genera that differed within stool between these comparisons of interest, despite not reaching significance (Supplementary Fig. S6A–H). Taxa influenced by either worsening disease state or fibrosis level or increasing HbA1c level were first reported (Supplementary Fig. S6A–S6D). After removing taxa that were affected by increasing HbA1c level, we observed that for MASH and MASLD, the genera that showed the greatest positive LFC compared to NLO were Clostridia group Christensenellaceae R-7, Erysipelotrichaceae UCG-003, and Clostridia group CAG-352 (Supplementary Fig. S6E-F). *Limosilactobacillus* showed the greatest positive LFC between no fibrosis and severe fibrosis (Supplementary Fig. S6H).

### MASLD severity may be linked to the translocation of certain core bacteria from the fecal microbiome to the tissue

**Fig. 2:**
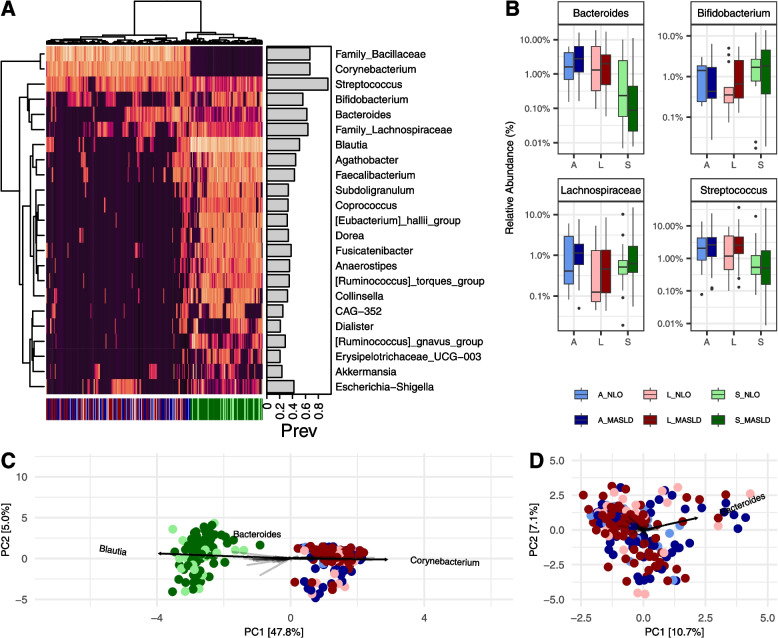
Compositional taxonomic similarities between stool, adipose, and liver tissues. **A** Euclidean clustered heatmap of the Centred Log-Ratio (CLR) values of the genera with an average relative abundance > 1% and an average prevalence > 5%. Clustering shows a separation of samples by stool and tissues on the *x*-axis cladogram and by taxonomic relative abundances on the *y*-axis cladogram. The prevalence of each genus is shown in the barplot as a proportion of 1. **B** *Bacteroides, Bifidobacterium, Lachnospriaceae,* and *Streptococcus*, which form a clade in the heatmap (**A**). **C**, **D** Principal component analysis (PCA) plots of these CLR values for **C** stool and tissues and **D** tissues alone. The taxa which contributed most significantly to the construction of the principal components are plotted on the PCAs. Data is colored by sample type and disease state (NLO and MASLD), as shown below **B**. A adipose, L liver, and S stool

To better understand taxa that were identified in both stool and tissue samples, we created a Euclidean clustered heatmap of the Centred Log-Ratio (CLR) values of the genera with an average relative abundance > 1% and an average prevalence > 5% (see Fig. 2A), as a means of understanding the core taxa shared between the gut and the tissues. Euclidean clustering shows clear segregation of stool from tissues, as was previously observed in the PCoA plot (see Fig. 1A–D). Despite an overall high degree of shared genera between stool and tissue samples, from the heatmap, we observe that this is often due to a few select samples containing certain shared taxa. For example, we observe that *Blautia*, the most abundant overall genera in stool, can be identified in only a few select tissue samples. As previously reported, only 11.2% of genera with a higher prevalence and abundance were found to be shared between the liver, adipose, and stool samples (Supplementary Fig. S3B).

However, *Streptococcus*, *Bifidobacterium*, *Bacteroides*, and Lachnospiraceae (unknown genus) form a clade of taxa that are observably present in many stool and tissue samples. *Streptococcus* is the most prevalent taxon across all stool and tissue samples. These taxa exhibited a large range of relative abundances between fecal, liver, and adipose tissues, and between disease states (see Fig. 2B). Of note, *Bacteroides* is observably less abundant in the stool microbiome of individuals with MASLD while being more abundant in the adipose and liver tissue compared to NLO. In patients that contained these taxa, it was often the case that these taxa were present in stool and both tissue types (Supplementary Fig. S7). However, we also observed that for several patients, the taxa were only observed in either adipose or liver, and stool, and were absent from the other tissue type. All four of these taxa are more abundant in the liver tissue of individuals with MASLD compared to NLO (see Fig. 2B). Next, we used an unconstrained PCA plot of these CLR values and determine which taxa are primarily responsible for community structure (see Fig. 2C). Expectedly, we observe that the stool primarily differs from the tissue due to the abundance of *Blautia*, while the tissues differ from the stool due to the abundance of *Corynebacterium* (separating on the primary PC1 axis). The relative abundance of *Bacteroides* appears to have an influence on the community composition of both stool and tissue samples, as the vector influences the position of the sample on the secondary PC2 axis. By plotting the tissue samples independently (see Fig. 2D), we observe that the abundance of *Bacteroides* is the greatest contributing factor responsible for the variation observed between tissue samples.

### Absolute abundance tissue microbiome structure associated with MASLD/MASH and fibrosis severity

The data was then further analyzed to estimate the absolute bacterial abundances in liver and adipose tissues. Due to significantly elevated levels of plasma endotoxin in MASLD and MASH patients (Supplementary Table S4), we hypothesized that bacterial translocation into adipose and hepatic tissues may be more relevant than stool bacteria in MASLD development and progression. Furthermore, we hypothesize that estimated absolute abundances may be more informative when examining communities with low bacterial biomass, such as these tissues [28, 29]. We analyzed the data, again first by correcting for batch effect and then scaling the relative abundances of each taxon by the estimated total number of cells, determined by the utilization of the ZymoBIOMICS Spike-In control II (see Methods). The estimated number of bacterial cells per gram of tissue was significantly lower in adipose tissues compared to the liver (Supplementary Fig. S8) but was not significantly affected by disease state or fibrosis level for either tissue type.

**Fig. 3:**
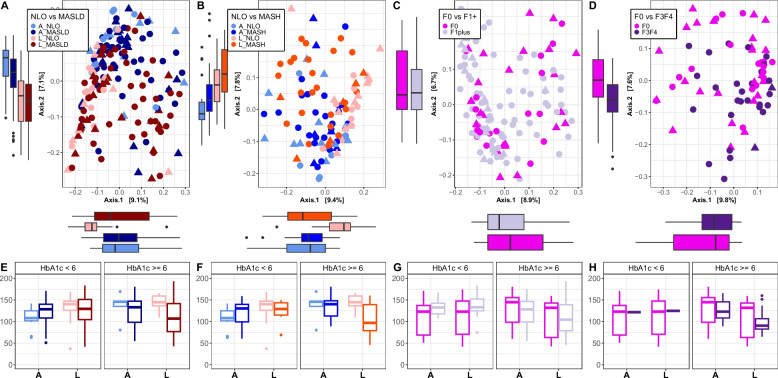
Bray–Curtis PCoAand Chao1 alpha diversity plots of adipose and liver tissues. **A**–**D** PCoA plots of Bray–Curtis distances for adipose and liver tissues and corresponding **E**–**H** Chao1 alpha diversity values. Data is colored by disease state **A**, **E** NLO compared to MASLD, and **B**, **F** NLO compared to MASH, and fibrosis level **C**, **G** no fibrosis (F0) compared to presence of fibrosis (F1, F2, F3, F4), and **D**, **H** no fibrosis (F0) compared to severe fibrosis (F3, F4). A adipose, L liver, and S stool. Diagnoses: F0 no fibrosis, F1 + presence of fibrosis, F3F4 severe fibrosis. The shape of point indicates HbA1c < 6 (triangle) or HbA1c > = 6 (circle)

Bray–Curtis distance PCoA plots (see Fig. 3A–D) and Choa1 alpha diversity indices (see Fig. 3E–H) were determined for this scaled estimated abundance data. Tissue type as well as all groups of interest (MASLD, MASH, and presence and severe fibrosis in those with MASLD) all had a significant impact on Bray–Curtis beta diversity after accounting for the effect of HbA1c (ADONIS: *p* < 0.01) (Fig. 3A–D; Supplementary Table S19). Dispersion analysis showed that beta diversity distances were significantly larger within MASLD and MASH. This suggests that the source of variance between NLO and MASLD/MASH is due to the higher level of sample dissimilarity within MASLD and MASH groups (*p* < 0.01).

Similar to the unscaled relative abundance data, but more pronounced with the estimated absolute microbiome abundances, individuals with severe fibrosis accounted for the greatest proportion of variance (*p* < 0.01, *R*2 = 0.03), more so than the presence of fibrosis alone or the diagnosis of MASLD/MASH. Observably, the data remained relatively unclustered by tissue type, disease state, and fibrosis level, as there remained to be a high degree of variability and heterogeneity between all samples and a low amount of variance explained by these factors. Alpha diversity was significantly lower in the livers of MASLD (see Fig. 3E) and MASH (see Fig. 3F.) compared to NLO (Chao1 *p* < 0.05) (Supplementary Table S20). HbA1c did not have a significant effect on alpha diversity and did not significantly interact with disease state or fibrosis level. No significant difference in alpha diversity was identified between any disease or fibrosis groups in adipose tissue.

Dirichlet-Multinomial modeling was used to cluster scaled sample data by estimated community membership, factoring in genera with a minimum total abundance > 0.5% and a prevalence > 5%. Laplace approximation suggests the probability of three metacommunities observed in the data. We observe that each subcommunity, or enterotype, is characterized primarily by the most abundant genera within the tissues: *Corynebacterium*, Bacillaceae spp.,* Pseudomonas*, and *Streptococcus* (Supplementary TableS21). *Corynebacterium* and Bacillaceae spp. are found only in tissue samples, while *Pseudomonas* and *Streptococcus* were also found in stool. *Streptococcus* was observed with an average abundance of > 1% in stool and was highly prevalent in tissues, suggesting a high degree of translocation(see Fig. 2A).

### Several taxa were significantly enriched in severely diseased or fibrotic adipose and liver tissues

**Fig. 4:**
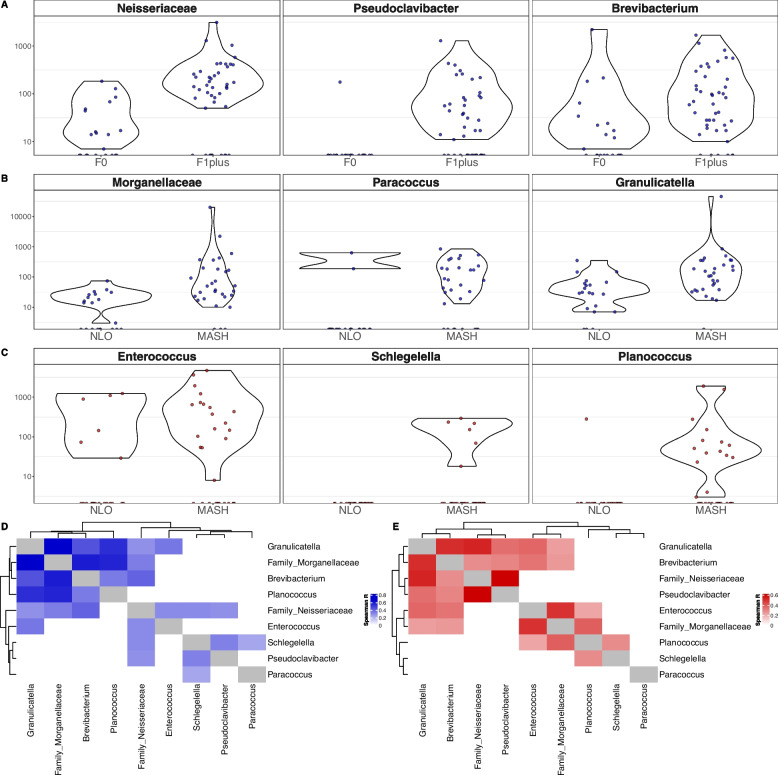
Differentially abundant genera between disease states and fibrosis levels for adipose and liver tissues. **A**–**C** The estimated scaled abundance of significant taxa (MaAsLin2 adjusted *p*-value < 0.05) that were also confirmed by the unscaled relative abundance data comparisons: **A**, **B** genera that are significantly enriched for adipose (blue) in **A** the presence of fibrosis (F1, F2, F3, F4) and in **B** MASH compared to NLO, and for **C** liver (red) genera that are significantly enriched in MASH compared to NLO. No genera were identified as significantly enriched in severe fibrosis (F3, F4) compared to no fibrosis (F0), or in MASLD compared to NLO. **D**, **E** Spearman correlation heatmaps of genera scaled abundances, for taxa identified as significant in MaAsLin2 (A-C), filtered to include only significant correlations (*p* < 0.05) for **D** adipose (blue) and **E** liver (red) tissues

Differential abundance analysis was performed to characterize the relationship between disease state and fibrosis level and the estimated abundance of each genera, as we hypothesize that the proliferation of certain taxa in the tissues may be contributing to the progression of the disease. Age, sex, and HbA1c level were considered cofactors. We focused the analysis on the estimated absolute abundance data, examining taxa that were found to be more abundant in a more advanced disease state, relative to their comparator (see Fig. 4, Supplementary Fig. S11 and S12, Supplementary Table S23). As a means of comparison, we also ran the analysis on unscaled relative abundances (Supplementary Fig. S9 and S10, Supplementary Table S22). This allowed us to identify common trends and avoid drawing conclusions from potentially overfitted estimated abundance data.

For the purpose of this study, we focused our analysis on taxa that were enriched in advanced disease states or fibrosis levels. As before, these taxa were first filtered to remove any that were only associated with age or sex. Taxa influenced by either worsening disease state or fibrosis level or increasing HbA1c level were first reported (Supplementary Fig. S9A–S9D, S10A–S10D, S11A–S11D, S12A–S12D). After removing taxa that were affected by increasing HbA1c level, we then examined taxa that were only affected by worsening disease state or fibrosis level (Supplementary Fig. S9E–S9H, S10E–S10H, S11E–S11H, S12E–S12H). This allowed the analysis to focus on taxa that were influenced by worsening disease state or fibrosis level and not confounded by increasing HbA1c levels. Of these taxa, we retained those for further analysis (Fig. 4) that were additionally identified as enriched in advanced disease state or fibrosis level in the unscaled data analysis (Supplementary Table 22, Supplementary Fig. S9E–S9H, S10E–S10H).

 Neisseriaceae (unknown genus), *Pseudoclavibacter*, and *Brevibacterium* were significantly enriched in the adipose presence of fibrosis (see Fig. 4A). Morganellaceae (unknown genus), *Paracoccus*, and *Granulicatella* were significantly enriched in adipose MASH (see Fig. 4B). *Enterococcus*, *Schlegelella*, and *Plancococcus* were significantly enriched in liver MASH (see Fig. 4C). Of these taxa, *Enterococcus* and Neisseriaceae were also identified in stool. *Enterococcus* was identified in multiple stool samples at a relative abundance > 1%.

A correlation analysis was run to compare the estimated absolute abundances of these significantly associated taxa, to better understand the community dynamics and potential co-occurrence of these taxa relative to tissue type. A heatmap was generated for adipose (see Fig. 4D) and liver (see Fig. 4E) of significant Spearman correlation indices (*p*-value < 0.05, *R*-value reported in the heatmap). We observed that for adipose tissue, the abundance of Morganelleaceae, *Brevibacterium*, *Granulicatella*, and *Planococcus* are significantly positively correlated with each other. In the liver tissue, we observed that Morganellaceae and *Enterococcus* were highly correlated, as well as with many other taxa. Interestingly, all taxa identified as significant in Fig. 4 are found in both adipose and liver tissues.

### Significant fecal/adipose/liver bacteria were associated with significant clinical, metabolic, immune cells, and gene expression parameters

**Fig. 5:**
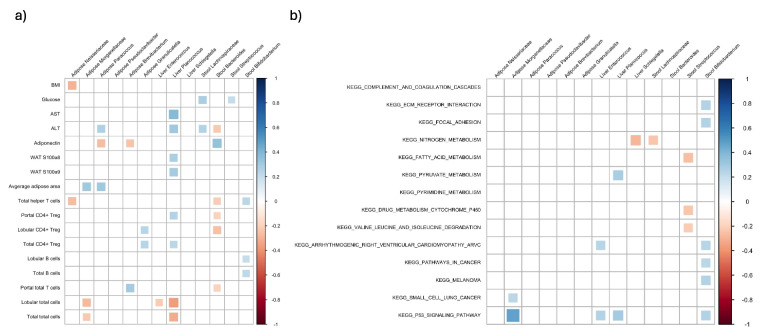
Association between significant fecal/adipose/liver bacteria and **A** clinical/immune cell and adipose gene expression, **B** significant KEGG transcriptome pathways in the liver. Correlations were assessed using the Spearman correlation. Only significant correlations (*p* < 0.05) are shown

We next looked at the association between taxa found to be of significance based on the above analyses or identified as potentially translocated taxa from stool and parameters that were significantly different between subject groupings (Fig. 5A). The taxa were Neisseriaceae, Morganellaceae, *Pseudoclavibacter*, *Granulicatella*, *Paracoccus*, and *Brevibacterium* in the adipose tissue; *Enterococcus*, *Planococcous*, and *Schlegelella* in the liver; and Lachnospiraceae, *Bacteroides*, *Streptococcus*, and *Bifidobacterium* in the stool. For this purpose, we assessed the correlation in the entire study population of NLO and MASLD patients. We found a significant negative correlation between adipose *Paracoccus* and *Brevibacterium* and plasma adiponectin. We also found a positive correlation between liver *Planococcus*, and the lobular and portal CD4 + Treg, AST, ALT, WAT s100a8, and WAT s100a9.

**Fig. 6:**
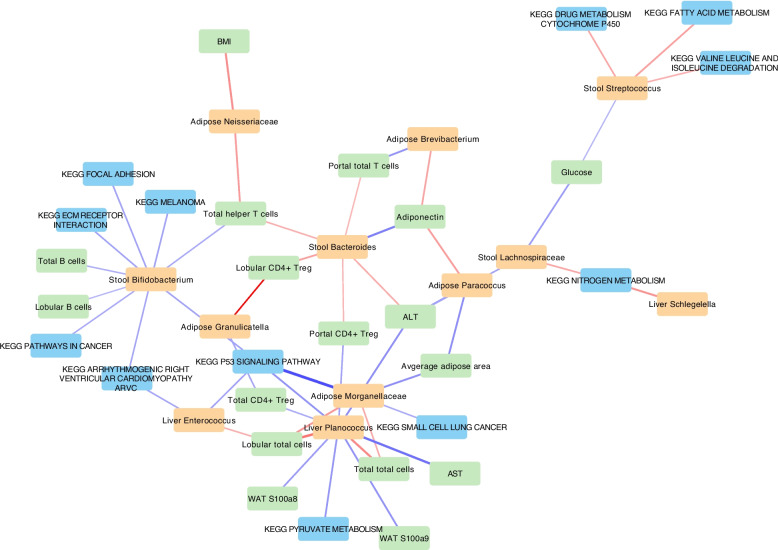
Network analysis of the relationship between significant fecal/adipose/liver bacteria and clinical/immune cell, adipose gene expression, and significant hepatic KEGG pathways.Red is positive and blue is negative correlation. Thicker lines represent higher correlation coefficient. Clinical, immune cell and adipose gene expression is shown in green. Hepatic KEGG pathways are shown in blue, and bacteria are shown in orange. Only significant correlations (*p* < 0.05) are shown

Next, we looked at the correlation between the significant bacteria and DEGs in the liver and found 198 significant correlations (*p* < 0.05) (see Supplementary Material 2). Majority of these correlations belonged to adipose Morganellaceae. However, this did not provide a clear picture of the relationship between the bacteria and hepatic transcriptome. Thus, we also used KEGG pathways which include a network of genes involved in each functional pathway in the liver (Fig. 5B). Stool *Bifidobacterium* also had positive correlations with ECM receptor and focal adhesion as well as several cardiovascular and cancer-related pathways. We also performed a network analysis (Fig. 6). Liver *Planococcus* had the highest number of associations followed by stool *Bifidobacterium.* The strongest association was between adipose Morganellaceae and p53 signalling pathway in the liver.

## Discussion

Our results show a significant shift in the liver and adipose tissue microbiome composition in patients with MASLD and MASH, particularly for patients with severe liver fibrosis. Higher abundance of prevalent stool bacterium including *Streptococcus*, *Bifidobacterium*, and *Bacteroides* in stool and tissues with elevated plasma endotoxins suggests that obesity-induced translocation could contribute to MASLD. Of typical, often commensal, gut taxa, we identified *Enterococcus* and Morganellaceae abundance to be associated with increased disease severity in tissues. We also identified several taxa that are not commonly identified in the gut, such as *Neisseriaceae* and *Brevibacterium*, to be associated with worsening disease severity and fibrosis was enriched in adipose tissue of patients with fibrosis, and negatively correlated with plasma adiponectin. Morganellaceae and *Enterococcus* were enriched in diseased tissues and positively correlated with increased expression of the p53 signalling pathway. These bacterial taxa often include opportunistic pathogen species associated with the advancement of various diseases, as we describe in more detail throughout the discussion. Therefore, there is evidence of bacterial translocation in MASLD, with specific liver and adipose tissue bacteria linked to disease severity, fibrosis, and metabolic dysfunction parameters.

The presence of bacteria in the liver and adipose tissue has only been recently reported in MASLD [9, 11, 12]. These studies are challenging to conduct because of risks of contamination that need to be avoided [30, 31]. In addition to characterizing liver and adipose tissue bacteria following stringent methods to avoid contamination, our study adds to the body of knowledge by assessing for the presence of gut bacterial translocation through quantification of plasma endotoxins and the assessment of tissue-associated microbial communities. We identified certain taxa in common with previous studies [9, 11, 12] and certain taxa that have not previously been characterized in adipose and liver tissue. These differences in disease-associated tissue taxa in our study compared to others may reflect different types of patient population such as the presence of type 2 diabetes, differences in BMI, and using lean control group [9, 11, 12].

### The fecal microbiome is not strongly influenced by MASLD

Regarding fecal microbiome, we did not observe any strong trends of certain significantly enriched or depleted taxa in the stool associated with MASLD, MASH, or fibrosis level in this specific patient population. This could be attributed to the fact that our patient population was homogeneous, being in the same range of obesity and all meeting the NIH criteria for bariatric surgery. This is supported by our previous study in MASLD [16] showing that what drives the difference in fecal microbiome is more the presence or absence of obesity than the grouping according to liver histology. This was also reported by others, when comparing lean to obese subjects [32]. Other factors may also affect the fecal microbiome such as diet; this is relevant since all bariatric patients received the same standardized pre-surgical diet for about 3 weeks. This may have reduced differences in fecal microbiome by the time of surgery. Also, patient demography like age and sex is known to affect the fecal microbiome. Studies in obese subjects with MASLD that do show significant trends in the fecal microbiome for MASLD patients may be reporting data from subject groups with more heterogeneity of diet, age, sex, comorbidities, prognosis, and treatment plan [32–36].

To find consistent trends associated with MASLD, rather than potentially transient changes observed in the gut microbiome, we decided to focus our analyses on the adipose and liver tissue data. Considering that several of these factors may affect the intestinal microbiome and that increased intestinal permeability and bacterial translocation could contribute to MASLD [33, 34], we decided to focus our analyses on the adipose and liver tissue data, the translocation of certain taxa from the gut to liver and adipose tissues, and the relative abundance of potential key players in the tissue microbial communities that may be associated with metabolic dysfunction parameters and elevated disease states.

### Investigation of the tissue microbiome requires rigorous data decontamination

The quantity of bacteria in the colon is estimated to be approximately 10^11^ bacteria per gram of wet content [35]. Our results show on average approximately 10^4^–10^5^ bacteria reside in each gram of liver and adipose tissues, for both NLO and MASLD patients. This is similar to the number of bacteria present in the stomach and small intestines [35]. Overall, adipose tissue contained a lower quantity of estimated bacteria per gram of tissue than the liver, but there was no significant relationship between bacterial quantity and disease state or fibrosis level. The amount of bacteria that would reside in the tissue of non-obese healthy individuals is not well known or studied. The number we observe could be largely inflated due to obesity-induced and inflammation-associated translocation, as is observed in patients with intestinal bowel disease [36]. This quantity is substantially lower than that of the gastrointestinal tract, which inherently leads to difficulties in sample processing and can result in the characterization of bacterial contaminants, rather than bacteria that are truly present within the tissues [31]. This is a significant issue for other environments with low quantities of bacteria, such as blood, and has led to the misinterpretation of data [37, 38]. To circumvent this issue, the processing of extraction and sequencing controls is necessary [39, 40]. Similar to other methods studying tissue microbiomes, we utilized numerous extraction and sequencing negative controls that were processed alongside our samples to estimate, identify, and remove amplicon sequence variances (ASV) that were likely contaminants [9].

Another issue with studying the microbiome of tissues, mucosa, and blood is the large proportion of human DNA relative to bacterial DNA. It is difficult to capture a sufficient amount of bacteria for sequencing and analysis from these human DNA-rich environments, and the presence of human DNA affects the distribution of the bacteria identified [41]. The relative number of human cells may be even higher in samples from inflamed regions, such as liver and adipose tissues from patients with obesity or MASLD, due to the influx of immune cells [42]. To mitigate this issue of host DNA, we utilized a HostZero method, which has been previously shown to significantly reduce the presence of human DNA in tissue samples [43]. The V4 hypervariable region of the 16S rRNA gene was then targeted, following a standard method that is widely used to cost-effectively characterize various microbiomes [44]. This also allowed us to selectively amplify bacterial DNA, rather than utilizing a metagenomic approach, which would have carried forward residual human DNA and reduced our ability to capture the bacterial microbiome accurately. Additionally, we used a method to estimate the absolute abundances of the taxa in the microbiome which enables a more accurate estimation of the microbiome, particularly in samples with low bacterial biomass and a high proportion of host cells [28, 29]. The batch effect was alleviated by modelling the batch variations and correcting the observed abundances of ASVs. Along with standard sterile technique, we believe that these methods of utilizing negative controls, reducing host DNA, selectively amplifying bacterial sequences, analyzing relative and estimated absolute abundances, and correcting for batch effects have allowed us to capture a microbial community from the liver and adipose tissues as accurately as possible. We acknowledge that with only 16S rRNA data, we are not able to track exact strain-level translocation. However, we do believe that the body of evidence we provide, including plasma endotoxin quantification and robust data analysis, it is reasonable to believe that the source of bacteria in the tissues is from the gut.

### Highly prevalent taxa in both feces and tissues suggest a core translocating microbiome

We observed that the most prevalent genera/families in both feces and tissue were *Streptococcus*, *Bifidobacterium*, *Bacteroides*, and Lachnospiraceae. The prevalence of these taxa in both feces and tissue, in NLO and MASLD patients, suggests an obesity-induced translocation of certain core taxa. It is unknown as to why these taxa were so prevalent, but we speculate that either they can translocate more easily from the gut into the tissues, or that they are capable of maintaining colonization more easily in the tissues. Intrahepatic bacteria have been previously observed in MASLD liver tissue [45, 46]. Bacteria have also been identified in different regions of adipose tissue from patients who underwent bariatric surgery [14]. This reinforces the notion that bacterial communities, and not just microbial DNA, exist in the liver tissue and adipose tissue. *Streptococcus* was enriched in the liver of MASLD and MASH subjects, which is supported by the literature [47–49]. Studies have reported that *Streptococcus* is an identifier for liver injury in those experiencing alcoholic liver disease [48] and certain species correlated with abundance of toxic metabolite in MASLD [49]. In addition, *Streptococcus* infection in murine models led to infiltration of the liver by inflammatory cells [47]. We also found that *Bifidobacterium* was increased in those with MASLD, which conflicts with what is known in the literature. *Bifidobacterium* is a commonly tested probiotic for the treatment of MASLD, known to modulate host’s metabolism and reduce pro-inflammatory markers in a metabolic syndrome population [50–53]. Different species and strains of *Bifidobacterium* have shown to also mitigate MASLD by altering hepatic fat deposition and inflammation, lipid and glucose metabolism, and intestinal permeability [45, 54, 55]. Stool *Bifidobacterium* correlated positively with B cells and helper T cells. Species from *Bifidobacterium* can communicate with the immune system and modulate secretion of cytokines and differentiation of naïve T cells to specific helper T cells (reviewed in [53]). Surprisingly, *Bifidobacterium* correlated positively with “ECM receptor interaction” and “focal adhesion” pathways as well as cardiovascular and cancer-related pathways; however, the relationship is not fully understood. In our study, the presence and increased relative abundance of *Bifidobacterium* in liver MASLD and the observed relationship with cancer-related pathways may be due to increased general translocation from the gut during an advanced disease state, rather than a contributor to the disease itself.

*Bacteroides* is the greatest contributing taxon for the overall microbiome structure in tissues, and therefore, the presence of this taxon in the liver and adipose tissues affects the rest of the tissue microbial community. Intestinal *Bacteroides* abundance is often associated with MASLD disease progression [46, 56]. We observed that stool *Bacteroides* positively correlated with adiponectin and negatively with Treg and helper T cells. Occasionally, the abundance of certain *Bacteroides* species, such as *B. xylanisolvens*, *B. acidifaciens*, or *B. dorei*, has strengthened intestinal integrity and reduced inflammation and lipid hepatic accumulation and steatosis [57–60]. However, other species, such as *Bacteroides caccae*, can degrade mucin layer of the gut, leading to diminished intestinal barrier integrity and increased translocation of bacteria [61]. The importance of *Bacteroides* on overall community structure of the tissue microbiome may be due to a species-specific effect on intestinal permeability through T cell regulated inflammation.

### Adiponectin regulation in MASLD correlated with *Brevibacterium* and *Paracoccus* abundance

*Brevibacterium* is a common skin bacterium [62], which was significantly enriched in the presence of fibrosis. In a previous study, rats with induced MASH showed higher levels of *Brevibacterium* in the gut, which was significantly reduced following treatment with Metformin and Berberine [63]. *Brevibacterium* negatively correlated with adiponectin. Adiponectin has been demonstrated to have an anti-fibrotic effect in the liver by blocking the activation of certain hepatic pathways and reducing the expression of pro-fibrotic genes [64]. *Paracoccus* was also negatively correlated with adiponectin and positively with ALT and average adipose area. One of the species of *Paracoccus* has been reported as an opportunistic pathogen in patients with decompensated cirrhosis [65]; however, not much is known with regard to *Paracoccus*’s role in health and disease, especially in the context of liver disease. As we do not know the directionality of the effect, we speculate that the increased abundance of *Brevibacterium* and *paracoccus*, in adipose tissue, reduces the secretion of adiponectin, leading to MASH and/or increased levels of fibrosis in the liver, or the pro-fibrotic environment due to low adiponectin allows for the growth of these bacteria.

### Disease progression to MASH may be related to the abundance of key taxa: investigating *Enterococcus*, *Planococcus*, *Granulicatella*, and Morganellaceae

In the liver, *Enterococcus*, *Planococcus*, *Granulicatella*, and *Schlegelella* were all identified as significantly enriched in MASH compared to NLO. Though identified as significant, these taxa were not found to be ubiquitously enriched in MASH. MASH samples, in particular liver, showed a significantly higher degree of heterogeneity between samples, based on beta diversity distances. This observation aligns with our relative abundance analysis, as only certain samples show enrichment of certain taxa. We hypothesize that these taxa may be opportunistically pathogenic in the MASH environment and establish themselves in a patient-specific manner.

*Enterococcus* is a normal gut bacterium that can be translocated from the intestines and persist in the body by altering the state of phagosomes [66]. The use of proton pump inhibitor medications which contribute to elevated translocation of *Enterococcus* to the liver has been associated with increased risk of MASLD and MASH [67]. Certain species of *Enterococcus* are also capable of creating hemolytic bacterial toxins and hyaluronidase, an enzyme that can degrade tissues by breaking apart hyaluronic acid (HA) [66]. In MASH, a higher quantity of HA within the blood serum is an indicator of more advanced fibrosis [68]. Certain *Enterococci* species are capable of secreting cytolysin and causing hepatocyte death and liver injury, and carcinogenesis in other liver diseases [69, 70]. We hypothesize that the increased estimated abundance of *Enterococcus* in MASH patients relative to NLO is potentially contributing to the progression of disease, possibly due to the presence of certain enzymes capable of tissue degradation.

*Planococcus* is generally characterized as an environmental bacterium, associated with various soil and water ecosystems [71]. Recently, certain species of *Planococcus* have been identified in human blood [72] and, separately, characterized to include the genetic material capable of producing multidrug resistance, suggesting the possibility of an emergent opportunistic pathogen [71]. The role of *Planococcus* is unknown in MASLD development, but the significant enrichment in MASH liver tissue, and the significant correlation with multiple markers associated with liver damage (AST, ALT, WAT s100a8 and s100a9), suggests a potentially interesting and important role of this bacterium.

Morganellaceae and *Granulicatella* were significantly enriched in adipose MASH, and in the liver were significantly correlated with the relative abundance of *Enterococcus*. *Granulicatella adiacens* has been previously associated with severe diseases such as MASH, fibrosis, and cirrhosis [50]. Another study in patients with MASLD showed a positive association between HOMA-IR and *Granulicatella* [72]. When adjusted for HbA1c, we found higher levels of *Granulicatella* in MASH adipose tissue which correlated positively with Treg. This suggests *Granulicatella* might play a role in MASH development independent of glycemic control. Morganellaceae and *Enterococcus* were also both strongly positively correlated with increased expression of the p53 signalling pathway. Increasing evidence links obesity to the onset of cancer and the role of p53 in tumor development and liver fibrosis (as reviewed in [73]). *Proteus mirabilis* (from the family Morganellaceae) and *Enterococcus faecalis* can be found as persistent co-colonizers in vivo, capable of forming a biofilm directly on the intestinal epithelium, disseminating to the bloodstream and mesenteric lymph nodes [74–77]. Interestingly, a previous study showed Morganellaceae to be present in the liver tissues of obese rats and completely absent from non-obese rats [78]. The presence and correlated abundance of Morganellaceae and *Enterococcus* in our study could be an observation of symbiosis between these taxa, potentially allowing for the development of biofilms within the liver and adipose tissues of patients with MASH. This persistent colonization of cytolytic-capable bacteria, such as *Enterococcus*, within the tissues could thus play a role in p53-induced liver fibrosis.

To our knowledge, this is the first study that links the tissue microbiome to metabolic dysfunction parameters that include hepatic transcriptome pathways in humans. The strengths of this study are the relatively large sample size, use of adipose, liver, and stool as well as the use of liver biopsy for MASLD diagnosis and determining its severity. We also utilized a methodology that allowed for both relative and estimated absolute abundance quantification, which provides a more accurate analysis of the microbiome. We also put in place measures to prevent environmental and processing contamination. We included several sets of controls at each tissue and sequencing manipulation step, for DNA extraction kit and reagent contaminants, and 16S rRNA gene amplification and sequencing controls, followed by rigorous statistical testing to reduce risk of reporting false-positive results. The limitations of this study include the cross-sectional aspect and lack of complete data regarding species.

## Conclusion

Our work demonstrates an intricate relationship between MASLD severity and various microbiological, immunological, and gene expression factors. MASLD severity can be characterized not by a general translocation of gut bacteria to hepatic and adipose tissue, but by specific microbial patterns within these diseased tissues, particularly in those with severe liver fibrosis and/or MASH. We identified higher abundances of specific taxa in these diseased tissues relative to normal livers from obese patients (NLO). Coupled with elevated plasma endotoxin levels, we provide evidence supporting the idea that obesity-induced bacterial translocation is linked to the progression of MASLD.

This research uniquely investigates the relationship between the tissue microbiomes and metabolic dysfunction parameters, including hepatic transcriptome pathways, in humans. Our methodology, including relative and absolute abundance quantification and contamination controls, a relatively large sample size, and the use of multiple tissue types, provides a robust analysis of the tissue microbiome. The role of these taxa remains primarily speculative, as it is unknown whether certain taxa cause the progression of MASLD, or if the MASLD environment promotes the colonization and proliferation of certain taxa. Likely, there is an ongoing synergistic effect of these two factors at play that influences inflammatory genes and immune cell responses, contributing to the progression of the disease and the development of severe fibrosis and MASH. This underscores the importance for a deeper understanding of the microbiome’s role in MASLD, to identify potential therapeutic targets to improve disease management strategies for this complex disease.

## Supplementary Information

Supplementary Material 1.

Supplementary Material 2.

## Data Availability

The data that support the findings of this study are not openly available due to ongoing publications and are available from the corresponding author upon reasonable request. Once the related manuscripts are published, data will be deposited into a data repository.
