# Supplementary material for: Characterization of liver, adipose, and fecal microbiome in obese patients with MASLD: links with disease severity and metabolic dysfunction parameters
Source: Microbiome. 2025 Jan 14;13:9. doi: 10.1186/s40168-024-02004-7 (PMC11730849; doi:10.1186/s40168-024-02004-7)
Supplement: Supplementary file 2 — Supplementary Material 1. [file 40168_2024_2004_MOESM1_ESM.docx]

**Supplementary**

**Materials and Methods:**

**Study Design**

As cited in previously published articles ^1^, the inclusion criteria were fulfilling the NIH criteria ^2^ and suitable candidates for bariatric surgery; male/female; age > 18 years; alcohol intake <20g/d; on stable drug regimen > 3 months for hyperlipidemia or type 2 diabetes prior to study entry. Exclusion criteria included: Liver disease other than MASLD; medications known to precipitate MASH 6 months prior to entry; regular intake of non-steroidal anti-inflammatory drugs; prebiotics, probiotics or antibiotics or any experimental drug in the 3 months prior to study entry; type 1 diabetes, chronic gastrointestinal diseases, previous gastrointestinal surgery modifying the anatomy (prior to bariatric surgery), smoking; pregnancy or breastfeeding.

**Clinical and biochemical measurements**

Blood work was collected in the morning after a 12-hours fast. The certified University Hospital Laboratory Medicine Program analyzed samples for HbA1c, fasting insulin, glucose, liver enzymes, lipid profile, albumin and platelets using standard protocols see **Supplementary Methods**. The homeostasis model for insulin resistance (HOMA-IR) was used [glucose (mmol/L) X insulin (mU/L / 22.5)] as an indirect measure of insulin resistance^3^. Plasma adipokine, cytokine, gut hormones, fibroblast growth factor (FGF)-23, FGF-19 and endotoxin were also measured (see details are in Supplementary Material). Weight, height, body mass index (BMI), waist circumference, and systolic and diastolic blood pressure were also collected by a registered nurse.

**Liver Biopsy**

Immune Cell: multiplex immunofluorescence (MIF) staining was conducted at BC Cancer (British Columbia, Canada). Briefly, a 4-μm-thick section was cut from formalin-fixed paraffin-embedded liver tissues. Slides were stained with an 8 colour + DAPI multicolour immunofluorescent panel (CD16a, CD79a, CD3, CD94, CD68, FoxP3, CD8, panCK) in batches of up to 20 slides. Each batch of slides were incubated overnight at 37C then deparaffinised through xylene and graded reagent alcohols (Fisher Scientific). Antigen retrieval was performed at 110C for 15 minutes in a Biocare decloaking chamber using nuclear decloaking solution (Biocare) before loading the slides onto a Biocare Intellipath FLX Autostainer. The multicolour IF process involved polymers from Biocare and OPAL fluors from Akoya. The first round of staining involved performing endogenous peroxidise blocking with Peroxidased-1 (Biocare) followed by non-specific blocking with Background Sniper (Biocare). The primary antibodies CD16a (Clone SP175 Abcam), CD79a (clone SP18, Abcam), CD3 (Clone PS1, Biocare), CD94 (clone EPR21003, Abcam), CD68 (clone SP251, Abcam), FoxP3 (clone 236A/E7, Fisher Scientific), CD8 (C8/144B, Cell Marque), PanCK+ (clone AE1/AE3 + 5D3, Biocare) were added and incubated subsequently. Following the antibody rounds the slides were counterstained with DAPI (Akoya) and coverslipped with Prolong Diamond Antifade mounting media (Fisher Scientific). Slides were imaged on the Vectra Polaris multispectral imaging system (Akoya), a low power scan was completed then regions of interest selected for multispectral image collection. Images were analyzed using inForm image analysis software (Akoya). Briefly, a base algorithm comprised of tissue and cell segmentation was constructed followed by individual single phenotype algorithms for each of the markers. The algorithms were trained on a smaller set of images then applied to all images in a batch run. Discovery of the immune subtypes was conducted using output from inForm which was overlaid in Phenoptr (Akoya) to define phenotypes and obtain counts. Phenotypes are defined as: CD79a+ total B cells; CD3+ total T cells; FOXP3-/CD8+/CD3+ killer T cells; FOXP3-/CD8-/CD3+ helper T cells, FOXP3+/CD8-/CD3+ CD4+ T reg; FOXP3+/CD8+ CD8+ T reg; CD94+/CD3- NK cells; CD94+/CD3+ possible NKT; CD16a+/CD68- possible toxic NKs; CD16a-/CD68+ macrophages; CD16a+/CD68+ activated macrophages. Cell density (number of cells/mm^2^) of each immune cell subtype is calculated at 20 portal areas and 20 lobular areas for each slide. The final cell density of each immune cell subtypes in portal/lobular area was calculated as mean ± SD using 20 areas.

DNA Extraction for Tissue Microbiome: Samples were randomized, and DNA was extracted from adipose and liver tissues using the ZymoBIOMICS Host-ZERO microbial DNA prep kit (D4310), following a modified version of the Solid Tissues method in Appendix B. The ZymoBIOIMCS Spike-In Control II (Low Microbial Load, D6321) was added to the extraction. To summarize, liver and adipose tissues were weighed and <125 mg of tissue was added to a MN Bead Tubes Type E (Macherey-Nagel) with 900 uL of DNA/RNA shield (ZymoBIOMICS). If the weight of the tissue sample was <50mg, 5 uL of Spike-In was added, if the weight of the tissue was >50mg, 10 uL of Spike-In was added. This volume was established based on a trial sample subset (data not shown), which indicated that with a greater sample volume, a greater amount of Spike-In Control II would be required in order to achieve the rough target of 20% Spike-In sequences in the final sample library. The Bead Tubes were then bashed in the Bead Beater for 3 minutes and centrifuged at 10,000xg for 1 minute. 200 uL of supernatant was transferred to the Host-Zero DNA depletion step 1, and the remaining extraction protocol was followed as described in the ZymoBIOMICS Host-Zero kit manual. Negative (no input) controls were processed alongside each batch of samples (10 negative controls total), a negative control with 10 uL of Spike-in, and positive control, the ZymoBIOMICS Microbial Community Standard (D6300) with 10 uL of Spike-In, were processed following the same extraction method as described.

Tissue Microbiome Sequencing: The V4 hypervariable region of the 16S rRNA gene was amplified using uniquely barcoded 515F (forward) and 806R (reverse) sequencing primers to allow for multiplexing^4^. Amplification reactions were performed using 12.5 uL of KAPA2G Robust HotStart ReadyMix (KAPA Biosystems), 1.5 uL of 10 uM forward and reverse primers, 7.5 uL of sterile water and 2 uL of DNA. The V4 region was amplified by cycling the reaction at 95**°**C for 3 minutes, 30x cycles of 95**°**C for 15 seconds, 50**°**C for 15 seconds and 72**°**C for 15 seconds, followed by a 5-minute 72**°**C extension. All amplification reactions were done in triplicate to reduce amplification bias, pooled, and checked on a 1% agarose TBE gel. Pooled triplicates were quantified using PicoGreen and combined by even concentrations. The library was then purified using Ampure XP beads and loaded on to the Illumina MiSeq for sequencing, according to manufacturer instructions (Illumina, San Diego, CA). Sequencing is performed using the V2 (150bp x 2) chemistry. A single-species (*Pseudomonas aeruginosa* DNA), a mock community (Zymo Microbial Community DNA Standard D6305), and triplicate template-free negative control were included per sequencing run. Two sequencing runs were performed in order to achieve adequate sequencing depth per sample, given the number of total samples.

RNA Extraction for Transcriptome: RNA extraction by Trizol method was performed by Novogene Inc. (USA). After cell lysis, impurities removal, and inhibition of RNAse activity, total RNA was extracted by using phase separation method from cell debris. Then, Agilent bioanalyzer 2100 were used to check RNA integrity and concentration. For samples with RIN ≥4, library of mRNAs was prepared and sequenced using Illumina platform and paired-end sequencing. Reads with adaptor, poly-N reads as well as low-quality reads were removed to obtain clean raw data which were then mapped to reference genome using Hisat2 version 2.0.5. and read count was obtained using featureCounts version 1.5.0-p3.

**Adipose Biopsy**

RNA Extraction for Adipose Gene Expression: As previously published^5^, *RNA extraction and cDNA synthesis:* Total RNA was extracted from patient’s VAT using Trizol reagent combined with GeneJET RNA Purification Kit (Thermofisher Scientific Cat number #K0732). Total RNA concentration and purity was determined by nanodrop (Thermo Scientific™ NDONECWQUBIT4). From 1 µg of RNA as a template, cDNA was synthesized using Verso cDNA Synthesis Kit (Thermofisher Scientific Cat number #AB1453A) as per manufacturers protocol. The cDNA samples were stored at -20°C until further analysis. *Quantitative polymerase chain reaction (qPCR):* qPCR analyses were performed in 384-well plates on a CFX384 Touch Real-time PCR machine (Bio-rad) in a final volume of 10 μL, using PowerUp SYBR® Green Mastermix (Applied Biosystems) and primer sets targeting several human genes related to inflammation, proper functioning, browning and fibrosis of adipose tissue (LEP (leptin gene), RETN (resistin), TNF, IL6, ADIPOQ (adiponectin), UCP1, PPARG, PPARA, SLC2A1 (GLUT1), PRDM16, CIDEA, PPARGC1A, RBP4, calprotectin/S100A8 and 9, MCP-1/CCL2, CCL4, CCR2, IFNG, IL1B, NOS2, CLEC10A, IL10, CYBB (NOX2), TGFB1, VEGF, COL1A1, COL3A1, COL6A1, LOX, LOXL2, CD9, ACTA2, MMP2, MMP9, FN1, CTGF, E2F1, DPP4). Controls were RPLP0, TBP, LRP10. All qPCR reactions were run in triplicate. The thermal cycling profile included initial denaturation at 95 °C for 10 min, followed by 40 cycles of 15 sec at 95°C and 1 min at 58°C. A melting curve was generated for each set of primers to discriminate between specific and non-specific amplification products. The delta CT (∆Ct = ct values of gene - mean ct value of housekeeping control genes) values were used to calculate the relative expression of different target genes. A higher ∆Ct value is indicative of lower RNA levels (more PCR cycles required to exceed background).

DNA Extraction for Tissue Microbiome: See section above under Liver Biopsy sub-section DNA Extraction for Tissue Microbiome.

**Fecal Microbiome**

Stool collection and DNA extraction: As previously published^1^, DNA was extracted, according to the manufacturer’s protocol, with some modifications, using ZymoBIOMICS DNA Miniprep Kit (ZYMO RESEARCH, Irvine, CA, USA). Briefly, 100-200mg of fecal sample was mixed with 750µL of the lysis solution in a 2mL beaded tube and homogenized using dry ice and Precellys Evolution (Bertin Technologies S.A.S, France) using 5 cycles of 8000rpm with two minutes between cycles. The tube was centrifuged at 10000g for 1 minute and the supernatant was transferred to a 2mL tube and re-centrifuged at 3000g for 1 minute. DNA was eluted for 5 minutes, and the remaining extraction procedure was performed using the manufacturer’s protocol.

Fecal Microbiome Sequencing: The V4 hypervariable region of the 16S rRNA gene was amplified using uniquely barcoded 515F (forward) and 806R (reverse) sequencing primers to allow for multiplexing^4^. Amplification reactions were performed using 12.5 uL of KAPA2G Robust HotStart ReadyMix (KAPA Biosystems), 1.5 uL of 10 uM forward and reverse primers, 7.5 uL of sterile water and 2 uL of DNA. The V4 region was amplified by cycling the reaction at 95**°**C for 3 minutes, 18x cycles of 95**°**C for 15 seconds, 50**°**C for 15 seconds and 72**°**C for 15 seconds, followed by a 5-minute 72**°**C extension. All amplification reactions were done in duplicate to reduce amplification bias, pooled, and checked on a 1% agarose TBE gel. Pooled duplicates were quantified using PicoGreen and combined by even concentrations. The library was then purified using Ampure XP beads and loaded on to the Illumina MiSeq for sequencing, according to manufacturer instructions (Illumina, San Diego, CA). Sequencing is performed using the V2 (150bp x 2) chemistry. A single-species (*Pseudomonas aeruginosa* DNA), a mock community (Zymo Microbial Community DNA Standard D6305), and a template-free negative control were included.

**Bioinformatic Analysis of Tissue and Fecal Microbiome**

The Qiime2 analysis package version 2023.2 was used for sequence analysis, the following functions were accessed from within the Qiime2 package^6^. The quality of the sequencing runs were first examined using FastQC and MultiQC ^7,8^. Cut adapt was used, following the default settings, to remove sequences with high errors rates^9^. Paired-end sequences were assembled, and quality trimmed using vsearch –fastq_mergepairs ^10,11^, following default settings, with a –fastq_truncqual set at 2, a maxee set at 1, and minimum and maximum assemble lengths set at 250 and 255 (+2 and -3 base pairs from the expected sequence length of 253bp). Assembled sequences were subjected to an additional filtering step, utilizing the quality-filter function in Qiime2. The resulting high-quality data was then processed following the deblur pipeline. Sequences were clustered into Amplicon Sequence Variant (ASV) groups and singleton sequences were removed. Taxonomy assignment was executed using the Qiime2 classify-hybrid-vsearch-sklearn function and the Average ReadyToWear trained Silva database version 138.1^12,13^. ASVs with an abundance less than 0.01% were removed to reduce the potential for observing bleed-through ASVs, and ASVs identified as contaminating chloroplast or mitochondria are removed. A phylogenetic tree is created using the SEPP function available through Qiime2^14^.

The data was then analyzed using R version 4.3.0. First, the ASV table containing all sample types (fecal, adipose, liver) was filtered to include ASVs with a prevalence greater than 3 and a total and average abundance greater than the 75^th^ percentile, as the data skewed highly toward zero count and low prevalence and abundance ASVs. Extraction and sequencing control samples were analyzed, and the 10 negative extraction controls were utilized by the R package ‘Decontam’ verison 1.20.0 in order to identify contaminant ASVs. The sample quantification determined with PicoGreen (see Tissue and Fecal Microbiome Sequencing sections) was used as the “conc” parameter and the method was set to “either”, with a prevalence threshold set at 0.1 (default). Additionally, ASVs with a total abundance >100 in the negative extraction controls or >10 in the negative PCR controls, were also selected to be addressed as contaminants.

The data was then corrected to reduce batch effects using the R package ‘ConQuR’ version 2.0 following the default parameters. Spike-In taxa were removed from the tissue data.

The tissue-only data was then analyzed separately. ASVs identified in only stool samples were first removed from the raw data. Again, the data was then filtered to include ASVs with a prevalence >3 and a total and average abundance >75^th^ percentile. The R pacakge ‘Decontam’ verison 1.20.0 was used to identify contaminants and the R package ‘ConQuR’ version 2.0 was used to correct for batch effects, and the spike-in taxa were removed.

The tissue data was then also re-scaled utilizing the Spike-In data (stool samples did not contain a spike-in, again because they were sequenced prior to the utilization of this control). The relative abundance of the Zymo Spike-In taxa *Imtechella* was determined and the Total Cells per gram of tissue weight were calculated following the protocol described in the Zymo Spike-In Control I manual. The ASV table was then scaled relative to the Total Cells identified in each sample, creating an ASV table with estimated ASV absolute abundances. Samples that did not have any Spike-In control identified in the sample data, most often due to very low sequencing depth, were removed from the analysis.

**Plasma Adipokine, Cytokine, Gut hormones, FGF-23, FGF-19 and Endotoxin Measures**

As previously published^5^, using Luminex ELISA cytokines and adipokines were quantified from the plasma samples using a custom Luminex assay (Bio-Rad) according to manufacturer’s instructions. The panel of cytokines and adipokines included were: 6 Plex (IL-6, IL-10, TNF-α, CCL-2, Leptin, Resistin), 2 Plex (Adiponectin, Retinol binding protein-4), one-Plex (C-reactive protein). After collection, blood samples were centrifuged at 14,000 rpm for 10 min and the plasma samples were stored at -80°C. Plasma samples were diluted 2 times, 500 times and 2000 times for 6 plex, 2 plex and 1 plex respectively. Samples were analysed on the same plate at the same run-in random order, and sample status was blinded to those performing the biochemical analysis. Plates were analysed with a Bio-Plex MAGPIX Multiplex Reader (Bio-Rad). The concentrations of different analytes were calculated by standard curve.

As previously published^15^, GLP-1, PYY, ghrelin, leptin and FGF-23 were measured using 50µL of plasma and U-PLEX Obesity Combo 2 (Mesoscale Discovery, Gaithersburg, MD 20877 USA). Manufacturer’s protocol was followed and the results were read with Meso Scale Discovery Sector 2400A (Mesoscale Discovery, Gaithersburg, MD 20877 USA). In addition, we measured FGF-19 in 100µL of diluted serum (1:2) using FGF-19 Quantikine ELISA kit (R&D Systems, Minneapolis, MN, US) according to manufacturer’s protocol^16^. Plasma endotoxin was measured using Limulus Amebocyte Lysate PYROGENT™-5000 kits (Lonza, Walkersville, MD, USA) with some modifications.

**Statistical Analysis**

Hepatic transcriptome analysis: Raw counts were normalized using fragments per kilobase of transcript per million mapped reads (FPKM) and principal component analysis (PCA) was performed to assess the intergroup variability. Differential gene expression (DEG) was analyzed using DESeq2 version 1.38.3. A table of results was created for each comparison. *P* value was adjusted during Wald testing using Benjamini-Hochberg to account for multiple hypothesis testing and log2FoldChange was calculated without shrinkage. DEG were identified using p-adjusted<0.05 and |log2FC|≥1. Functional enrichment analysis was performed using ClusterProfiler version 4.6.2. DEG with raw *P* <0.05 was used for gene ontology (GO) and Kyoto Encyclopedia of Genes and Genomes (KEGG), DEG with raw p-value<0.05. A gene set enrichment analysis (GSEA) of GO and KEGG was performed using gseGO and gseKEGG, respectively, using DEG with raw *P* <0.05. All data are analyzed using R version 4.3.2. Gene set variation analysis (GSVA) was performed using GSVA package version 1.50.0. with KEGG database as the reference gene set.

Tissue and fecal microbiome analysis: The data was analyzed using R version 4.3.0. Phyloseq version 1.44.0 was used for data manipulation and analysis. Ggplot2 3.4.3. was used for data visualizations. Chao1 alpha diversity was calculated using the ‘estimate_richness’ function and was compared between sample groups using the Anova lm function and a TukeyHSD for post-hoc comparisons (p adjusted < 0.05). MetagMisc function ‘phyloseq_transform_css’ was used to convert the ASV table to cumuliative sum scores (CSS) for the for beta diversity analysis, following the default parameters. Vegan 2.6.4 was used to calculate Bray-Curtis distances and determine diversity using the ‘adonis2’ function. The method “terms” was used by the adonis2 function, inputting the factors in the order of sample type, HbA1c, factor of interest, age, sex, and subject ID. This was performed in order to identify the role of the factor of interest after accounting for the potential effect of HbA1c. The *P* value correction for multiple comparisons was set to BH p-adjusted value *P* < 0.05 (default). The ‘plot_ordination’ function of Phyloseq was used to create the PCoA plots. MicroViz 0.10.8 was used to create the PCA plots with CLR transformed data with Aitchison PERMANOVA distance analysis. Mia version 1.8.0 function ‘runDMN’ was used to determine community clusters with laplace correction. MaAsLin2 version 1.15.1 was used for differential abundance analysis of genera, following default parameters (Briefly: CLR normalization, LM analysis method, and a min prevalence of 0.1 for unscaled relative abundance data and 0.2 for scaled estimated abundance data). Fixed effects were set as the factor of interest (disease state or fibrosis level, specific to the particular analysis), age, sex, and HbA1c value. The p-value correction for multiple comparisons was set to BH p-adjusted value *P* < 0.1 (default). Taxa identified as significantly associated with the factor of interest had a significant adjusted p-value after comparing the differential abundance of the scaled estimated absolute abundances. To be carried forward in the analysis, these taxa were supported by either a significant association of the unscaled relative abundance data, or by a positive log-fold change (LFC) value associated with the factor or interest and the absence of a positive LFC associated with HbA1c value. Heatmap of correlations between significant bacteria and variables of interest was created using Spearman correlation coefficient (with unadjusted *P* value) and rstatix package version 0.7.2.

**Results:**

**Microbiome Data Processing Results: Filtering, Decontamination, Batch Correction:**

The ASV table (see Supplementary Methods **Bioinformatic Analysis of Tissue and Fecal Microbiome**) containing all sample types (fecal, adipose, liver) was filtered to include ASVs with a prevalence greater than 3 and a total and average abundance greater than the 75^th^ percentile, as the data skewed highly toward zero count and low prevalence and abundance ASVs. This reduced the number of ASVs from 38906 to 1734, while only reducing the average total number of reads per sample from 24777 to 23855.

Extraction and sequencing control samples were analyzed, and the 10 negative extraction controls were utilized by the R package ‘Decontam’ verison 1.20.0 in order to identify contaminant ASVs. The negative extraction controls primarily contained the following genera: *Acinetobacter, Alcaligenes, Brevundimonas, Comamonas, Delfia, Pseudomonas, Shinella*, and *Staphylococcus*. Aside from *Staphylococcus*, which is a type of Bacilli, these genera are all Proteobacteria, primiarly Gammaproteobacteria and Betaproteobacteria. The sample quantification determined with PicoGreen (see Supplementary Methods **Tissue and Fecal Microbiome Sequencing**) was used as the “conc” parameter and the method was set to “either”, with a prevalence threshold set at 0.1 (default). This resulted in 223 ASVs being identified as contaminants. Additionally, ASVs with a total abundance >100 in the negative extraction controls or >10 in the negative PCR controls, were also selected to be addressed as contaminants. This resulted in a total of 12 additional ASVs identified as contaminants. The ASVs that were eliminated from the data included all the high abundance taxa present in the negative controls, as described above, as well as other lower abundance ASVs that were identified as contaminants by the software.

The data was then corrected to reduce batch effects using the R package ‘ConQuR’ version 2.0 following the default parameters, setting the covariates as: sample type (stool, adipose, liver), disease state, and fibrosis level. This method attempted to correct the relative abundances of ASVs to reduce the effects of PCR and sequencing batches and resulted in the additional removal of 7 ASVs. Spike-In taxa were removed from the tissue data, and the result was a total of 1493 ASVs to be analyzed.

The tissue data was then analyzed separately. ASVs identified only in stool samples were first removed from the raw data, resulting in 34377 ASVs present in the tissue-only ASV table. Again, the data was then filtered to include ASVs with a prevalence >3 and a total and average abundance >75^th^ percentile. This resulted in a total of 1210 ASVs to be analyzed. Again, the R pacakge ‘Decontam’ verison 1.20.0 was used to identify contaminants, the R package ‘ConQuR’ version 2.0 was used to correct for batch effects, and the spike-in taxa were removed, following the same methods described above. This resulted in a total of 1049 ASVs to be analyzed in the tissue-only data table. The tissue-only data was decontaminated and batch-corrected separately from the stool data following this method because the software was able to perform more robustly when the stool data was not included, as the stool data was sequenced previously and was therefore self-contained in a separate batch, meaning that the stool sample type and stool sample batch could not be discerned or properly corrected for. The tissue data was processed randomly and distributed over multiple batches, allowing for batch correction to be more effective for the tissue-only data.

The tissue data was then also re-scaled utilizing the Spike-In data (stool samples did not contain a spike-in, again, because they were sequenced prior to the implementation of this control). The relative abundance of the Zymo Spike-In taxa *Imtechella* was determined and the Total Cells per gram of tissue weight were calculated following the protocol described in the Zymo Spike-In Control I manual. The other taxa present in the Zymo Spike-In Control II, *Truepera* and *Allobacillus,* were not identified at the expected relative abundance compared to the relative abundance of *Imtechella*. We speculate that for Truepera, this was due to either an inability to lyse the cells properly during extraction or a very high GC content, which inhibited PCR and sequencing, as is described in the Zymo Spike-In Control II manual. We speculate that for *Allobacillus*, this may have been because the input quantity was too low for accurate detection. The ASV table was then scaled relative to the Total Cells identified in each sample, creating an ASV table containing the estimate absolute abundances of each ASV per sample. Samples that did not have any Spike-In control identified in the sample data were removed from the analysis. This eliminated an additional 4 samples from the estimated absolute abundance ASV table during the tissue-only analysis.

**Supplementary Tables:**

**Table S1: Summary of measured adipokines, cytokines, gut hormones and hepatic immune cells function and relationship to MASLD/MASH**

| **Parameter** | **Function** | **Relation with MASLD/MASH** |
| --- | --- | --- |
| Interleukin 6 | Pro-inflammatory cytokine | Lead to insulin resistance and fat deposition in the liver. Stimulate stellate cells promoting liver fibrosis. ^17,18^ |
| Interleukin 10 | Anti-inflammatory cytokine | Protect against hepatic steatosis and fibrosis. ^17,19,20^ |
| Tumour Necrosis Factor alpha | Pro-inflammatory cytokine | Lead to insulin resistance, dysfunction in lipid metabolism, release of inflammatory cytokines, and fibrosis in the liver. Associated with the development of MAFLD and progression to MASH. ^17,18^ |
| Chemokine ligand 2 | Inflammatory chemokine | Increased in MASLD and linked to monocyte recruitment to the liver. ^21,22^ |
| Leptin | Dual action of anti-steatotic and pro-inflammatory/pro-fibrotic | Anti-steatotic in early MASLD and promote inflammation and fibrosis in later stages. ^23^ |
| Resistin | Pro-inflammatory | Linked to insulin resistance, inflammation, steatosis and fibrosis. ^24^ |
| Adiponectin | Anti-inflammatory; inhibit migration/proliferation of activated stellate cells in the liver | Decrease insulin resistance, and attenuates liver inflammation and fibrosis. ^25^ |
| Retinol binding protein 4 | Vitamin A transporter; linked to metabolic diseases | Increased in MASLD. But the mechanism remains unclear. ^26^ |
| C-reactive protein | Inflammatory marker | Increased in MASLD. A prognostic predictor of hepatic damage progression in MASLD ^17^. |
| Glucagon-like peptide 1 | Reduction of appetite; insulinotropic | Treatment with GLP-1 receptor agonist ameliorated MASLD by regulating lipid metabolism. ^27^ |
| Protein YY | Reduction of appetite | Potentially linked to amelioration of MASLD. ^27^ |
| Ghrelin | Increase appetite | Increased in MASLD. ^27^ |
| Fibroblast Growth Factor 23 | Adipose tissue regulation | Increased in MASLD and associated with increased fat in the liver. ^28^ |
| Fibroblast Growth Factor 19 | Increase lipid metabolism; regulating protein and glucose metabolism | Generally, reduced in MASLD but some studies showed no impact. It reduces fat deposition in the liver. ^29^ |
| Endotoxin | Bacterial cell wall | Increased endotoxin in those with MASLD/MASH^30^ |
| Killer T cells | Cytotoxic role (produce IFNγ, TNF and cytotoxic molecules) | Elevated in MASH ^31,32^ |
| Helper T cells | Regulate pro and anti-inflammatory immune processes | Depending on the specific sub-type of helper T cell it has been found be increased or decreased in MAFLD/MASH^32^ |
| CD4+ T regulatory cells | Cytotoxic role | Increased in individuals with MASH ^32,33^ |
| CD8+ T regulatory cells | Produce inflammatory cells, such as TNF | Dominate the portal tracts in NASH patients ^34^ and drive adipose tissue inflammation, enhancing the recruitment of inflammatory monocytes ^32,35,36^. |
| Natural killer cells | Cytotoxic role | Elevated in MASH ^31,32^ |
| Natural killer T cells | Promote the activation of both inflammatory T helper 1 cells and anti-inflammatory T helper 2 cells ^37^ | Depleted in steatosis but increase as the disease progresses, contributing to inflammation and fibrosis ^32,38^. |
| Macrophages | Immunoregulatory and detoxifying functions | Increased in MAFLD/MASH ^33^ |
| B cells | Influence immune-mediated inflammatory responses | Increased activation in MASH ^32,39^ |

**Table S2: Comparison of anthropometric and biochemical data between Normal Liver Obese (NLO) vs MASLD** **subjects and NLO versus MASH subjects**

| **Variable** | **NLO (n=26)**  **Median [IQR]** | **MASLD** **(n=72)**  **Median [IQR]** | **MASH (n=36)**  **Median [IQR]** | **p-value**  **(NLO vs MASLD)** | **p-value**  **(NLO vs**  **MASH)** |
| --- | --- | --- | --- | --- | --- |
| Age | 47 [38, 52] | 49 [43, 57] | 50 [43, 57] | 0.1010 | 0.0989 |
| Sex (female) (N (%)) | 21 (80.8%) | 76 (83.3%) | 28 (77.8%) | 0.7314 | 1.0000 |
| Waist (cm) | 127 [120, 138] | 133 [121, 147] | 134 [122, 149] | 0.1057 | 0.0808 |
| Weight (Kg) | 124.6 [115.5, 144.6] | 129.6 [111.1, 151.6] | 129.3 [113.6, 149.5] | 0.7113 | 0.6277 |
| BMI (Kg/m2) | 43.4 [41.4, 46.5] | 47.6 [43.4, 52.4] | 47.6 [43.1, 50.5] | **0.0169** | 0.0507 |
| Systolic blood pressure (mmHg) | 118 [110, 133] | 131 [122, 135] | 132 [126, 138] | **0.0124** | **0.0069** |
| Diastolic blood pressure (mmHg) | 80 [77, 84] | 83 [79, 87] | 84 [81, 88] | **0.1020** | **0.0176** |
| Glucose (mmol/L) | 5.3 [4.9, 6.2] | 6.3 [5.5, 7.6] | 6.7 [5.7, 8.5] | **0.0023** | **0.0007** |
| Insulin (pmol/L) | 105 [81, 123] | 158 [98, 236] | 156 [110, 255] | **0.0042** | **0.0095** |
| HOMA-IR | 4.13 [2.67, 4.94] | 8.47 [4.56, 11.33] | 9.26 [4.44, 12.09] | **0.0023** | **0.0137** |
| HbA1c | 0.06 [0.05, 0.06] | 0.07 [0.06, 0.07] | 0.07 [0.06, 0.08] | **0.0029** | **0.0009** |
| Cholesterol (mmol/L) | 4.71 [3.63, 5.60] | 4.62 [3.90, 5.03] | 4.50 [3.94, 4.92] | 0.8456 | 0.7603 |
| Triglycerides (mmol/L) | 1.38 [0.94, 1.60] | 1.49 [1.28, 2.01] | 1.75 [1.41, 2.25] | 0.0575 | **0.0054** |
| LDL Cholesterol (mmol/L) | 2.81 [1.59, 3.73] | 2.63 [2.04, 3.21] | 2.63 [2.08, 3.28] | 0.6519 | 0.5965 |
| HDL Cholesterol (mmol/L) | 1.2 [0.92, 1.47] | 1.19 [0.99, 1.35] | 1.11 [0.99, 1.27] | 0.8867 | 0.6472 |
| ALP (U/L) | 82 [66, 96] | 81 [68, 96] | 83 [59, 96] | 0.8136 | 0.9542 |
| ALT (U/L) | 19 [16, 26] | 33 [24, 53] | 46 [34, 58] | **<0.001** | **<0.001** |
| AST (U/L) | 19 [17, 21] | 27 [19, 37] | 34 [24, 46] | **0.0003** | **<0.001** |

Data presented as median (interquartile; IQR).

Wilcoxon rank-sum test was used for group comparison. All p-values < 0.05 are significant

ALP, alkaline phosphatase; ALT, alanine transaminase; AST, aspartate aminotransferase; BMI, body mass index; HbA1c, hemoglobin A1c; HDL, high-density lipoprotein; HOMA-IR, homeostatic model of assessment for insulin resistance; LDL, low-density lipoprotein.

**Table S3: Comparison of anthropometric and biochemical data between MASLD** **subjects with no Fibrosis (F0) vs presence of fibrosis (F1-F2-F3-F4) and those with no fibrosis (F0) and those with severe fibrosis (F3-F4)**

| **Variable** | **F0 with MASLD** **(n=21)**  **Median [IQR]** | **F1-F2-F3-F4 with MASLD** **(n=51)**  **Median [IQR]** | **F3-F4 with MASLD**  **(n=15)**  **Median [IQR]** | **P-value**  **(F0 vs F1-F2-F3-F4)** | **p-value**  **(F0 vs F3-F4)** |
| --- | --- | --- | --- | --- | --- |
| Age | 46 [42, 56] | 50 [43, 57] | 56 [44, 58] | 0.2100 | 0.0516 |
| Sex female N (%) | 20 (95.2%) | 40 (78.4%) | 9 (60.0%) | 0.1606 | **0.0134** |
| Waist (cm) | 141 [122, 149] | 131 [120, 147] | 146 [123, 149] | 0.3642 | 0.7947 |
| Weight (Kg) | 133 [119, 152] | 129 [107, 147] | 141 [106, 155] | 0.2343 | 0.8599 |
| BMI (Kg/m2) | 50.1 [44.4, 55.5] | 47.0 [42.7, 51.5] | 47.5 [44.0, 50.5] | 0.1387 | 0.1727 |
| Systolic blood pressure (mmHg) | 127 [118, 133] | 132 [123, 136] | 133 [126, 134] | 0.0985 | 0.2073 |
| Diastolic blood pressure (mmHg) | 82 [77, 87] | 83 [79, 87] | 84 [79, 86] | 0.4221 | 0.6943 |
| Glucose (mmol/L) | 6.2 [5.2, 6.8] | 6.6 [5.6, 8.0] | 7.7 [6.1, 10.6] | 0.1674 | **0.0088** |
| Insulin (pmol/L) | 144 [120, 198] | 167 [95, 254] | 254 [160, 396] | 0.6425 | **0.0362** |
| HOMA-IR | 6.87 [4.61, 10.05] | 9.26 [4.56, 11.90] | 12.29 [6.10, 10.06] | 0.4263 | **0.0108** |
| HbA1c | 0.06 [0.05, 0.06] | 0.07 [0.06, 0.08] | 0.07 [0.07, 0.08] | **0.0163** | **0.0052** |
| Cholesterol (mmol/L) | 4.69 [3.94, 5.07] | 4.58 [3.88, 5.03] | 4.16 [3.74, 4.85] | 0.7076 | 0.3092 |
| Triglycerides (mmol/L) | 1.41 [0.93, 1.61] | 1.69 [1.30, 2.25] | 2.25 [1.86, 2.72] | 0.1078 | **0.0006** |
| LDL Cholesterol (mmol/L) | 2.63 [2.19, 3.23] | 2.69 [1.87, 3.21] | 2.29 [1.51, 2.96] | 0.8456 | 0.3092 |
| HDL Cholesterol (mmol/L) | 1.27 [1.09, 1.40] | 1.14 [0.98, 1.29] | 0.99 [0.89, 1.15] | 0.1844 | **0.0156** |
| ALP (U/L) | 77 [65, 99] | 82 [69, 94] | 82 [58, 99] | 0.5607 | 0.9361 |
| ALT (U/L) | 25 [21, 33] | 38 [25, 56] | 48 [36, 61] | **0.0135** | **0.0011** |
| AST (U/L) | 24 [19, 31] | 30 [20, 43] | 36 [25, 48] | 0.1255 | **0.0118** |

Data presented as median (interquartile; IQR) or as n (%).

Wilcoxon rank-sum test was used for group comparison. All p-values < 0.05 are significant.

ALP, alkaline phosphatase; ALT, alanine transaminase; AST, aspartate aminotransferase; BMI, body mass index; HbA1c, hemoglobin A1c; HDL, high-density lipoprotein; HOMA-IR, homeostatic model of assessment for insulin resistance; LDL, low-density lipoprotein.

**Table S4: Significant adipose tissue gene expression, plasma adipokines, cytokines, gut hormones, FGF-23, FGF-19 and endotoxin measures between Normal Liver Obese (NLO) vs MASLD** **subjects and NLO versus MASH subjects**

| **Variable** | **NLO (n=24)**  **Median [IQR]** | MASLD **(n=71)**  **Median [IQR]** | **MASH (n=36)**  **Median [IQR]** | **p-value**  **(NLO vs MASLD)** | **p-value**  **(NLO vs**  **MASH)** |
| --- | --- | --- | --- | --- | --- |
| PPAR g (∆Ct) | -045 (-1.01, 5.86) | -0.89 (-1.31, -0.43) |  | 0.0421 | N/A |
| S100a8 (∆Ct) | 0.39 (-0.38, 0.80) |  | 1.23 (-0.05, 1.72) | N/A | 0.0477 |
| Adiponectin 10,000 (pg/ml) | 656.70 (576.92, 860.62) | 593.79 (505.10, 704.17) | 569.57 (471.04, 702.88) | 0.0376 | 0.0323 |
| RBP4 per 10,000 (pg/ml) | 4000.80 (3245.45, 5003.10) | 4740.90 (4130.00, 6257.75) | 4740.90 (4256.30, 6326.10) | 0.0484 | 0.0352 |
| C-Reactive Protein (pg/mL) | 29212 (23296, 34809) |  | 40395 (32305, (54120) | N/A | 0.0097 |
| FGF19 (pg/mL) | 152.14 (87.14, 304.19) |  | 110.73 (73.56, 180.79) | N/A | 0.0482 |
| Endotoxin (EU/mL) | 0.00 (0.00, 0.08) | 0.33 (0.00, 5.65) | 0.03 (0.00, 1.46) | 0.0006 | 0.0287 |

Data presented as median (interquartile; IQR).

Wilcoxon rank-sum test was used for group comparison. All p-values < 0.05 are significant

**Table S5: Significant hepatic immune cells between Normal Liver Obese (NLO) vs MASLD** **subjects and NLO vs MASH subjects**

| **Immune Cell (umol/L)** | **NLO (n=26)**  **Median [IQR]** | **MASLD** **(n=72)**  **Median [IQR]** | **MASH (n=36)**  **Median [IQR]** | **p-value**  **(NLO vs MASLD)** | **p-value**  **(NLO vs MASH)** |
| --- | --- | --- | --- | --- | --- |
| Helper T Cells Total | 207.65 (160.71, 303.08) | 269.02 (220.19, 355.62) | 275.14 (236.94, 382.13) | 0.0153 | 0.0093 |
| CD4+ T Regulatory Cells Lobular | 0.73 (0.35, 1.55) | 1.61 (0.67, 2.79) | 1.91 (0.67, 3.63) | 0.0252 | 0.0103 |
| CD4+ T Regulatory Cells Portal | 16.82 (10.38, 31.10) | 33.68 (12.82, 68.94) | 38.06 (14.62, 70.45) | 0.0253 | 0.0197 |
| CD4+ T Regulatory Cells Total | 2.06 (1.10, 3.00) | 4.50 (2.00, 7.42) | 4.86 (2.58, 8.88) | 0.0022 | 0.0012 |
| Activated Macrophages Cells Lobular | 934.84 (746.92, 1,052.05) | 726.29 (535.00, 870.60) | 708.00 (535.00, 870.60) | 0.0116 | 0.0148 |
| Activated Macrophages Cells Total | 905.88 (719.31, 1,047.35) | 737.45 (528.43, 892.13) | 707.70 (532.39, 892.13) | 0.0322 | 0.0470 |
| B Cells Portal | 193.38 (124.35, 323.93) | 340.77 (211.57, 461.83) | 309.25 (213.75, 462.98) | 0.0110 | 0.0392 |
| B Cells Total | 37.53 (28.25, 50.55) | 51.39 (33.96, 67.12) |  | 0.0188 | N/A |
| Total Cells Lobular | 7,505 (6,704, 8,686) | 6,310 (5,391, 7,342) | 5,895 (5,112, 6,444) | 0.0005 | <0.0001 |
| Total Cells Portal | 14,653 (12,842, 15,665) |  | 14,097 (12,256, 15,906) | N/A | 0.0168 |
| Total Cells Total | 7,883 (7,152, 10,132) | 6,916 (6,237, 8,065) | 6,758 (5,970, 7,469) | 0.0022 | <0.0001 |

Data presented as median (interquartile; IQR).

Wilcoxon rank-sum test was used for group comparison. All p-values < 0.05 are significant.

**Table S6: Significant hepatic gene expression between Normal Liver Obese (NLO) vs MASLD** **subjects and NLO vs MASH subjects**

| **Gene Symbol** | **Gene Name** | **MASLD (n=59) vs NLO (n=24)** | | | **MASH (n=29) vs NLO (n=24)** | | |
| --- | --- | --- | --- | --- | --- | --- | --- |
|  |  | Log 2-fold change | p-value | p-adjusted | Log 2-fold change | p-value | p-adjusted |
| LPL | Lipoprotein Lipase | 2.8836 | 2.41E-12 | 5.91E-08 | 3.5114 | 2.27E-16 | 2.49E-12 |
| FABP4 | Fatty Acid Binding Protein 4 | 2.0847 | 4.05E-12 | 5.91E-08 | 2.6696 | 5.63E-21 | 1.23E-16 |
| MTND1P23 | MT-ND1 Pseudogene 23 | 3.2135 | 1.40E-08 | 1.02E-04 |  |  |  |
| AJ009632.2 | Uncharacterized LOC101927745 | 1.3752 | 6.18E-07 | 2.76E-03 | 1.5147 | 4.75E-08 | 1.04E-04 |
| MMP9 | Matrix Metallopeptidase 9 | 2.0034 | 6.62E-07 | 2.76E-03 | 2.5479 | 2.00E-09 | 7.30E-06 |
| PPP1R3G | Protein Phosphatase 1 Regulatory Subunit 3G | -1.2333 | 7.95E-07 | 2.90E-03 | -1.5249 | 5.49E-07 | 7.50E-04 |
| BOLA2B | bolA Family Member 2B | 3.8207 | 1.59E-06 | 4.23E-03 | 4.0372 | 3.99E-06 | 3.28E-03 |
| TREM2 | Triggering Receptor Expressed on Myeloid Cells 2 | 2.0076 | 1.98E-06 | 4.82E-03 | 2.6613 | 5.01E-15 | 3.65E-11 |
| BCAT1 | Branched Chain Amino Acid Transaminase 1 | 1.1267 | 2.33E-06 | 5.22E-03 | 1.5207 | 7.87E-11 | 4.30E-07 |
| ERBB4 | erb-b2 Receptor Tyrosine Kinase 4 | -2.3810 | 7.46E-06 | 1.36E-02 | -2.2732 | 1.58E-04 | 3.28E-02 |
| KCNH4 | Potassium Voltage-Gated Channel Subfamily H member 4 | 3.2411 | 4.24E-05 | 4.66E-02 |  |  |  |
| COL1A1 | Collagen Type I Alpha 1 Chain |  |  |  | 1.1136 | 1.41E-08 | 4.42E-05 |
| CAPG | Capping Actin Protein, Gelsolin Like |  |  |  | 1.2891 | 2.53E-08 | 6.91E-05 |
| FABP5 | Fatty Acid Binding Protein 5 |  |  |  | 1.2757 | 3.17E-08 | 7.71E-05 |
| STMN2 | Stathmin 2 |  |  |  | 4.1122 | 1.92E-07 | 3.24E-04 |
| CIDEC | Cell Death Inducing DFFA Like Effector c |  |  |  | 3.0548 | 4.93E-07 | 7.50E-04 |
| ATP6V0D2 | ATPase H+ Transporting V0 Subunit d2 |  |  |  | 4.1886 | 5.28E-07 | 7.50E-04 |
| KCNJ5 | Potassium Voltage-Gated Channel Subfamily J Member 5 |  |  |  | 1.5158 | 9.80E-07 | 1.13E-03 |
| FAM9B | Family with Sequence Similarity 9 Member B |  |  |  | -1.2090 | 2.53E-06 | 2.64E-03 |
| RPS6KL1 | Ribosomal Protein S6 Kinase Like 1 |  |  |  | 1.2852 | 3.06E-06 | 3.04E-03 |
| ADTRP | Androgen Dependent TFPI Regulating Protein |  |  |  | -1.6713 | 3.86E-06 | 3.28E-03 |
| IL32 | Interleukin 32 |  |  |  | 1.0220 | 4.73E-06 | 3.34E-03 |
| CXCL8 | C-X-C Motif Chemokine Ligand 8 |  |  |  | 1.8434 | 8.22E-06 | 4.99E-03 |
| SLC23A3 | Solute Carrier Family 23 Member 3 |  |  |  | 1.1421 | 1.54E-05 | 8.67E-03 |
| IGKV3-20 | Immunoglobulin Kappa Variable 3-20 |  |  |  | 1.0907 | 1.80E-05 | 9.42E-03 |
| FABP5P7 | Fatty Acid Binding Protein 5 Pseudogene 7 |  |  |  | 1.5555 | 1.81E-05 | 9.42E-03 |
| TENM3 | Teneurin Transmembrane Protein 3 |  |  |  | -1.1009 | 2.77E-05 | 1.29E-02 |
| AC011591.1 | Novel Transcript |  |  |  | -2.3745 | 2.77E-05 | 1.29E-02 |
| IGHG2 | Immunoglobulin Heavy Constant Gamma 2 |  |  |  | 1.2384 | 2.79E-05 | 1.29E-02 |
| SLITRK3 | SLIT and NTRK Like Family Member 3 |  |  |  | -1.7808 | 2.90E-05 | 1.29E-02 |
| TTC9 | Tetratricopeptide Repeat Domain 9 |  |  |  | 1.1615 | 3.07E-05 | 1.34E-02 |
| SLC1A7 | Solute Carrier Family 1 Member 7 |  |  |  | 1.2269 | 4.43E-05 | 1.68E-02 |
| SPATA21 | Spermatogenesis Associated 21 |  |  |  | 1.9909 | 5.92E-05 | 2.02E-02 |
| COL21A1 | Collagen Type XXI Alpha 1 Chain |  |  |  | -1.0426 | 7.16E-05 | 2.27E-02 |
| IGHA1 | Immunoglobulin Heavy Constant Alpha 1 |  |  |  | 1.1851 | 8.32E-05 | 2.56E-02 |
| SPP1 | Secreted Phosphoprotein 1 |  |  |  | 1.5842 | 8.85E-05 | 2.65E-02 |
| MMP12 | Matrix Metallopeptidase 12 |  |  |  | 3.7953 | 9.20E-05 | 2.65E-02 |
| MPO | Myeloperoxidase |  |  |  | 2.2600 | 9.83E-05 | 2.71E-02 |
| FAM95C | Family with Sequence Similarity 95 Member C |  |  |  | 1.0722 | 9.85E-05 | 2.71E-02 |
| ADAMDEC1 | ADAM Like Decysin 1 |  |  |  | 3.3120 | 9.96E-05 | 2.71E-02 |
| DHRS9 | Dehydrogenase/Reductase 9 |  |  |  | 1.0246 | 1.07E-04 | 2.73E-02 |
| IGFBP2 | Insulin Like Growth Factor Binding Protein 2 |  |  |  | -1.1555 | 1.14E-04 | 2.73E-02 |
| AKR1B10 | Aldo-Keto Reductase Family 1 Member B10 |  |  |  | 2.2374 | 1.15E-04 | 2.73E-02 |
| AC096577.1 | Novel Transcript |  |  |  | -1.9282 | 1.16E-04 | 2.73E-02 |
| TINCR | TINCR Ubiquitin Domain Containing |  |  |  | -2.6323 | 1.18E-04 | 2.73E-02 |
| ELANE | Elastase, Neutrophil Expressed |  |  |  | 2.2678 | 1.46E-04 | 3.20E-02 |
| TRIM31 | Tripartite Motif Containing 31 |  |  |  | 1.7162 | 1.48E-04 | 3.21E-02 |
| IGKV1-16 | Immunoglobulin Kappa Variable 1-16 |  |  |  | 1.4404 | 1.50E-04 | 3.22E-02 |
| PHLDA3 | Pleckstrin Homology Like Domain Family A Member 3 |  |  |  | 1.1313 | 1.64E-04 | 3.33E-02 |
| DUSP8 | Dual Specificity Phosphatase 8 |  |  |  | 1.4546 | 1.73E-04 | 3.39E-02 |
| HS3ST2 | heparan sulfate-glucosamine 3-sulfotransferase 2 [Source:HGNC Symbol;Acc:HGNC:5195] |  |  |  | 1.7037 | 1.74E-04 | 3.39E-02 |
| FLNC | filamin C [Source:HGNC Symbol;Acc:HGNC:3756] |  |  |  | -1.2670 | 1.82E-04 | 3.45E-02 |
| FNDC1 | Fibronectin Type III Domain Containing 1 |  |  |  | 1.0642 | 1.87E-04 | 3.52E-02 |
| IGLV6-57 | Immunoglobulin Lambda Variable 6-57 |  |  |  | 1.2725 | 2.04E-04 | 3.69E-02 |
| AP005131.3 | novel transcript, antisense C18orf1 |  |  |  | 2.0730 | 2.35E-04 | 4.08E-02 |
| MMP28 | Matrix Metallopeptidase 28 |  |  |  | -1.6042 | 2.55E-04 | 4.33E-02 |
| AL022724.1 | Novel Transcript |  |  |  | -2.1501 | 2.83E-04 | 4.62E-02 |
| IGLV1-44 | Immunoglobulin Lambda Variable 1-44 |  |  |  | 1.1660 | 2.92E-04 | 4.69E-02 |
| ART5 | ADP-Ribosyltransferase 5 |  |  |  | -2.4080 | 3.09E-04 | 4.81E-02 |
| CHIT1 | Chitinase 1 |  |  |  | 2.1774 | 3.18E-04 | 4.90E-02 |
| TNFRSF12A | TNF Receptor Superfamily Member 12A |  |  |  | 1.0331 | 3.24E-04 | 4.92E-02 |

**Table 7: Significant adipose tissue gene expression, plasma adipokines, cytokine, gut hormones, FGF-23, FGF-19 and endotoxin measures between MASLD** **subjects with no Fibrosis (F0) vs presence of fibrosis (F1-F2-F3-F4) and those with no fibrosis (F0) and those with severe fibrosis (F3-F4)**

| **Variable** | **F0 with** MASLD **(n=21)**  **Median [IQR]** | **F1-F2-F3-F4 with** MASLD **(n=51)**  **Median [IQR]** | **F3-F4 with** MASLD  **(n=15)**  **Median [IQR]** | **P-value**  **(F0 vs F1-F2-F3-F4)** | **p-value**  **(F0 vs F3-F4)** |
| --- | --- | --- | --- | --- | --- |
| S100a9 (∆Ct) | 0.43 (-0.74, 0.74) | 0.96 (0.28, 1.39) | 1.04 (0.28, 1.21) | 0.0261 | 0.0420 |
| Leptin (pg/ml) | 70683 (47188, 104718) |  | 26914 (21355, 44432) | N/A | 0.0086 |
| Adiponectin 10,000 (pg/ml) | 680.56 (568.12, 732.35) | 561.05 (471.04, 678.83) | 542.65 (433.29, 646.19) | 0.0429 | 0.0248 |

Data presented as median (interquartile; IQR).

Wilcoxon rank-sum test was used for group comparison. All p-values < 0.05 are significant

**Table S8: Significant hepatic immune cells between MASLD** **subjects with no Fibrosis (F0) vs presence of fibrosis (F1-F2-F3-F4) and those with no fibrosis (F0) and those with severe fibrosis (F3-F4)**

| Variable | F0 with MASLD (n=21)  Median [IQR] | F1-F2-F3-F4 with MASLD (n=51)  Median [IQR] | F3-F4 with MASLD  (n=15)  Median [IQR] | P-value  (F0 vs F1-F2-F3-F4) | p-value  (F0 vs F3-F4) |
| --- | --- | --- | --- | --- | --- |
| Tissue Area Total | 0.60 (0.56, 0.62) |  | 0.62 (0.61, 0.63) | N/A | 0.0302 |
| Helper T Cells Lobular | 89.42 (73.45, 122.45) |  | 117.89 (101.34, 164.55) | N/A | 0.0492 |
| Helper T Cells Total | 297.14 (185.41, 358.69) |  | 487.52 (282.52, 603.53) | N/A | 0.0150 |
| NK Cells Portal | 138.03 (71.10, 243.51) |  | 62.45 (32.02, 113.66) | N/A | 0.0278 |
| Possible NKT Cells Portal | 64.41 (40.24, 103.92) | 42.21 (25.47, 75.25) |  | 0.0476 | N/A |
| B Cells Total | 50.91 (33.20, 63.52) |  | 73.78 (44.34, 90.30) | N/A | 0.0077 |
| Total Cells Portal | 15191 (11947, 16387) |  | 14823 (14097, 16533) | N/A | 0.0073 |

Data presented as median (interquartile; IQR).

Wilcoxon rank-sum test was used for group comparison. All p-values < 0.05 are significant

**Table S9: Significant hepatic gene expression between MASLD** **subjects with no Fibrosis (F0) vs presence of fibrosis (F1-F2-F3-F4) and those with no fibrosis (F0) and those with severe fibrosis (F3-F4)**

| **Gene Symbol** | **Gene Name** | **Presence of Fibrosis (n=42) vs No Fibrosis (n=17)** | | | **Severe Fibrosis (n=10) vs No Fibrosis (n=17)** | | |
| --- | --- | --- | --- | --- | --- | --- | --- |
|  |  | Log 2-fold change | p-value | p-adjusted | Log 2-fold change | p-value | p-adjusted |
| MTND1P23 | MT-ND1 Pseudogene 23 | -7.66607 | 8.11E-37 | 1.65E-32 | -7.1136 | 3.07E-10 | 5.93E-07 |
| USP9Y | Ubiquitin Specific Peptidase 9 Y-Linked | 23.89198 | 6.17E-17 | 4.29E-13 | 24.6667 | 3.80E-16 | 2.20E-12 |
| UTY | Ubiquitously Transcribed Tetratricopeptide Repeat Containing, Y-Linked | 23.78312 | 6.72E-17 | 4.29E-13 | 24.5136 | 5.77E-16 | 2.51E-12 |
| TXLNGY | Taxilin Gamma Pseudogene, Y-Linked | 24.91236 | 8.45E-17 | 4.29E-13 | 25.6522 | 2.44E-17 | 2.82E-13 |
| KDM5D | Lysine Demethylase 5D | 24.82113 | 1.37E-14 | 5.55E-11 | 25.5513 | 3.25E-17 | 2.82E-13 |
| ZFY | Zinc Finger Protein Y-Linked | 23.08390 | 2.68E-14 | 9.06E-11 | 23.8686 | 3.26E-15 | 1.13E-11 |
| EIF1AY | Eukaryotic Translation Initiation Factor 1A Y-Linked | 22.21921 | 4.47E-13 | 1.30E-09 | 22.3039 | 1.78E-13 | 3.87E-10 |
| AEBP1 | AE Binding Protein 1 | 1.05607 | 8.42E-07 | 1.90E-03 | 1.6391 | 3.40E-07 | 3.70E-04 |
| MOXD1 | Monooxygenase DBH Like 1 | 2.06319 | 3.51E-06 | 6.49E-03 | 3.1427 | 1.86E-06 | 1.20E-03 |
| PHLDA3 | Pleckstrin Homology Like Domain Family A Member 3 | 1.27371 | 3.39E-05 | 4.91E-02 | 2.1799 | 4.12E-14 | 1.02E-10 |
| NLGN4Y | Neuroligin 4 Y-Linked |  |  |  | 23.0415 | 2.82E-14 | 8.17E-11 |
| GAS6-AS1 | GAS6 Antisense RNA 1 |  |  |  | 1.7001 | 3.69E-09 | 6.42E-06 |
| AEN | Apoptosis Enhancing Nuclease |  |  |  | 1.1563 | 1.93E-08 | 3.05E-05 |
| FBLN5 | Fibulin 5 |  |  |  | 1.2926 | 2.12E-08 | 3.07E-05 |
| ADAMTSL2 | ADAMTS Like 2 |  |  |  | 1.1167 | 3.32E-08 | 4.44E-05 |
| STMN2 | Stathmin 2 |  |  |  | 5.1409 | 1.44E-07 | 1.67E-04 |
| KRT7 | Keratin 7 |  |  |  | 1.1886 | 6.97E-07 | 6.73E-04 |
| MMP2 | Matrix Metallopeptidase 2 |  |  |  | 1.2274 | 1.13E-06 | 1.03E-03 |
| ISLR | Immunoglobulin Superfamily Containing Leucine Rich Repeat |  |  |  | 1.2967 | 1.45E-06 | 1.12E-03 |
| CCL21 | C-C Motif Chemokine Ligand 21 |  |  |  | 1.3333 | 1.50E-06 | 1.12E-03 |
| VWF | von Willebrand Factor |  |  |  | 1.1763 | 1.73E-06 | 1.20E-03 |
| PODN | Podocan |  |  |  | 1.6862 | 3.18E-06 | 1.91E-03 |
| CXorf36 | Chromosome X Open Reading Frame 36 |  |  |  | 1.3703 | 5.86E-06 | 3.08E-03 |
| PDGFRA | Platelet Derived Growth Factor Receptor Alpha |  |  |  | 1.0465 | 6.49E-06 | 3.32E-03 |
| DKK 3.00 | Dickkopf WNT Signaling Pathway Inhibitor 3 |  |  |  | 1.3356 | 6.70E-06 | 3.33E-03 |
| LUM | Lumican |  |  |  | 1.4487 | 7.13E-06 | 3.44E-03 |
| HYDIN | HYDIN, Axonemal Central Pair Apparatus Protein |  |  |  | -2.2659 | 9.59E-06 | 4.16E-03 |
| ITGBL1 | Integrin Subunit Beta Like 1 |  |  |  | 2.2536 | 9.81E-06 | 4.16E-03 |
| THBS2 | Thrombospondin 2 |  |  |  | 1.6234 | 1.04E-05 | 4.31E-03 |
| GPC3 | Glypican 3 |  |  |  | 1.5884 | 1.64E-05 | 6.35E-03 |
| RPS4Y1 | Ribosomal Protein S4 Y-Linked 1 |  |  |  | 12.9019 | 2.04E-05 | 7.54E-03 |
| MFAP4 | Microfibril Associated Protein 4 |  |  |  | 1.0902 | 2.19E-05 | 7.77E-03 |
| SAMD11 | Sterile Alpha Motif Domain Containing 11 |  |  |  | 1.6668 | 2.73E-05 | 8.78E-03 |
| COL1A1 | Collagen Type I Alpha 1 Chain |  |  |  | 1.2530 | 3.02E-05 | 9.40E-03 |
| FMOD | Fibromodulin |  |  |  | 1.0522 | 3.06E-05 | 9.40E-03 |
| MIR34AHG | MIR34A Host Gene |  |  |  | 1.1016 | 5.13E-05 | 1.39E-02 |
| C7 | Complement C7 |  |  |  | 1.1919 | 5.80E-05 | 1.51E-02 |
| EFEMP1 | EGF Containing Fibulin Extracellular Matrix Protein 1 |  |  |  | 1.7231 | 6.72E-05 | 1.67E-02 |
| GPAT3 | Glycerol-3-Phosphate Acyltransferase 3 |  |  |  | -1.0775 | 6.98E-05 | 1.69E-02 |
| DPT | Dermatopontin |  |  |  | 1.7769 | 7.22E-05 | 1.72E-02 |
| THY1 | Thy-1 Cell Surface Antigen |  |  |  | 2.0125 | 7.90E-05 | 1.83E-02 |
| ALKAL2 | ALK and LTK Ligand 2 |  |  |  | 1.4798 | 8.59E-05 | 1.96E-02 |
| DDX3Y | DEAD-Box Helicase 3 Y-Linked |  |  |  | 11.7640 | 1.02E-04 | 2.22E-02 |
| AC021242.1 | DENN/MADD Domain Containing 4A (DENND4A) Pseudogene |  |  |  | -1.1243 | 1.05E-04 | 2.22E-02 |
| AC002401.3 | Novel Transcript, Antisense to PDK2 |  |  |  | -1.5243 | 1.13E-04 | 2.33E-02 |
| MGP | Matrix Gla Protein |  |  |  | 1.4175 | 1.13E-04 | 2.33E-02 |
| ALDH1A3 | Aldehyde Dehydrogenase 1 Family Member A3 |  |  |  | 1.1978 | 1.18E-04 | 2.33E-02 |
| LXN | Latexin |  |  |  | 1.2112 | 1.21E-04 | 2.36E-02 |
| CXCL6 | C-X-C Motif Chemokine Ligand 6 |  |  |  | 1.6304 | 1.25E-04 | 2.39E-02 |
| ISG20 | Interferon Stimulated Exonuclease Gene 20 |  |  |  | 1.1298 | 1.25E-04 | 2.39E-02 |
| PTGDS | Prostaglandin D2 Synthase |  |  |  | 1.6726 | 1.28E-04 | 2.42E-02 |
| SEC24AP1 | SEC24 Homolog A Pseudogene 1 |  |  |  | -1.3584 | 1.54E-04 | 2.70E-02 |
| AC122688.3 | Novel Transcript |  |  |  | 2.4394 | 1.60E-04 | 2.72E-02 |
| COL1A2 | Collagen Type I Alpha 2 Chain |  |  |  | 1.0501 | 1.61E-04 | 2.72E-02 |
| LAMA2 | Laminin Subunit Alpha 2 |  |  |  | 1.3971 | 1.90E-04 | 3.03E-02 |
| COL28A1 | Collagen Type XXVIII Alpha 1 Chain |  |  |  | -1.9815 | 1.94E-04 | 3.03E-02 |
| SOX9 | SRY-Box 9 |  |  |  | 1.0169 | 2.11E-04 | 3.21E-02 |
| EPHA3 | EPH Receptor A3 |  |  |  | 1.4922 | 2.19E-04 | 3.29E-02 |
| GLI2 | GLI Family Zinc Finger 2 |  |  |  | 2.1877 | 2.28E-04 | 3.34E-02 |
| SOD3 | Superoxide Dismutase 3 |  |  |  | 1.1557 | 2.35E-04 | 3.39E-02 |
| TNFRSF12A | TNF Receptor Superfamily Member 12A |  |  |  | 1.2805 | 2.36E-04 | 3.39E-02 |
| PLCXD3 | Phosphatidylinositol Specific Phospholipase C X Domain Containing 3 |  |  |  | 1.1072 | 2.38E-04 | 3.39E-02 |
| CLDN10 | Claudin 10 |  |  |  | 1.0446 | 2.76E-04 | 3.69E-02 |
| EPCAM | Epithelial Cell Adhesion Molecule |  |  |  | 1.4475 | 3.24E-04 | 4.20E-02 |
| MDK | Midkine |  |  |  | 1.0038 | 3.32E-04 | 4.22E-02 |
| SPATA21 | Spermatogenesis Associated 21 |  |  |  | 2.0814 | 3.37E-04 | 4.22E-02 |
| LMCD1 | LIM and Cysteine Rich Domains 1 |  |  |  | 1.0739 | 3.38E-04 | 4.22E-02 |
| LAMC3 | Laminin Subunit Gamma 3 |  |  |  | 1.6530 | 3.43E-04 | 4.23E-02 |
| IFI44L | Interferon Induced Protein 44 Like |  |  |  | -1.1616 | 3.61E-04 | 4.30E-02 |
| AJAP1 | Adherens Junctions Associated Protein 1 |  |  |  | 2.0829 | 3.65E-04 | 4.30E-02 |
| CLSTN2 | Calsyntenin 2 |  |  |  | 1.4059 | 3.83E-04 | 4.41E-02 |
| PDLIM3 | PDZ and LIM Domain 3 |  |  |  | 1.4234 | 4.28E-04 | 4.67E-02 |
| CBLN4 | Cerebellin 4 Precursor |  |  |  | -1.2937 | 4.33E-04 | 4.68E-02 |
| AC093525.7 | Novel Transcript, Antisense to PDPK1 |  |  |  | -1.8643 | 4.34E-04 | 4.68E-02 |
| VIL1 | Villin 1 |  |  |  | -1.1115 | 4.46E-04 | 4.76E-02 |
| LTBP2 | Catent Transforming Growth Factor Beta Binding Protein 2 |  |  |  | 1.3548 | 4.53E-04 | 4.77E-02 |
| CCDC80 | Coiled-Coil Domain Containing 80 |  |  |  | 1.5065 | 4.64E-04 | 4.80E-02 |

**Table S10: Significant KEGG pathways in MASLD vs NLO**

| ID | Description | p-value | Adjusted p-value |
| --- | --- | --- | --- |
| hsa04610 | Complement and coagulation cascades | 1.03E-13 | 3.42E-11 |
| hsa04514 | Cell adhesion molecules | 1.85E-05 | 0.00308155 |
| hsa05322 | Systemic lupus erythematosus | 0.00032217 | 0.03576102 |
| hsa05150 | Staphylococcus aureus infection | 0.00063883 | 0.05318264 |

Adjusted p value <0.05 is significant.

**Table S11: Significant KEGG pathways in MASH vs NLO**

| ID | Description | p-value | Adjusted p-value |
| --- | --- | --- | --- |
| hsa04610 | Complement and coagulation cascades | 5.21E-18 | 1.78E-15 |
| hsa04974 | Protein digestion and absorption | 1.73E-08 | 2.95E-06 |
| hsa04514 | Cell adhesion molecules | 7.45E-06 | 0.00084943 |
| hsa04933 | AGE-RAGE signaling pathway in diabetic complications | 2.50E-05 | 0.00214155 |
| hsa05150 | Staphylococcus aureus infection | 4.17E-05 | 0.00235696 |
| hsa04512 | ECM-receptor interaction | 4.20E-05 | 0.00235696 |
| hsa05133 | Pertussis | 4.82E-05 | 0.00235696 |
| hsa04115 | p53 signaling pathway | 0.00011683 | 0.0049944 |
| hsa04151 | PI3K-Akt signaling pathway | 0.0001575 | 0.00598507 |
| hsa05146 | Amoebiasis | 0.00030694 | 0.01049738 |
| hsa04145 | Phagosome | 0.00041316 | 0.01238321 |
| hsa04510 | Focal adhesion | 0.0004345 | 0.01238321 |
| hsa05200 | Pathways in cancer | 0.00054622 | 0.0143698 |
| hsa05222 | Small cell lung cancer | 0.00062139 | 0.01517958 |
| hsa05202 | Transcriptional misregulation in cancer | 0.00170987 | 0.03898498 |

Adjusted p value <0.05 is significant.

**Table S12: Significant KEGG pathways in MASLD with presence of fibrosis and no fibrosis**

| ID | Description | p-value | Adjusted p-value |
| --- | --- | --- | --- |
| hsa04512 | ECM-receptor interaction | 3.04E-06 | 0.00096367 |
| hsa04974 | Protein digestion and absorption | 2.12E-05 | 0.0026852 |
| hsa04510 | Focal adhesion | 2.54E-05 | 0.0026852 |
| hsa04933 | AGE-RAGE signaling pathway in diabetic complications | 5.81E-05 | 0.00459074 |
| hsa05146 | Amoebiasis | 7.34E-05 | 0.00459074 |
| hsa05144 | Malaria | 8.69E-05 | 0.00459074 |
| hsa04115 | p53 signaling pathway | 0.00014886 | 0.0067411 |
| hsa04151 | PI3K-Akt signaling pathway | 0.00032895 | 0.01303474 |
| hsa05165 | Human papillomavirus infection | 0.00038062 | 0.01340631 |
| hsa00910 | Nitrogen metabolism | 0.00085343 | 0.02705365 |
| hsa05202 | Transcriptional misregulation in cancer | 0.00172495 | 0.04691158 |
| hsa05218 | Melanoma | 0.00180942 | 0.04691158 |
| hsa04611 | Platelet activation | 0.00192382 | 0.04691158 |

Adjusted p value <0.05 is significant.

**Table S13: Significant KEGG pathways in MASLD with severe fibrosis and no fibrosis**

| ID | Description | pvalue | p.adjust |
| --- | --- | --- | --- |
| hsa04510 | Focal adhesion | 2.04E-10 | 6.91E-08 |
| hsa04512 | ECM-receptor interaction | 1.51E-09 | 2.56E-07 |
| hsa04151 | PI3K-Akt signaling pathway | 5.74E-07 | 6.49E-05 |
| hsa01100 | Metabolic pathways | 8.42E-06 | 0.00071352 |
| hsa05222 | Small cell lung cancer | 1.28E-05 | 0.00086738 |
| hsa04974 | Protein digestion and absorption | 2.96E-05 | 0.00147954 |
| hsa05165 | Human papillomavirus infection | 3.06E-05 | 0.00147954 |
| hsa00590 | Arachidonic acid metabolism | 3.86E-05 | 0.00163673 |
| hsa04115 | p53 signaling pathway | 4.82E-05 | 0.00181674 |
| hsa05205 | Proteoglycans in cancer | 0.0001101 | 0.00373226 |
| hsa00071 | Fatty acid degradation | 0.00012616 | 0.00388795 |
| hsa00240 | Pyrimidine metabolism | 0.00027205 | 0.00768537 |
| hsa00982 | Drug metabolism - cytochrome P450 | 0.00035557 | 0.00927227 |
| hsa00380 | Tryptophan metabolism | 0.0004101 | 0.00957098 |
| hsa00280 | Valine, leucine and isoleucine degradation | 0.00042349 | 0.00957098 |
| hsa05410 | Hypertrophic cardiomyopathy | 0.00074666 | 0.01581988 |
| hsa04390 | Hippo signaling pathway | 0.00088684 | 0.01732548 |
| hsa05218 | Melanoma | 0.00106625 | 0.01732548 |
| hsa01521 | EGFR tyrosine kinase inhibitor resistance | 0.00110537 | 0.01732548 |
| hsa04061 | Viral protein interaction with cytokine and cytokine receptor | 0.00111527 | 0.01732548 |
| hsa04933 | AGE-RAGE signaling pathway in diabetic complications | 0.00111527 | 0.01732548 |
| hsa04610 | Complement and coagulation cascades | 0.00112437 | 0.01732548 |
| hsa05200 | Pathways in cancer | 0.00136776 | 0.02015957 |
| hsa05146 | Amoebiasis | 0.00143952 | 0.02033323 |
| hsa05412 | Arrhythmogenic right ventricular cardiomyopathy | 0.00224035 | 0.03037914 |
| hsa00430 | Taurine and hypotaurine metabolism | 0.00263415 | 0.03434528 |
| hsa00120 | Primary bile acid biosynthesis | 0.00374481 | 0.04668198 |
| hsa05414 | Dilated cardiomyopathy | 0.00385574 | 0.04668198 |
| hsa00620 | Pyruvate metabolism | 0.00409117 | 0.04782441 |

Adjusted p value <0.05 is significant.

**Table S14:** Bray-Curtis diversity ADONIS results indicate that sample type (fecal, hepatic, adipose) accounts for the majority of the variance observed between samples (p<0.01, R2 = ~0.32)

| **ADONIS NLO vs MASLD All Unscaled** | | | | | | |
| --- | --- | --- | --- | --- | --- | --- |
|  | Df | Sum of Squares | F Model | R2 | p-value | p-adjusted |
| Sample Type | 2 | 30.341 | 68.158 | 0.33 | 0.001 | **0.0254** |
| HbA1c | 1 | 0.32 | 1.437 | 0 | 0.125 | 0.5299 |
| MASLD | 1 | 0.528 | 2.372 | 0.01 | 0.029 | 0.3688 |
| Sex | 1 | 0.228 | 1.026 | 0 | 0.286 | 0.8083 |
| Age | 1 | 0.3 | 1.349 | 0 | 0.147 | 0.5341 |
| SubjectID | 86 | 20.547 | 1.073 | 0.22 | 0.117 | 0.5299 |
| Sample Type * HbA1c | 2 | 0.634 | 1.424 | 0.01 | 0.098 | 0.5299 |
| Sample Type * MASLD | 2 | 0.744 | 1.672 | 0.01 | 0.044 | 0.3731 |
| Sample Type * MASLD * HbA1c | 2 | 0.554 | 1.244 | 0.01 | 0.185 | 0.5882 |
| Residual | 174 | 38.729 | NA | 0.42 | NA | NA |
| Total | 272 | 92.925 | NA | 1 | NA | NA |
| **ADONIS NLO vs MASH All Unscaled** | | | | | | |
|  | Df | Sum of Squares | F Model | R2 | p-value | p-adjusted |
| Sample Type | 2 | 18.792 | 43.615 | 0.34 | 0.001 | **0.0254** |
| HbA1c | 1 | 0.345 | 1.6 | 0.01 | 0.098 | 0.4985 |
| MASH | 1 | 0.488 | 2.264 | 0.01 | 0.034 | 0.4324 |
| Sex | 1 | 0.253 | 1.174 | 0 | 0.228 | 0.7249 |
| Age | 1 | 0.333 | 1.544 | 0.01 | 0.119 | 0.5045 |
| SubjectID | 50 | 11.29 | 1.048 | 0.2 | 0.3 | 0.8478 |
| Sample Type * HbA1c | 2 | 0.674 | 1.565 | 0.01 | 0.076 | 0.4833 |
| Sample Type * MASH | 2 | 0.702 | 1.63 | 0.01 | 0.059 | 0.4833 |
| Sample Type * MASH * HbA1c | 2 | 0.581 | 1.349 | 0.01 | 0.152 | 0.5523 |
| Residual | 102 | 21.974 | NA | 0.4 | NA | NA |
| Total | 164 | 55.431 | NA | 1 | NA | NA |
| **ADONIS F0 with MASLD vs F1-F2-F3-F4 with MASLD All Unscaled** | | | | | | |
|  | Df | Sum of Squares | F Model | R2 | p-value | p-adjusted |
| Sample Type | 2 | 22.784 | 49.908 | 0.32 | 0.001 | **0.0254** |
| HbA1c | 1 | 0.266 | 1.165 | 0 | 0.235 | 0.8539 |
| F1-F2-F3-F4 | 1 | 0.579 | 2.535 | 0.01 | 0.017 | 0.2162 |
| Sex | 1 | 0.253 | 1.107 | 0 | 0.276 | 0.8775 |
| Age | 1 | 0.281 | 1.232 | 0 | 0.21 | 0.8539 |
| SubjectID | 64 | 15.505 | 1.061 | 0.22 | 0.203 | 0.8539 |
| Sample Type * HbA1c | 2 | 0.543 | 1.19 | 0.01 | 0.213 | 0.8539 |
| Sample Type * F1-F2-F3-F4 | 2 | 0.654 | 1.433 | 0.01 | 0.078 | 0.6613 |
| Sample Type * F1-F2-F3-F4 * HbA1c | 2 | 0.424 | 0.928 | 0.01 | 0.487 | 1 |
| Residual | 130 | 29.674 | NA | 0.42 | NA | NA |
| Total | 206 | 70.963 | NA | 1 | NA | NA |
| **ADONIS F0 with MASLD vs F3-F4 with MASLD All Unscaled** | | | | | | |
|  | Df | Sum of Squares | F Model | R2 | p-value | p-adjusted |
| Sample Type | 2 | 12.037 | 26.502 | 0.33 | 0.001 | **0.0254** |
| HbA1c | 1 | 0.262 | 1.152 | 0.01 | 0.229 | 1 |
| F3-F4 | 1 | 0.486 | 2.14 | 0.01 | 0.039 | 0.496 |
| Sex | 1 | 0.28 | 1.234 | 0.01 | 0.184 | 1 |
| Age | 1 | 0.231 | 1.017 | 0.01 | 0.341 | 1 |
| SubjectID | 31 | 7.451 | 1.058 | 0.2 | 0.29 | 1 |
| Sample Type * HbA1c | 2 | 0.501 | 1.103 | 0.01 | 0.274 | 1 |
| Sample Type * F3-F4 | 2 | 0.602 | 1.325 | 0.02 | 0.151 | 1 |
| Sample Type * F3-F4 * HbA1c | 2 | 0.417 | 0.917 | 0.01 | 0.534 | 1 |
| Residual | 64 | 14.534 | NA | 0.39 | NA | NA |
| Total | 107 | 36.8 | NA | 1 | NA | NA |

**Table S15:** Chao1 alpha diversity differs significantly between NLO and MASLD for stool, adipose, and liver tissues and between NLO and MASH for adipose tissues (p < 0.05)

| **Stool** | **Sum Sq** | **Df** | **p-value** | **p- adjusted** |
| --- | --- | --- | --- | --- |
|  |  |  |  |  |
| MASLD | 1516 | 1 | 2 | 0.16 |
| HbA1c | 2740 | 1 | 4 | 0.06 |
| MASLD * HbA1c | 3060 | 1 | 4 | 0.05 |
| Residuals | 64817 | 87 | NA | NA |
|  |  |  |  |  |
| MASH | 1648 | 1 | 2 | 0.13 |
| HbA1c | 2137 | 1 | 3 | 0.08 |
| MASH * HbA1c | 3174 | 1 | 5 | **0.04** |
| Residuals | 35066 | 51 | NA | NA |
|  |  |  |  |  |
| F1-F2-F3-F4 | 96 | 1 | 0 | 0.7 |
| HbA1c | 817 | 1 | 1 | 0.26 |
| F1-F2-F3-F4 * HbA1c | 125 | 1 | 0 | 0.66 |
| Residuals | 41218 | 65 | NA | NA |
|  |  |  |  |  |
| F3-F4 | 8 | 1 | 0 | 0.92 |
| HbA1c | 0 | 1 | 0 | 0.98 |
| F3-F4 * HbA1c | 1008 | 1 | 1 | 0.25 |
| Residuals | 23408 | 32 | NA | NA |
| **Adipose** | **Sum Sq** | **Df** | **p-value** | **p adjusted** |
|  |  |  |  |  |
| MASLD | 521 | 1 | 1 | 0.29 |
| HbA1c | 1892 | 1 | 4 | 0.05 |
| MASLD * HbA1c | 59 | 1 | 0 | 0.72 |
| Residuals | 33952 | 73 | NA | NA |
|  |  |  |  |  |
| MASH | 1142 | 1 | 3 | 0.11 |
| HbA1c | 1580 | 1 | 4 | 0.06 |
| MASH * HbA1c | 38 | 1 | 0 | 0.77 |
| Residuals | 17405 | 41 | NA | NA |
|  |  |  |  |  |
| F1-F2-F3-F4 | 19 | 1 | 0 | 0.85 |
| HbA1c | 1351 | 1 | 3 | 0.1 |
| F1-F2-F3-F4 * HbA1c | 396 | 1 | 1 | 0.37 |
| Residuals | 25655 | 52 | NA | NA |
|  |  |  |  |  |
| F3-F4 | 20 | 1 | 0 | 0.81 |
| HbA1c | 769 | 1 | 2 | 0.14 |
| F3-F4 * HbA1c | 454 | 1 | 1 | 0.26 |
| Residuals | 7770 | 23 | NA | NA |
| **Liver** | **Sum Sq** | **Df** | **p-value** | **p adjusted** |
|  |  |  |  |  |
| MASLD | 4061 | 1 | 7 | **0.01** |
| HbA1c | 88 | 1 | 0 | 0.69 |
| MASLD * HbA1c | 2858 | 1 | 5 | **0.03** |
| Residuals | 36811 | 67 | NA | NA |
|  |  |  |  |  |
| MASH | 4191 | 1 | 11 | **0.01** |
| HbA1c | 129 | 1 | 0 | 0.57 |
| MASH * HbA1c | 2009 | 1 | 5 | **0.03** |
| Residuals | 14679 | 38 | NA | NA |
|  |  |  |  |  |
| F1-F2-F3-F4 | 255 | 1 | 0 | 0.52 |
| HbA1c | 1154 | 1 | 2 | 0.17 |
| F1-F2-F3-F4 * HbA1c | 915 | 1 | 2 | 0.23 |
| Residuals | 30351 | 50 | NA | NA |
|  |  |  |  |  |
| F3-F4 | 212 | 1 | 0 | 0.5 |
| HbA1c | 7 | 1 | 0 | 0.9 |
| F3-F4 * HbA1c | 132 | 1 | 0 | 0.59 |
| Residuals | 9252 | 21 | NA | NA |

**Table S16:** Differentially abundant genera between tissues (adipose and liver, positive coef values) and stool (negative coef values), arranged by descending adjusted p-value (MaAsLin2 p < 0.05). The genera with the most substantial differences included *Pseudomonas, Corynebacterium, Halomonas*, and *Bacillaceae*, which were significantly enriched in tissue samples, and *Blautia, Anaerostipes, Ruminoccus, Butyricicoccus*, and *Eubacterium,* which were significantly enriched in stool

| **feature** | **coef** | **stderr** | **pval** | **qval** | **N** | **N.not. Zero** |
| --- | --- | --- | --- | --- | --- | --- |
| Halomonas | 4.395 | 0.065 | 6.78E-172 | 1.16E-169 | 273 | 182 |
| Bacillus | 4.483 | 0.068 | 3.47E-168 | 2.96E-166 | 273 | 182 |
| Corynebacterium | 5.167 | 0.093 | 6.24E-150 | 3.55E-148 | 273 | 182 |
| Family_Bacillaceae | 5.855 | 0.109 | 2.43E-146 | 1.04E-144 | 273 | 182 |
| Pseudomonas | 4.616 | 0.089 | 1.31E-142 | 4.47E-141 | 273 | 182 |
| Blautia | -6.891 | 0.160 | 6.01E-123 | 1.71E-121 | 273 | 138 |
| X.Ruminococcus._torques_group | -4.837 | 0.140 | 4.10E-101 | 1.00E-99 | 273 | 95 |
| Anaerostipes | -4.882 | 0.147 | 1.12E-97 | 2.39E-96 | 273 | 94 |
| Butyricicoccus | -3.205 | 0.114 | 1.08E-82 | 1.85E-81 | 273 | 92 |
| Incertae_Sedis | -3.689 | 0.131 | 9.97E-83 | 1.85E-81 | 273 | 95 |
| Fusicatenibacter | -4.881 | 0.177 | 1.03E-80 | 1.59E-79 | 273 | 102 |
| X.Eubacterium._hallii_group | -5.032 | 0.184 | 1.00E-79 | 1.43E-78 | 273 | 87 |
| Dorea | -4.789 | 0.199 | 1.98E-69 | 2.60E-68 | 273 | 90 |
| Family_Pasteurellaceae | 2.866 | 0.124 | 2.66E-66 | 3.25E-65 | 273 | 162 |
| Coprococcus | -4.147 | 0.180 | 1.15E-65 | 1.32E-64 | 273 | 89 |
| Neisseria | 2.903 | 0.129 | 1.11E-63 | 1.18E-62 | 273 | 164 |
| Lachnoclostridium | -3.507 | 0.157 | 1.28E-63 | 1.28E-62 | 273 | 107 |
| Agathobacter | -4.820 | 0.215 | 1.51E-63 | 1.43E-62 | 273 | 122 |
| Monoglobus | -2.770 | 0.127 | 1.55E-61 | 1.40E-60 | 273 | 80 |
| Subdoligranulum | -4.354 | 0.205 | 8.74E-60 | 7.47E-59 | 273 | 91 |
| Prevotella | 2.528 | 0.124 | 8.71E-57 | 7.10E-56 | 273 | 162 |
| Family_XIII_AD3011_group | -2.275 | 0.114 | 2.17E-55 | 1.69E-54 | 273 | 76 |
| Faecalibacterium | -4.592 | 0.243 | 2.12E-51 | 1.57E-50 | 273 | 116 |
| Collinsella | -4.351 | 0.235 | 5.79E-50 | 4.13E-49 | 273 | 88 |
| Micrococcus | 2.893 | 0.160 | 2.25E-48 | 1.54E-47 | 273 | 149 |
| Acinetobacter | 2.666 | 0.148 | 4.21E-48 | 2.77E-47 | 273 | 154 |
| Family_Lachnospiraceae | -3.169 | 0.186 | 7.31E-45 | 4.63E-44 | 273 | 167 |
| X.Ruminococcus._gauvreauii_group | -3.192 | 0.187 | 1.01E-44 | 6.16E-44 | 273 | 76 |
| X.Eubacterium._coprostanoligenes_group | -2.972 | 0.181 | 1.08E-42 | 6.37E-42 | 273 | 102 |
| Eggerthella | -2.242 | 0.138 | 9.54E-42 | 5.44E-41 | 273 | 71 |
| Sphingomonas | 2.407 | 0.150 | 2.39E-41 | 1.32E-40 | 273 | 134 |
| Flavobacterium | 2.716 | 0.170 | 8.06E-41 | 4.30E-40 | 273 | 127 |
| Alloprevotella | 2.022 | 0.128 | 1.81E-40 | 9.37E-40 | 273 | 142 |
| Prevotella_7 | 2.150 | 0.138 | 2.48E-39 | 1.25E-38 | 273 | 150 |
| Tepidiphilus | 1.918 | 0.125 | 7.42E-39 | 3.63E-38 | 273 | 126 |
| Family_Oscillospiraceae | -2.567 | 0.168 | 1.62E-38 | 7.71E-38 | 273 | 97 |
| Roseburia | -2.502 | 0.165 | 5.21E-38 | 2.41E-37 | 273 | 89 |
| Haemophilus | 2.285 | 0.154 | 6.62E-37 | 2.98E-36 | 273 | 165 |
| Muribaculaceae | 2.570 | 0.174 | 1.58E-36 | 6.93E-36 | 273 | 151 |
| X.Ruminococcus._gnavus_group | -2.977 | 0.202 | 1.87E-36 | 8.00E-36 | 273 | 73 |
| Fusobacterium | 1.977 | 0.135 | 3.09E-36 | 1.29E-35 | 273 | 122 |
| Chryseobacterium | 2.325 | 0.164 | 1.28E-34 | 5.22E-34 | 273 | 123 |
| Massilia | 2.176 | 0.154 | 1.86E-34 | 7.38E-34 | 273 | 127 |
| UBA1819 | -1.562 | 0.111 | 4.45E-34 | 1.73E-33 | 273 | 69 |
| Methylobacterium.Methylorubrum | 2.269 | 0.165 | 6.29E-33 | 2.39E-32 | 273 | 123 |
| Christensenellaceae_R.7_group | -2.245 | 0.165 | 1.83E-32 | 6.82E-32 | 273 | 78 |
| Bradyrhizobium | 1.767 | 0.131 | 3.78E-32 | 1.37E-31 | 273 | 104 |
| Lachnospiraceae_FCS020_group | -1.690 | 0.125 | 3.97E-32 | 1.42E-31 | 273 | 66 |
| Brevibacterium | 1.760 | 0.130 | 4.37E-32 | 1.53E-31 | 273 | 110 |
| Hydrogenophaga | 2.068 | 0.157 | 6.15E-31 | 2.10E-30 | 273 | 103 |
| Ochrobactrum | 1.971 | 0.150 | 6.90E-31 | 2.31E-30 | 273 | 104 |
| Erysipelatoclostridium | -1.983 | 0.152 | 2.08E-30 | 6.86E-30 | 273 | 79 |
| Family_Neisseriaceae | 1.721 | 0.134 | 7.35E-30 | 2.37E-29 | 273 | 90 |
| Cutibacterium | 0.967 | 0.075 | 9.05E-30 | 2.87E-29 | 273 | 53 |
| Anaerobacillus | 1.418 | 0.112 | 4.23E-29 | 1.31E-28 | 273 | 98 |
| Acidocella | 1.320 | 0.105 | 6.24E-29 | 1.90E-28 | 273 | 78 |
| Bdellovibrio | 1.698 | 0.136 | 1.42E-28 | 4.26E-28 | 273 | 92 |
| Sphingopyxis | 1.701 | 0.137 | 3.01E-28 | 8.86E-28 | 273 | 97 |
| X.Clostridium._innocuum_group | -1.274 | 0.104 | 8.72E-28 | 2.53E-27 | 273 | 54 |
| Family_Morganellaceae | 1.130 | 0.093 | 2.74E-27 | 7.80E-27 | 273 | 58 |
| Cryobacterium | 1.407 | 0.117 | 4.13E-27 | 1.16E-26 | 273 | 89 |
| Adlercreutzia | -1.603 | 0.134 | 1.03E-26 | 2.84E-26 | 273 | 57 |
| Achromobacter | 1.110 | 0.093 | 1.06E-26 | 2.87E-26 | 273 | 60 |
| Dubosiella | 1.235 | 0.104 | 1.57E-26 | 4.20E-26 | 273 | 68 |
| Pseudoclavibacter | 1.098 | 0.093 | 4.16E-26 | 1.10E-25 | 273 | 66 |
| Gordonia | 1.455 | 0.125 | 8.49E-26 | 2.20E-25 | 273 | 81 |
| Planococcus | 1.200 | 0.104 | 1.83E-25 | 4.66E-25 | 273 | 67 |
| Bosea | 1.513 | 0.131 | 2.35E-25 | 5.92E-25 | 273 | 77 |
| Faecalibaculum | 1.219 | 0.106 | 2.45E-25 | 6.08E-25 | 273 | 61 |
| Erysipelotrichaceae_UCG.003 | -2.466 | 0.215 | 4.92E-25 | 1.20E-24 | 273 | 56 |
| Roseomonas | 1.303 | 0.114 | 5.60E-25 | 1.35E-24 | 273 | 71 |
| Kocuria | 1.661 | 0.147 | 1.73E-24 | 4.11E-24 | 273 | 87 |
| Mycobacterium | 1.308 | 0.118 | 1.19E-23 | 2.79E-23 | 273 | 66 |
| Jeotgalicoccus | 0.999 | 0.092 | 3.18E-23 | 7.35E-23 | 273 | 52 |
| X.Eubacterium._ventriosum_group | -1.518 | 0.143 | 2.85E-22 | 6.49E-22 | 273 | 53 |
| Pseudoxanthomonas | 1.430 | 0.137 | 1.02E-21 | 2.29E-21 | 273 | 59 |
| Family_Rhizobiaceae | 1.067 | 0.102 | 1.05E-21 | 2.34E-21 | 273 | 47 |
| Knoellia | 1.208 | 0.116 | 1.14E-21 | 2.49E-21 | 273 | 63 |
| Intestinibacter | -1.821 | 0.174 | 1.17E-21 | 2.54E-21 | 273 | 54 |
| Brochothrix | 1.142 | 0.111 | 3.84E-21 | 8.21E-21 | 273 | 50 |
| Yersinia | 1.101 | 0.107 | 4.23E-21 | 8.93E-21 | 273 | 48 |
| Aquabacterium | 1.691 | 0.165 | 4.30E-21 | 8.97E-21 | 273 | 74 |
| Family_Comamonadaceae | 1.464 | 0.143 | 5.01E-21 | 1.03E-20 | 273 | 73 |
| Vulcaniibacterium | 1.052 | 0.103 | 6.09E-21 | 1.24E-20 | 273 | 50 |
| Thermicanus | 1.094 | 0.108 | 9.36E-21 | 1.88E-20 | 273 | 48 |
| Sphingobium | 1.246 | 0.124 | 1.78E-20 | 3.54E-20 | 273 | 60 |
| Family_Ruminococcaceae | -1.182 | 0.118 | 2.55E-20 | 5.01E-20 | 273 | 62 |
| Paracoccus | 1.415 | 0.142 | 3.23E-20 | 6.28E-20 | 273 | 63 |
| Arthrobacter | 0.817 | 0.082 | 4.10E-20 | 7.87E-20 | 273 | 24 |
| Caulobacter | 1.174 | 0.119 | 6.46E-20 | 1.23E-19 | 273 | 53 |
| CAG.352 | -2.517 | 0.264 | 8.46E-19 | 1.59E-18 | 273 | 67 |
| Cellulomonas | 1.160 | 0.122 | 9.30E-19 | 1.73E-18 | 273 | 52 |
| Romboutsia | -2.043 | 0.216 | 1.70E-18 | 3.13E-18 | 273 | 83 |
| Atopostipes | 0.956 | 0.101 | 1.93E-18 | 3.51E-18 | 273 | 34 |
| Bifidobacterium | -2.375 | 0.257 | 7.16E-18 | 1.29E-17 | 273 | 151 |
| Blastococcus | 1.141 | 0.125 | 1.48E-17 | 2.63E-17 | 273 | 50 |
| Noviherbaspirillum | 1.037 | 0.115 | 4.10E-17 | 7.23E-17 | 273 | 37 |
| Rhodococcus | 1.134 | 0.126 | 4.43E-17 | 7.74E-17 | 273 | 41 |
| Brevundimonas | 0.993 | 0.111 | 7.09E-17 | 1.22E-16 | 273 | 35 |
| Pseudoalteromonas | 0.824 | 0.093 | 9.74E-17 | 1.66E-16 | 273 | 31 |
| Cloacibacterium | 1.341 | 0.151 | 1.05E-16 | 1.77E-16 | 273 | 48 |
| Nocardioides | 1.114 | 0.127 | 1.69E-16 | 2.84E-16 | 273 | 43 |
| Aeromonas | 0.938 | 0.107 | 2.03E-16 | 3.37E-16 | 273 | 34 |
| Modestobacter | 1.120 | 0.129 | 4.15E-16 | 6.82E-16 | 273 | 39 |
| Staphylococcus | 1.148 | 0.133 | 5.02E-16 | 8.17E-16 | 273 | 39 |
| Lachnospiraceae_ND3007_group | -1.447 | 0.168 | 5.28E-16 | 8.52E-16 | 273 | 53 |
| Clostridium_sensu_stricto_1 | -1.664 | 0.193 | 6.27E-16 | 1.00E-15 | 273 | 71 |
| Anaerococcus | 1.255 | 0.147 | 8.46E-16 | 1.34E-15 | 273 | 64 |
| Marvinbryantia | -1.051 | 0.125 | 2.57E-15 | 4.03E-15 | 273 | 43 |
| Aerococcus | 1.024 | 0.124 | 8.06E-15 | 1.25E-14 | 273 | 32 |
| UCG.002 | -1.476 | 0.180 | 9.14E-15 | 1.41E-14 | 273 | 68 |
| Leptotrichia | 0.998 | 0.122 | 1.08E-14 | 1.64E-14 | 273 | 30 |
| Gardnerella | 0.963 | 0.118 | 1.56E-14 | 2.35E-14 | 273 | 28 |
| Abiotrophia | 0.865 | 0.108 | 3.80E-14 | 5.70E-14 | 273 | 25 |
| Psychrobacter | 1.026 | 0.128 | 4.01E-14 | 5.97E-14 | 273 | 32 |
| Sandaracinobacter | 0.959 | 0.121 | 5.29E-14 | 7.79E-14 | 273 | 27 |
| NK4A214_group | -1.226 | 0.160 | 2.85E-13 | 4.17E-13 | 273 | 46 |
| Peptoniphilus | 0.942 | 0.129 | 3.32E-12 | 4.81E-12 | 273 | 49 |
| Lactobacillus | 1.568 | 0.217 | 4.82E-12 | 6.87E-12 | 273 | 124 |
| Gordonibacter | -0.748 | 0.103 | 4.80E-12 | 6.87E-12 | 273 | 40 |
| Atopobium | 0.893 | 0.125 | 7.61E-12 | 1.08E-11 | 273 | 61 |
| Ruminococcus | -1.335 | 0.189 | 1.24E-11 | 1.74E-11 | 273 | 58 |
| DTU089 | -0.649 | 0.093 | 1.88E-11 | 2.61E-11 | 273 | 42 |
| Finegoldia | 0.941 | 0.137 | 4.41E-11 | 6.08E-11 | 273 | 52 |
| Dialister | -1.641 | 0.246 | 1.50E-10 | 2.06E-10 | 273 | 56 |
| Faecalitalea | -1.048 | 0.158 | 1.96E-10 | 2.66E-10 | 273 | 39 |
| Rothia | 1.080 | 0.164 | 2.43E-10 | 3.27E-10 | 273 | 79 |
| CAG.56 | -1.100 | 0.168 | 2.79E-10 | 3.72E-10 | 273 | 39 |
| X.Eubacterium._brachy_group | -0.643 | 0.104 | 2.34E-09 | 3.11E-09 | 273 | 39 |
| Negativibacillus | -0.685 | 0.116 | 1.09E-08 | 1.43E-08 | 273 | 38 |
| Ligilactobacillus | 0.777 | 0.136 | 2.69E-08 | 3.51E-08 | 273 | 68 |
| Gemella | 0.858 | 0.152 | 3.93E-08 | 5.09E-08 | 273 | 70 |
| Escherichia.Shigella | -1.354 | 0.241 | 4.70E-08 | 6.05E-08 | 273 | 110 |
| Akkermansia | -1.149 | 0.217 | 2.54E-07 | 3.24E-07 | 273 | 63 |
| Streptococcus | -0.975 | 0.195 | 1.00E-06 | 1.27E-06 | 273 | 259 |
| Candidatus_Soleaferrea | -0.363 | 0.076 | 3.20E-06 | 4.02E-06 | 273 | 35 |
| UCG.005 | -0.669 | 0.142 | 4.00E-06 | 4.99E-06 | 273 | 46 |
| Sellimonas | -0.657 | 0.140 | 4.36E-06 | 5.40E-06 | 273 | 31 |
| Family_Coriobacteriales_Incertae_Sedis | -0.472 | 0.106 | 1.22E-05 | 1.50E-05 | 273 | 28 |
| Enterorhabdus | -0.531 | 0.120 | 1.50E-05 | 1.83E-05 | 273 | 41 |
| Holdemanella | -0.828 | 0.206 | 7.65E-05 | 9.27E-05 | 273 | 32 |
| X.Eubacterium._eligens_group | -0.551 | 0.138 | 8.02E-05 | 9.66E-05 | 273 | 30 |
| Bacteroides | -1.020 | 0.257 | 9.35E-05 | 1.12E-04 | 273 | 165 |
| Lachnospiraceae_NK4A136_group | -0.893 | 0.226 | 1.01E-04 | 1.20E-04 | 273 | 130 |
| Prevotella_9 | 0.706 | 0.189 | 2.33E-04 | 2.75E-04 | 273 | 44 |
| Tyzzerella | -0.471 | 0.135 | 5.88E-04 | 6.88E-04 | 273 | 28 |
| Alistipes | -0.590 | 0.176 | 8.84E-04 | 1.03E-03 | 273 | 66 |
| Phascolarctobacterium | -0.417 | 0.124 | 9.19E-04 | 1.06E-03 | 273 | 27 |
| Colidextribacter | -0.376 | 0.122 | 2.36E-03 | 2.71E-03 | 273 | 53 |
| Holdemania | -0.231 | 0.081 | 4.54E-03 | 5.18E-03 | 273 | 30 |
| Actinomyces | 0.514 | 0.180 | 4.72E-03 | 5.35E-03 | 273 | 177 |
| Lachnospiraceae_UCG.001 | -0.353 | 0.133 | 8.35E-03 | 9.39E-03 | 273 | 39 |
| Terrisporobacter | -0.315 | 0.125 | 1.23E-02 | 1.38E-02 | 273 | 24 |
| Lachnospira | -0.326 | 0.130 | 1.30E-02 | 1.44E-02 | 273 | 44 |
| Family_XIII_UCG.001 | -0.204 | 0.087 | 1.99E-02 | 2.19E-02 | 273 | 33 |
| Enterococcus | 0.358 | 0.155 | 2.19E-02 | 2.40E-02 | 273 | 52 |
| Moryella | -0.144 | 0.069 | 3.84E-02 | 4.18E-02 | 273 | 23 |

**Table S17:** Bray-Curtis diversity ADONIS results indicate a significant effect of **MASLD** and MASH on sample diversity in stool (ADONIS: p<0.05, R2 = 0.02 and R2=0.03, respectively)

|  | | | | | | |
| --- | --- | --- | --- | --- | --- | --- |
|  |  |  |  |  |  |  |
|  |  |  |  |  |  |  |
|  |  |  |  |  |  |  |
|  |  |  |  |  |  |  |
|  | | | | | | |
|  |  |  |  |  |  |  |
|  |  |  |  |  |  |  |
|  |  |  |  |  |  |  |
|  |  |  |  |  |  |  |
|  | | | | | | |
|  |  |  |  |  |  |  |
|  |  |  |  |  |  |  |
|  |  |  |  |  |  |  |
|  |  |  |  |  |  |  |
|  | | | | | | |
|  |  |  |  |  |  |  |
|  |  |  |  |  |  |  |
|  |  |  |  |  |  |  |
|  |  |  |  |  |  |  |

| **ADONIS NLO vs MASLD Stool Unscaled** | | | | | | |
| --- | --- | --- | --- | --- | --- | --- |
|  | Df | Sum of Squares | F Model | R2 | p-value | p-adjusted |
| HbA1c | 1 | 0.34 | 1.654 | 0.02 | 0.033 | 0.3764 |
| MASLD | 1 | 0.261 | 1.271 | 0.01 | 0.139 | 0.7927 |
| Sex | 1 | 0.202 | 0.982 | 0.01 | 0.448 | 1 |
| Age | 1 | 0.158 | 0.77 | 0.01 | 0.836 | 1 |
| HbA1c * MASLD | 1 | 0.189 | 0.919 | 0.01 | 0.539 | 1 |
| Residual | 85 | 17.452 | NA | 0.94 | NA | NA |
| Total | 90 | 18.601 | NA | 1 | NA | NA |
| **ADONIS NLO vs MASH Stool Unscaled** | | | | | | |
|  | Df | Sum of Squares | F Model | R2 | p-value | p-adjusted |
| HbA1c | 1 | 0.322 | 1.618 | 0.03 | 0.037 | 0.3422 |
| MASH | 1 | 0.287 | 1.444 | 0.03 | 0.06 | 0.3422 |
| Sex | 1 | 0.243 | 1.222 | 0.02 | 0.154 | 0.5855 |
| Age | 1 | 0.174 | 0.877 | 0.02 | 0.621 | 1 |
| HbA1c * MASH | 1 | 0.207 | 1.041 | 0.02 | 0.371 | 1 |
| Residual | 49 | 9.744 | NA | 0.89 | NA | NA |
| Total | 54 | 10.977 | NA | 1 | NA | NA |
| **ADONIS F0 with MASLD vs F1-F2-F3-F4 with MASLD Stool Unscaled** | | | | | | |
|  | Df | Sum of Squares | F Model | R2 | p- value | p-adjusted |
| HbA1c | 1 | 0.278 | 1.362 | 0.02 | 0.092 | 0.5246 |
| F1-F2-F3-F4 | 1 | 0.306 | 1.503 | 0.02 | 0.068 | 0.5246 |
| Sex | 1 | 0.167 | 0.822 | 0.01 | 0.728 | 1 |
| Age | 1 | 0.176 | 0.863 | 0.01 | 0.629 | 1 |
| HbA1c * F1-F2-F3-F4 | 1 | 0.201 | 0.988 | 0.01 | 0.424 | 1 |
| Residual | 63 | 12.836 | NA | 0.92 | NA | NA |
| Total | 68 | 13.965 | NA | 1 | NA | NA |
| **ADONIS F0 with MASLD vs F3-F4 with MASLD Stool Unscaled** | | | | | | |
|  | Df | Sum of Squares | F Model | R2 | p-value | p-adjusted |
| HbA1c | 1 | 0.214 | 1 | 0.03 | 0.373 | 1 |
| F3-F4 | 1 | 0.238 | 1.114 | 0.03 | 0.272 | 1 |
| Sex | 1 | 0.24 | 1.124 | 0.03 | 0.28 | 1 |
| Age | 1 | 0.148 | 0.692 | 0.02 | 0.923 | 1 |
| HbA1c * F3-F4 | 1 | 0.235 | 1.099 | 0.03 | 0.291 | 1 |
| Residual | 30 | 6.405 | NA | 0.86 | NA | NA |
| Total | 35 | 7.479 | NA | 1 | NA | NA |

**Table S18:** Bray-Curtis diversity ADONIS results indicate tissue type as well as all groups of interest (MASLD, MASH, presence, and severe fibrosis) all had a significant impact on beta diversity (ADONIS: p<0.05)

|  | | | | | | |
| --- | --- | --- | --- | --- | --- | --- |
|  |  |  |  |  |  |  |
|  |  |  |  |  |  |  |
|  |  |  |  |  |  |  |
|  |  |  |  |  |  |  |
|  |  |  |  |  |  |  |
|  |  |  |  |  |  |  |
|  |  |  |  |  |  |  |
|  | | | | | | |
|  |  |  |  |  |  |  |
|  |  |  |  |  |  |  |
|  |  |  |  |  |  |  |
|  |  |  |  |  |  |  |
|  |  |  |  |  |  |  |
|  |  |  |  |  |  |  |
|  |  |  |  |  |  |  |
|  | | | | | | |
|  |  |  |  |  |  |  |
|  |  |  |  |  |  |  |
|  |  |  |  |  |  |  |
|  |  |  |  |  |  |  |
|  |  |  |  |  |  |  |
|  |  |  |  |  |  |  |
|  | | | | | | |
|  |  |  |  |  |  |  |
|  |  |  |  |  |  |  |
|  |  |  |  |  |  |  |
|  |  |  |  |  |  |  |
|  |  |  |  |  |  |  |
|  |  |  |  |  |  |  |

| **ADONIS NLO vs MASLD Tissue Unscaled** | | | | | | |
| --- | --- | --- | --- | --- | --- | --- |
|  | Df | Sum of Squares | F Model | R2 | p-value | p-adjusted |
| Sample Type | 1 | 0.786 | 3.537 | 0.02 | 0.001 | **0.0085** |
| HbA1c | 1 | 0.338 | 1.522 | 0.01 | 0.008 | **0.0339** |
| MASLD | 1 | 0.661 | 2.976 | 0.01 | 0.001 | **0.0085** |
| Sex | 1 | 0.253 | 1.137 | 0.01 | 0.194 | 0.5483 |
| Age | 1 | 0.354 | 1.594 | 0.01 | 0.007 | **0.0339** |
| SubjectID | 86 | 22.087 | 1.156 | 0.49 | 0.001 | **0.0085** |
| Sample Type * HbA1c | 1 | 0.276 | 1.241 | 0.01 | 0.078 | 0.248 |
| Sample Type * MASLD | 1 | 0.35 | 1.576 | 0.01 | 0.007 | **0.0339** |
| Sample Type * MASLD * HbA1c | 1 | 0.334 | 1.503 | 0.01 | 0.01 | **0.0363** |
| Residual | 87 | 19.33 | NA | 0.43 | NA | NA |
| Total | 181 | 44.769 | NA | 1 | NA | NA |
| **ADONIS NLO vs MASH Tissue Unscaled** | | | | | | |
|  | Df | Sum of Squares | F Model | R2 | p-value | p-adjusted |
| Sample Type | 1 | 0.568 | 2.657 | 0.02 | 0.001 | **0.0085** |
| HbA1c | 1 | 0.4 | 1.869 | 0.02 | 0.001 | **0.0085** |
| MASH | 1 | 0.574 | 2.685 | 0.02 | 0.001 | **0.0085** |
| Sex | 1 | 0.258 | 1.207 | 0.01 | 0.128 | 0.3617 |
| Age | 1 | 0.394 | 1.843 | 0.02 | 0.003 | **0.0153** |
| SubjectID | 50 | 12.152 | 1.136 | 0.46 | 0.002 | **0.0127** |
| Sample Type * HbA1c | 1 | 0.298 | 1.391 | 0.01 | 0.038 | 0.1208 |
| Sample Type * MASH | 1 | 0.329 | 1.537 | 0.01 | 0.009 | **0.0327** |
| Sample Type * MASH * HbA1c | 1 | 0.35 | 1.636 | 0.01 | 0.004 | **0.017** |
| Residual | 51 | 10.908 | NA | 0.42 | NA | NA |
| Total | 109 | 26.231 | NA | 1 | NA | NA |
| **ADONIS F0 with MASLD vs F1-F2-F3-F4 with MASLD Tissue Unscaled** | | | | | | |
|  | Df | Sum of Squares | F Model | R2 | p- value | p- adjusted |
| Sample Type | 1 | 0.619 | 2.5 | 0.02 | 0.001 | **0.0127** |
| HbA1c | 1 | 0.275 | 1.11 | 0.01 | 0.223 | 1 |
| F1-F2-F3-F4 | 1 | 0.678 | 2.74 | 0.02 | 0.001 | **0.0127** |
| Sex | 1 | 0.292 | 1.178 | 0.01 | 0.135 | 0.8584 |
| Age | 1 | 0.313 | 1.266 | 0.01 | 0.058 | 0.4917 |
| Sample Type * HbA1c | 1 | 0.257 | 1.038 | 0.01 | 0.35 | 1 |
| Sample Type * F1-F2-F3-F4 | 1 | 0.249 | 1.006 | 0.01 | 0.427 | 1 |
| HbA1c * F1-F2-F3-F4 | 1 | 0.269 | 1.089 | 0.01 | 0.254 | 1 |
| Sample Type * F1-F2-F3-F4 * HbA1c | 1 | 0.211 | 0.852 | 0.01 | 0.82 | 1 |
| Residual | 128 | 31.671 | NA | 0.91 | NA | NA |
| Total | 137 | 34.833 | NA | 1 | NA | NA |
| **ADONIS F0 with MASLD vs F3-F4 with MASLD Tissue Unscaled** | | | | | | |
|  | Df | Sum of Squares | F Model | R2 | p-value | p-adjusted |
| Sample Type | 1 | 0.402 | 1.681 | 0.02 | 0.002 | **0.0254** |
| HbA1c | 1 | 0.287 | 1.201 | 0.02 | 0.127 | 0.6613 |
| F3-F4 | 1 | 0.596 | 2.493 | 0.03 | 0.001 | **0.0254** |
| Sex | 1 | 0.297 | 1.243 | 0.02 | 0.08 | 0.6613 |
| Age | 1 | 0.29 | 1.211 | 0.02 | 0.13 | 0.6613 |
| Sample Type * HbA1c | 1 | 0.262 | 1.095 | 0.01 | 0.272 | 0.9883 |
| Sample Type * F3-F4 | 1 | 0.254 | 1.062 | 0.01 | 0.331 | 1 |
| HbA1c * F3-F4 | 1 | 0.281 | 1.176 | 0.02 | 0.159 | 0.674 |
| Sample Type * F3-F4 * HbA1c | 1 | 0.194 | 0.811 | 0.01 | 0.86 | 1 |
| Residual | 62 | 14.823 | NA | 0.84 | NA | NA |
| Total | 71 | 17.687 | NA | 1 | NA | NA |

**Table S19:** Bray-Curtis diversity ADONIS results indicate tissue type as well as all groups of interest (MASLD, MASH, presence, and severe fibrosis) all had a significant impact on beta diversity (ADONIS: p<0.05) in scaled estimated ASV abundances

| **ADONIS NLO vs MASLD Tissue Scaled** | | | | | | |
| --- | --- | --- | --- | --- | --- | --- |
|  | Df | Sum of Squares | F Model | R2 | p-value | p-adjusted |
| Sample Type | 1 | 0.925 | 5.708 | 0.03 | 0.001 | **0.0085** |
| HbA1c | 1 | 0.28 | 1.727 | 0.01 | 0.007 | **0.0445** |
| MASLD | 1 | 0.556 | 3.43 | 0.02 | 0.001 | **0.0085** |
| Sex | 1 | 0.231 | 1.426 | 0.01 | 0.036 | 0.1308 |
| Age | 1 | 0.243 | 1.498 | 0.01 | 0.027 | 0.1145 |
| SubjectID | 82 | 15.274 | 1.149 | 0.49 | 0.001 | **0.0085** |
| Sample Type * HbA1c | 1 | 0.194 | 1.194 | 0.01 | 0.145 | 0.461 |
| Sample Type * MASLD | 1 | 0.269 | 1.659 | 0.01 | 0.01 | 0.0509 |
| Sample Type * MASLD * HbA1c | 1 | 0.162 | 1.002 | 0.01 | 0.447 | 1 |
| Residual | 81 | 13.128 | NA | 0.42 | NA | NA |
| Total | 171 | 31.262 | NA | 1 | NA | NA |
| **ADONIS NLO vs MASH Tissue Scaled** | | | | | | |
|  | Df | Sum of Squares | F Model | R2 | p- value | p- adjusted |
| Sample Type | 1 | 0.611 | 4.061 | 0.03 | 0.001 | **0.0085** |
| HbA1c | 1 | 0.308 | 2.045 | 0.02 | 0.001 | **0.0085** |
| MASH | 1 | 0.524 | 3.482 | 0.03 | 0.001 | **0.0085** |
| Sex | 1 | 0.218 | 1.448 | 0.01 | 0.04 | 0.1453 |
| Age | 1 | 0.24 | 1.595 | 0.01 | 0.013 | 0.0551 |
| SubjectID | 47 | 8.028 | 1.135 | 0.46 | 0.004 | **0.0203** |
| Sample Type * HbA1c | 1 | 0.198 | 1.315 | 0.01 | 0.096 | 0.3052 |
| Sample Type * MASH | 1 | 0.299 | 1.986 | 0.02 | 0.002 | **0.0127** |
| Sample Type * MASH * HbA1c | 1 | 0.179 | 1.187 | 0.01 | 0.248 | 0.7009 |
| Residual | 46 | 6.919 | NA | 0.39 | NA | NA |
| Total | 101 | 17.522 | NA | 1 | NA | NA |
| **ADONIS F0 with MASLD vs F1-F2-F3-F4 with MASLD Tissue Scaled** | | | | | | |
|  | Df | Sum of Squares | F Model | R2 | p-value | p-adjusted |
| Sample Type | 1 | 0.625 | 3.616 | 0.03 | 0.001 | **0.0085** |
| HbA1c | 1 | 0.231 | 1.337 | 0.01 | 0.061 | 0.3103 |
| F1-F2-F3-F4 | 1 | 0.581 | 3.356 | 0.02 | 0.001 | **0.0085** |
| Sex | 1 | 0.244 | 1.41 | 0.01 | 0.032 | 0.2035 |
| Age | 1 | 0.203 | 1.171 | 0.01 | 0.179 | 0.7588 |
| SubjectID | 61 | 11.793 | 1.118 | 0.48 | 0.001 | **0.0085** |
| Sample Type * HbA1c | 1 | 0.163 | 0.94 | 0.01 | 0.571 | 1 |
| Sample Type * F1-F2-F3-F4 | 1 | 0.154 | 0.891 | 0.01 | 0.699 | 1 |
| Sample Type * F1-F2-F3-F4 * HbA1c | 1 | 0.149 | 0.863 | 0.01 | 0.72 | 1 |
| Residual | 60 | 10.378 | NA | 0.42 | NA | NA |
| Total | 129 | 24.521 | NA | 1 | NA | NA |
| **ADONIS F0 with MASLD vs F3-F4 with MASLD Tissue Scaled** | | | | | | |
|  | Df | Sum of Squares | F Model | R2 | p-value | p-adjusted |
| Sample Type | 1 | 0.389 | 2.235 | 0.03 | 0.001 | **0.0127** |
| HbA1c | 1 | 0.25 | 1.434 | 0.02 | 0.039 | 0.3307 |
| F3-F4 | 1 | 0.631 | 3.628 | 0.05 | 0.001 | **0.0127** |
| Sex | 1 | 0.235 | 1.349 | 0.02 | 0.067 | 0.351 |
| Age | 1 | 0.237 | 1.36 | 0.02 | 0.069 | 0.351 |
| SubjectID | 31 | 5.685 | 1.053 | 0.43 | 0.147 | 0.6232 |
| Sample Type * HbA1c | 1 | 0.167 | 0.96 | 0.01 | 0.535 | 1 |
| Sample Type * F3-F4 | 1 | 0.159 | 0.912 | 0.01 | 0.619 | 1 |
| Sample Type * F3-F4 * HbA1c | 1 | 0.128 | 0.737 | 0.01 | 0.883 | 1 |
| Residual | 31 | 5.396 | NA | 0.41 | NA | NA |
| Total | 70 | 13.276 | NA | 1 | NA | NA |

**Table 20:** Chao1 alpha diversity differs significantly between liver NLO and MASLD and liver NLO and MASH

|  |  |  |  |  |
| --- | --- | --- | --- | --- |
|  |  |  |  |  |
|  |  |  |  |  |
|  |  |  |  |  |
|  |  |  |  |  |
|  |  |  |  |  |
|  |  |  |  |  |
|  |  |  |  |  |
|  |  |  |  |  |
|  |  |  |  |  |

| **Adipose** | **Sum Sq** | **Df** | **F value** | **p value** |
| --- | --- | --- | --- | --- |
|  |  |  |  |  |
| MASLD | 115 | 1 | 0 | 0.74 |
| HbA1c | 1821 | 1 | 2 | 0.18 |
| MASLD * HbA1c | 1650 | 1 | 2 | 0.21 |
| Residuals | 81133 | 80 | NA | NA |
|  |  |  |  |  |
| MASH | 0 | 1 | 0 | 1 |
| HbA1c | 2289 | 1 | 3 | 0.08 |
| MASH * HbA1c | 1290 | 1 | 2 | 0.19 |
| Residuals | 32289 | 45 | NA | NA |
|  |  |  |  |  |
| F1-F2-F3-F4 | 168 | 1 | 0 | 0.7 |
| HbA1c | 399 | 1 | 0 | 0.56 |
| F1-F2-F3-F4 * HbA1c | 235 | 1 | 0 | 0.65 |
| Residuals | 68451 | 60 | NA | NA |
|  |  |  |  |  |
| F3-F4 | 21 | 1 | 0 | 0.9 |
| HbA1c | 468 | 1 | 0 | 0.54 |
| F3-F4 * HbA1c | 467 | 1 | 0 | 0.54 |
| Residuals | 37961 | 31 | NA | NA |
| **Liver** | **Sum Sq** | **Df** | **F value** | **p value** |
|  |  |  |  |  |
| MASLD | 8913 | 1 | 6 | **0.02** |
| HbA1c | 284 | 1 | 0 | 0.66 |
| MASLD * HbA1c | 6016 | 1 | 4 | 0.05 |
| Residuals | 120296 | 82 | NA | NA |
|  |  |  |  |  |
| MASH | 11675 | 1 | 10 | **0** |
| HbA1c | 195 | 1 | 0 | 0.69 |
| MASH * HbA1c | 4369 | 1 | 4 | 0.06 |
| Residuals | 56387 | 47 | NA | NA |
|  |  |  |  |  |
| F1-F2-F3-F4 | 251 | 1 | 0 | 0.69 |
| HbA1c | 2286 | 1 | 1 | 0.24 |
| F1-F2-F3-F4 * HbA1c | 684 | 1 | 0 | 0.52 |
| Residuals | 97707 | 61 | NA | NA |
|  |  |  |  |  |
| F3-F4 | 500 | 1 | 0 | 0.57 |
| HbA1c | 324 | 1 | 0 | 0.65 |
| F3-F4 * HbA1c | 135 | 1 | 0 | 0.77 |
| Residuals | 49294 | 32 | NA | NA |

**Table S21:** Contribution values for each genera are listed by row for each identified Dirichlet Multinomial (DMN) enterotype, a larger value indicates a greater contribution

|  | **Enterotype1** | **Enterotype2** | **Enterotype3** |
| --- | --- | --- | --- |
| Genus:Family_Bacillaceae | 10.1 | 2.1 | 1.3 |
| Genus:Corynebacterium | 4.3 | 5.2 | 1.6 |
| Genus:Pseudomonas | 2.6 | 2.0 | 0.8 |
| Genus:Streptococcus | 1.7 | 1.7 | 0.9 |
| Genus:Halomonas | 1.5 | 1.3 | 0.7 |
| Genus:Bacillus | 1.4 | 1.3 | 0.7 |
| Genus:Sphingobacterium | 1.4 | 1.0 | 0.4 |
| Genus:Escherichia-Shigella | 0.9 | 0.9 | 0.1 |
| Genus:Cutibacterium | 0.9 | 0.8 | 0.2 |
| Genus:Family_Pasteurellaceae | 0.5 | 0.5 | 0.1 |
| Genus:Family_Sphingobacteriaceae | 0.5 | 0.2 | 0.1 |
| Genus:Micrococcus | 0.5 | 0.7 | 0.2 |
| Genus:Enhydrobacter | 0.4 | 0.3 | 0.1 |
| Genus:Neisseria | 0.4 | 0.4 | 0.1 |
| Genus:Gemella | 0.4 | 0.5 | 0.1 |
| Genus:Flavobacterium | 0.4 | 0.2 | 0.2 |
| Genus:Finegoldia | 0.3 | 0.4 | 0.1 |
| Genus:Lactobacillus | 0.3 | 0.3 | 0.0 |
| Genus:Thermicanus | 0.3 | 0.3 | 0.1 |
| Genus:Lawsonella | 0.3 | 0.2 | 0.1 |
| Genus:Methylobacterium-Methylorubrum | 0.1 | 0.2 | 0.0 |
| Genus:Blastococcus | 0.1 | 0.2 | 0.0 |

**Table S22.** Taxa Significantly Associated with MASLD, MASH, Fibrosis, or HbA1c identified using MaAsLin2 and Unscaled Relative Abundance Data

| **Adipose** | **feature** | **Enriched** | **coef** | **stderr** | **pval** | **qval** | **N** | **N.not.zero** |
| --- | --- | --- | --- | --- | --- | --- | --- | --- |
| **NLO vs MASH Unscaled** | |  |  |  |  |  |  |  |
|  | Family_Morganellaceae | MASH | 0.87 | 0.23 | 0.00 | 0.04 | 55 | 17 |
|  | Planococcus | MASH | 0.82 | 0.24 | 0.00 | 0.10 | 55 | 22 |
|  | CAG.352 | hba1c | 0.68 | 0.16 | 0.00 | 0.03 | 55 | 7 |
|  | Brachybacterium | hba1c | 0.42 | 0.10 | 0.00 | 0.03 | 55 | 6 |
|  | Comamonas | MASH | -0.69 | 0.20 | 0.00 | 0.08 | 55 | 7 |
|  | Gordonia | MASH | -1.38 | 0.29 | 0.00 | 0.01 | 55 | 16 |
|  | Bdellovibrio | MASH | -1.44 | 0.37 | 0.00 | 0.04 | 55 | 24 |
| **NLO vs MASLD Unscaled** | |  |  |  |  |  |  |  |
|  | Gordonia | MASLD | -1.04 | 0.25 | 0.00 | 0.03 | 91 | 26 |
| **F0 vs F3-F4 Unscaled** | |  |  |  |  |  |  |  |
|  | Gordonia | MASLD | -1.04 | 0.25 | 0.00 | 0.03 | 91 | 26 |
| **F0 vs F1-F2-F3-F4 Unscaled** | |  |  |  |  |  |  |  |
|  | CAG.352 | hba1c | 0.65 | 0.13 | 0.00 | 0.00 | 69 | 7 |
|  | Family_Neisseriaceae | F1plus | 1.14 | 0.25 | 0.00 | 0.00 | 69 | 31 |
| **Liver and Stool: No significant results** | | | | | | | | |

**Table S23.** Taxa Significantly Associated with MASLD, MASH, Fibrosis, or HbA1c identified using MaAsLin2 and Scaled Estimated Absolute Abundance Data

| **Adipose** | **feature** | **Enriched** | **coef** | **stderr** | **pval** | **qval** | **N** | **N.not.zero** |
| --- | --- | --- | --- | --- | --- | --- | --- | --- |
| NLO vs MASH Scaled | |  |  |  |  |  |  |  |
|  | Gordonia | MASH | -3.21 | 0.58 | 0.00 | 0.00 | 51.00 | 20 |
|  | Paracoccus | MASH | 3.26 | 0.64 | 0.00 | 0.00 | 51.00 | 20 |
|  | Family_Morganellaceae | MASH | 2.18 | 0.50 | 0.00 | 0.01 | 51.00 | 21 |
|  | Bdellovibrio | MASH | -2.80 | 0.76 | 0.00 | 0.04 | 51.00 | 28 |
|  | Capnocytophaga | MASH | -2.23 | 0.59 | 0.00 | 0.04 | 51.00 | 18 |
|  | Granulicatella | MASH | 1.64 | 0.42 | 0.00 | 0.04 | 51.00 | 44 |
|  | Carnobacterium | MASH | -1.97 | 0.53 | 0.00 | 0.04 | 51.00 | 17 |
| NLO vs MASLD Scaled | |  |  |  |  |  |  |  |
|  | Gordonia | MASLD | -2.10 | 0.53 | 0.00 | 0.07 | 86.00 | 31 |
| F0 vs F3-F4 Scaled | |  |  |  |  |  |  |  |
|  | Carnobacterium | F3F4 | -3.79 | 0.70 | 0.00 | 0.00 | 35.00 | 14 |
| F0 vs F1-F2-F3-F4 Scaled | |  |  |  |  |  |  |  |
|  | Pseudoclavibacter | F1plus | 2.80 | 0.55 | 0.00 | 0.00 | 65.00 | 20 |
|  | Brevibacterium | F1plus | 2.05 | 0.48 | 0.00 | 0.02 | 65.00 | 34 |
|  | Family_Neisseriaceae | F1plus | 2.19 | 0.55 | 0.00 | 0.02 | 65.00 | 39 |
|  | Pseudarthrobacter | F1plus | -2.24 | 0.56 | 0.00 | 0.02 | 65.00 | 17 |
|  | Roseomonas | F1plus | -2.16 | 0.62 | 0.00 | 0.08 | 65.00 | 20 |
|  |  |  |  |  |  |  |  |  |
| **Liver** | **feature** | **value** | **coef** | **stderr** | **pval** | **qval** | **N** | **N.not.zero** |
| NLO vs MASH Scaled | |  |  |  |  |  |  |  |
|  | Carnobacterium | MASH | -3.20 | 0.36 | 0.00 | 0.00 | 51.00 | 11 |
|  | Eikenella | MASH | -3.00 | 0.51 | 0.00 | 0.00 | 51.00 | 28 |
|  | Pseudoclavibacter | MASH | -3.13 | 0.56 | 0.00 | 0.00 | 51.00 | 23 |
|  | Planococcus | MASH | 3.02 | 0.56 | 0.00 | 0.00 | 51.00 | 9 |
|  | Capnocytophaga | MASH | -2.97 | 0.65 | 0.00 | 0.00 | 51.00 | 27 |
|  | Porphyromonas | MASH | 3.59 | 0.80 | 0.00 | 0.00 | 51.00 | 16 |
|  | Family_Neisseriaceae | MASH | -3.15 | 0.77 | 0.00 | 0.01 | 51.00 | 33 |
|  | Mycobacterium | hba1c | 1.46 | 0.41 | 0.00 | 0.05 | 51.00 | 21 |
|  | Enterococcus | MASH | 2.79 | 0.79 | 0.00 | 0.05 | 51.00 | 22 |
|  | Gordonia | MASH | -2.22 | 0.64 | 0.00 | 0.05 | 51.00 | 37 |
| NLO vs MASLD Scaled | |  |  |  |  |  |  |  |
|  | Pseudoclavibacter | MASLD | -3.25 | 0.54 | 0.00 | 0.00 | 86.00 | 35 |
|  | Porphyromonas | MASLD | 3.71 | 0.63 | 0.00 | 0.00 | 86.00 | 40 |
|  | Eikenella | MASLD | -2.29 | 0.52 | 0.00 | 0.00 | 86.00 | 52 |
|  | Capnocytophaga | MASLD | -2.38 | 0.59 | 0.00 | 0.01 | 86.00 | 45 |
|  | Order_Lactobacillales | MASLD | 2.17 | 0.53 | 0.00 | 0.01 | 86.00 | 11 |
|  | Family_Neisseriaceae | MASLD | -2.60 | 0.66 | 0.00 | 0.01 | 86.00 | 59 |
|  | Gordonia | MASLD | -2.39 | 0.64 | 0.00 | 0.02 | 86.00 | 58 |
|  | Carnobacterium | MASLD | -2.01 | 0.54 | 0.00 | 0.02 | 86.00 | 27 |
| F0 vs F3-F4 Scaled | |  |  |  |  |  |  |  |
|  | Capnocytophaga | F3F4 | -4.82 | 0.78 | 0.00 | 0.00 | 36.00 | 13 |
| F0 vs F1-F2-F3-F4 Scaled | |  |  |  |  |  |  |  |
|  | Pseudarthrobacter | F1plus | -2.93 | 0.54 | 0.00 | 0.00 | 65.00 | 13 |
|  | Carnobacterium | F1plus | -2.48 | 0.56 | 0.00 | 0.01 | 65.00 | 13 |
|  | Capnocytophaga | F1plus | -2.48 | 0.62 | 0.00 | 0.02 | 65.00 | 23 |

**Supplementary Figures:**

**Figure S1: Patient Flow Chart**

**A)**

**
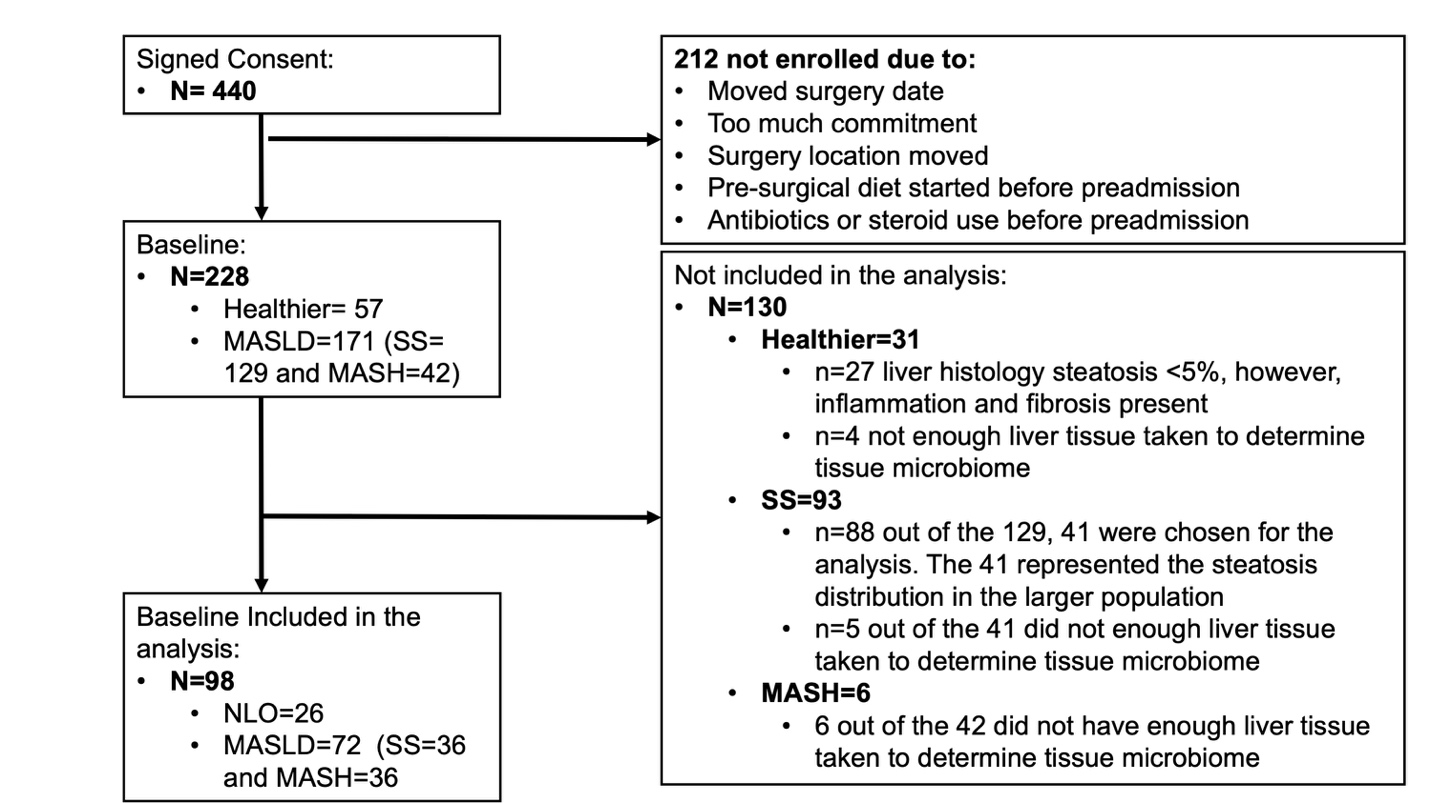
**

**B)**

**
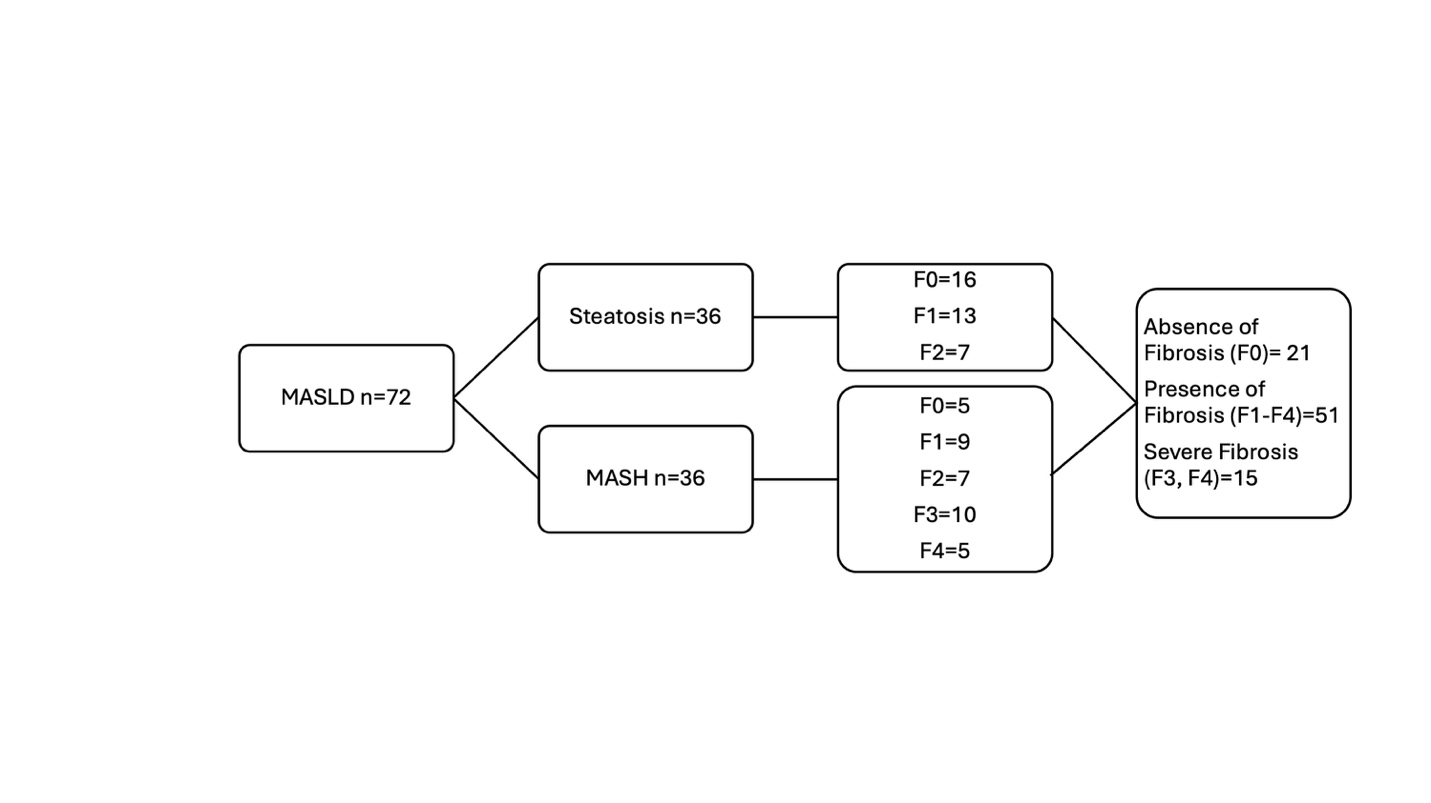
**

**Figure S2: Summary of Clinical and Biological Samples**


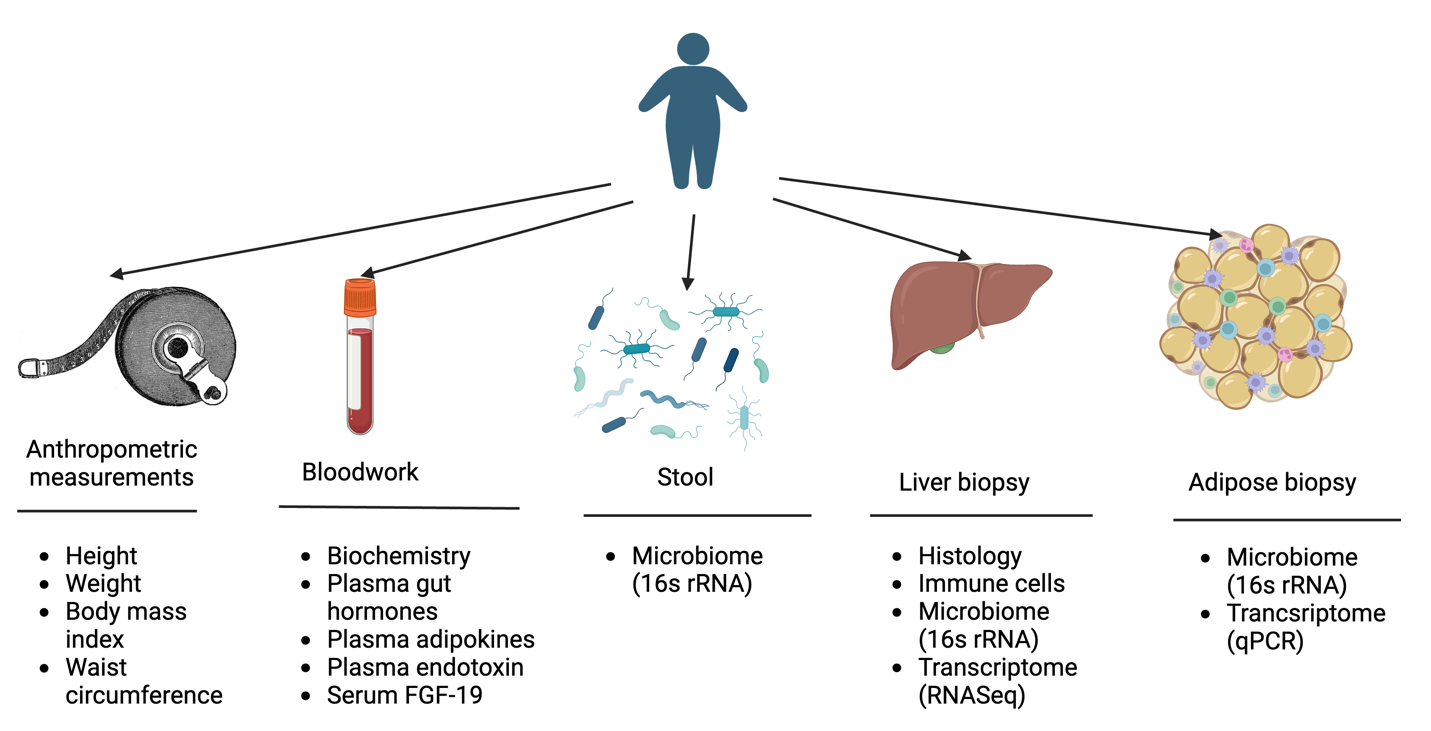


**Figure S3: Shared and Unique ASVs Between Stool, Adipose, and Liver Tissues.** Shared and unique Amplicon Sequence Variants (ASVs) shown in a standard venn diagrams to include all ASVs for (A) all samples, and filtered ASVs to include only those with a prevalence >10% and abundance >0.1% for (B) all samples, (C) NLO samples, (D) MASLD samples, and (E) MASH samples.

**Figure S4. Alpha Diversity Analysis of Rarefied and Non-Rarefied Data.** Scatter plots show the alpha diversity identified for (A) stool, (B) adipose, and (C) liver samples using both rarefied (x-axis) and non-rarefied (y axis) data. (D) Non-rarefied data was used to determine the alpha diversity of all sample types using the Observed, Chao1, and Shannon indices. (E) Rarefied data was used to determine the alpha diversity of stool samples using the Observed, Chao1, and Shannon indices.

**Figure S5: Bray-Curtis PCoA Plot of Stool, Adipose, and Liver Tissues.** PCoA plot of Bray-Curtis distances for (A-D) stool and (E-H) tissues. Data points are coloured by disease state (A, E) NLO and MASLD, and (B, F) NLO and MASH, and fibrosis level (C, G) F0 and F1, and (D, H) F0 and F3F4. Shape of point indicates HbA1c < 6 (triangle) or HbA1c >=6 (circle).

**Figure S6: MaAsLin2 Coefficient Values Between Disease States and Fibrosis Levels of Stool.** MaAsLin2 coef values of stool genera between disease states (A,E) NLO and MASLD, (B, F) NLO and MASH, and fibrosis levels (C,G) F0 and F1plus, and (D,H) F0 and F3F4. Fixed effects considered during the analysis were the factor of interest, as well as age, sex, and HbA1c. Included are genera the factor of interest and HbA1c (A-D), and genera just the factor of interest, but not HbA1c (E-H). Genera affected by age and sex are not shown.

**Figure S7: Relative Abundance (RA%) of Potential Translocated Taxa Identified in Stool, Adipose, and Liver Tissues.** The relative abundance (RA%) of potential translocated taxa in stool (green), adipose (blue), and liver (red) tissues, across all samples that contained these taxa. It can be observed that a number of patients contain these taxa of interest in all body sites of interest, while others do not.

**Figure S8: Scaled Estimated ASVs per Gram of Adipose and Liver Tissues:** The scaled estimated ASV abundance per gram of adipose and liver tissues for disease states (A) NLO and MASLD, (B) NLO and MASH, and fibrosis levels (C) F0 and F1plus, and (D) F0 and F3F4. A significant difference (p < 0.05) was identified between adipose and liver tissue estimated ASVs per gram of tissue, when (A) all samples were considered, but not when samples were removed from the analysis and only certain subgroups were considered (B-D). No significant difference was identified between the estimated ASVs per gram of tissue and the disease states or fibrosis levels, for either adipose or liver tissues.

**Figure S9: MaAsLin2 Coefficient Values Between Disease States and Fibrosis Levels of Adipose.** MaAsLin2 coef values >1 or <-1 of adipose genera between disease states (A,E) NLO and MASLD, (B, F) NLO and MASH, and fibrosis levels (C,G) F0 and F1plus, and (D,H) F0 and F3F4. Fixed effects considered during the analysis were the factor of interest, as well as age, sex, and HbA1c. Included are genera for (A-D) the factor of interest and HbA1c, and genera (E-H) influenced by the factor of interest, but not HbA1c. Age and sex had a smaller effect on a few select genera only, the results are not shown.

**Figure S10: MaAsLin2 Coefficient Values Between Disease States and Fibrosis Levels of Liver.** MaAsLin2 coef values >1 or <-1 of liver genera between disease states (A,E) NLO and MASLD, (B, F) NLO and MASH, and fibrosis levels (C,G) F0 and F1plus, and (D,H) F0 and F3F4. Fixed effects considered during the analysis were the factor of interest, as well as age, sex, and HbA1c. Included are genera for (A-D) the factor of interest and HbA1c, and genera (E-H) influenced by the factor of interest, but not HbA1c. Age and sex had a smaller effect on a few select genera only, the results are not shown.

**Figure S11: MaAsLin2 Coefficient Values Between Disease States and Fibrosis Levels of Adipose Estimated Genera Quantity.** MaAsLin2 coef values >1 or <-1 of adipose genera, scaled to estimate quantity, between disease states (A,E) NLO and MASLD, (B, F) NLO and MASH, and fibrosis levels (C,G) F0 and F1plus, and (D,H) F0 and F3F4. Fixed effects considered during the analysis were the factor of interest, as well as age, sex, and HbA1c. Included are genera for (A-D) the factor of interest and HbA1c, and genera (E-H) influenced by the factor of interest, but not HbA1c. Age and sex had a smaller effect on a few select genera only, the results are not shown.

**Figure S12: MaAsLin2 Coefficient Values Between Disease States and Fibrosis Levels of Liver Estimated Genera Quantity.** MaAsLin2 coef values of liver genera, scaled to estimate quantity, between disease states (A,E) NLO and MASLD, (B, F) NLO and MASH, and fibrosis levels (C,G) F0 and F1plus, and (D,H) F0 and F3F4. Fixed effects considered during the analysis were the factor of interest, as well as age, sex, and HbA1c. Included are genera for (A-D) the factor of interest and HbA1c, and genera (E-H) influenced by the factor of interest, but not HbA1c. Age and sex had a smaller effect on a few select genera only, the results are not shown.

**References:**

1 Schwenger, K. J. P. *et al.* Links between gut microbiome, metabolome, clinical variables and non-alcoholic fatty liver disease severity in bariatric patients. *Liver Int*, doi:10.1111/liv.15864 (2024).

2 NIH conference. Gastrointestinal surgery for severe obesity. Consensus Development Conference Panel. *Ann Intern Med* **115**, 956-961 (1991).

3 Matthews, D. R. *et al.* Homeostasis model assessment: insulin resistance and beta-cell function from fasting plasma glucose and insulin concentrations in man. *Diabetologia* **28**, 412-419, doi:10.1007/BF00280883 (1985).

4 Caporaso, J. G. *et al.* Ultra-high-throughput microbial community analysis on the Illumina HiSeq and MiSeq platforms. *ISME J* **6**, 1621-1624, doi:10.1038/ismej.2012.8 (2012).

5 Keshavjee, S. H. *et al.* Adipose Tissue and Plasma Markers Associated with HbA1c Pre- and Post-bariatric Surgery: a Cross-sectional and Cohort Study. *Obes Surg* **33**, 2443-2451, doi:10.1007/s11695-023-06679-z (2023).

6 Bolyen, E. *et al.* Reproducible, interactive, scalable and extensible microbiome data science using QIIME 2. *Nat Biotechnol* **37**, 852-857, doi:10.1038/s41587-019-0209-9 (2019).

7 S., A. *FastQC: a quality control tool for high throughput sequence data*, <<http://www.bioinformatics.babraham.ac.uk/projects/fastqc>> (2010).

8 Ewels, P., Magnusson, M., Lundin, S. & Kaller, M. MultiQC: summarize analysis results for multiple tools and samples in a single report. *Bioinformatics* **32**, 3047-3048, doi:10.1093/bioinformatics/btw354 (2016).

9 Martin, M. Cutadapt removes adapter sequences from high-throughput sequencing reads. *2011* **17**, 3, doi:10.14806/ej.17.1.200 (2011).

10 Rognes, T., Flouri, T., Nichols, B., Quince, C. & Mahe, F. VSEARCH: a versatile open source tool for metagenomics. *PeerJ* **4**, e2584, doi:10.7717/peerj.2584 (2016).

11 Edgar, R. C. Search and clustering orders of magnitude faster than BLAST. *Bioinformatics* **26**, 2460-2461, doi:10.1093/bioinformatics/btq461 (2010).

12 Bokulich, N. A. *et al.* Optimizing taxonomic classification of marker-gene amplicon sequences with QIIME 2's q2-feature-classifier plugin. *Microbiome* **6**, 90, doi:10.1186/s40168-018-0470-z (2018).

13 Kaehler, B. D. *et al.* Species abundance information improves sequence taxonomy classification accuracy. *Nat Commun* **10**, 4643, doi:10.1038/s41467-019-12669-6 (2019).

14 Janssen, S. *et al.* Phylogenetic Placement of Exact Amplicon Sequences Improves Associations with Clinical Information. *mSystems* **3**, doi:10.1128/mSystems.00021-18 (2018).

15 Ghorbani, Y. *et al.* Effect of faecal microbial transplant via colonoscopy in patients with severe obesity and insulin resistance: A randomized double-blind, placebo-controlled Phase 2 trial. *Diabetes Obes Metab* **25**, 479-490, doi:10.1111/dom.14891 (2023).

16 Goralska, J. *et al.* Enhanced GIP Secretion in Obesity Is Associated with Biochemical Alteration and miRNA Contribution to the Development of Liver Steatosis. *Nutrients* **12**, doi:10.3390/nu12020476 (2020).

17 Duan, Y. *et al.* Association of Inflammatory Cytokines With Non-Alcoholic Fatty Liver Disease. *Front Immunol* **13**, 880298, doi:10.3389/fimmu.2022.880298 (2022).

18 Han, Y. H., Choi, H., Kim, H. J. & Lee, M. O. Chemotactic cytokines secreted from Kupffer cells contribute to the sex-dependent susceptibility to non-alcoholic fatty liver diseases in mice. *Life Sci* **306**, 120846, doi:10.1016/j.lfs.2022.120846 (2022).

19 Fontes-Cal, T. C. M. *et al.* Crosstalk Between Plasma Cytokines, Inflammation, and Liver Damage as a New Strategy to Monitoring NAFLD Progression. *Front Immunol* **12**, 708959, doi:10.3389/fimmu.2021.708959 (2021).

20 El-Emshaty, H. M., Nasif, W. A. & Mohamed, I. E. Serum Cytokine of IL-10 and IL-12 in Chronic Liver Disease: The Immune and Inflammatory Response. *Dis Markers* **2015**, 707254, doi:10.1155/2015/707254 (2015).

21 Nagata, N., Chen, G., Xu, L. & Ando, H. An Update on the Chemokine System in the Development of NAFLD. *Medicina (Kaunas)* **58**, doi:10.3390/medicina58060761 (2022).

22 Roh, Y. S. & Seki, E. Chemokines and Chemokine Receptors in the Development of NAFLD. *Adv Exp Med Biol* **1061**, 45-53, doi:10.1007/978-981-10-8684-7_4 (2018).

23 Polyzos, S. A., Kountouras, J. & Mantzoros, C. S. Leptin in nonalcoholic fatty liver disease: a narrative review. *Metabolism* **64**, 60-78, doi:10.1016/j.metabol.2014.10.012 (2015).

24 Boutari, C., Perakakis, N. & Mantzoros, C. S. Association of Adipokines with Development and Progression of Nonalcoholic Fatty Liver Disease. *Endocrinol Metab (Seoul)* **33**, 33-43, doi:10.3803/EnM.2018.33.1.33 (2018).

25 Gatselis, N. K., Ntaios, G., Makaritsis, K. & Dalekos, G. N. Adiponectin: a key playmaker adipocytokine in non-alcoholic fatty liver disease. *Clin Exp Med* **14**, 121-131, doi:10.1007/s10238-012-0227-0 (2014).

26 Hu, R., Yang, X., He, X. & Song, G. The relationship between NAFLD and retinol-binding protein 4 - an updated systematic review and meta-analysis. *Lipids Health Dis* **22**, 8, doi:10.1186/s12944-022-01771-2 (2023).

27 Lopez-Mendez, I., Maldonado-Rojas, A. D. C., Uribe, M. & Juarez-Hernandez, E. Hunger & satiety signals: another key mechanism involved in the NAFLD pathway. *Front Endocrinol (Lausanne)* **14**, 1213372, doi:10.3389/fendo.2023.1213372 (2023).

28 Cao, W. *et al.* Serum Fibroblast Growth Factor 23 Level and Liver Fat Content in MAFLD: A Community-Based Cohort. *Diabetes Metab Syndr Obes* **14**, 4135-4143, doi:10.2147/DMSO.S328206 (2021).

29 Tian, H., Zhang, S., Liu, Y., Wu, Y. & Zhang, D. Fibroblast Growth Factors for Nonalcoholic Fatty Liver Disease: Opportunities and Challenges. *Int J Mol Sci* **24**, doi:10.3390/ijms24054583 (2023).

30 Kessoku, T. *et al.* Endotoxins and Non-Alcoholic Fatty Liver Disease. *Front Endocrinol (Lausanne)* **12**, 770986, doi:10.3389/fendo.2021.770986 (2021).

31 Li, Z. & Diehl, A. M. Innate immunity in the liver. *Curr Opin Gastroenterol* **19**, 565-571, doi:10.1097/00001574-200311000-00009 (2003).

32 Huby, T. & Gautier, E. L. Immune cell-mediated features of non-alcoholic steatohepatitis. *Nat Rev Immunol* **22**, 429-443, doi:10.1038/s41577-021-00639-3 (2022).

33 Petagine, L., Zariwala, M. G. & Patel, V. B. Non-alcoholic fatty liver disease: Immunological mechanisms and current treatments. *World J Gastroenterol* **29**, 4831-4850, doi:10.3748/wjg.v29.i32.4831 (2023).

34 Gadd, V. L. *et al.* The portal inflammatory infiltrate and ductular reaction in human nonalcoholic fatty liver disease. *Hepatology* **59**, 1393-1405, doi:10.1002/hep.26937 (2014).

35 Nishimura, S. *et al.* CD8+ effector T cells contribute to macrophage recruitment and adipose tissue inflammation in obesity. *Nat Med* **15**, 914-920, doi:10.1038/nm.1964 (2009).

36 Popov, Y. & Schuppan, D. CD8+ T cells drive adipose tissue inflammation--a novel clue for NASH pathogenesis? *J Hepatol* **52**, 130-132, doi:10.1016/j.jhep.2009.10.019 (2010).

37 Godfrey, D. I., MacDonald, H. R., Kronenberg, M., Smyth, M. J. & Van Kaer, L. NKT cells: what's in a name? *Nat Rev Immunol* **4**, 231-237, doi:10.1038/nri1309 (2004).

38 Tajiri, K. & Shimizu, Y. Role of NKT Cells in the Pathogenesis of NAFLD. *Int J Hepatol* **2012**, 850836, doi:10.1155/2012/850836 (2012).

39 Kremer, M. *et al.* Kupffer cell and interleukin-12-dependent loss of natural killer T cells in hepatosteatosis. *Hepatology* **51**, 130-141, doi:10.1002/hep.23292 (2010).
